# Site-Specific Immobilization of ZNRF3 Reveals the Importance of Target Structural Integrity on Macrocyclic Peptide Selections

**DOI:** 10.1021/acschembio.6c00015

**Published:** 2026-04-27

**Authors:** Demonta D. Coleman, Linnette Arceo, Armita Paydar, Alyssa Toner, Arya Kodali, Jacqueline Wright, J. Trae Hampton, Wenshe Ray Liu

**Affiliations:** † Texas A&M Drug Discovery Center and Department of Chemistry, 14736 Texas A&M University, College Station, Texas 77843, United States; ‡ Institute of Biosciences and Technology and Department of Translational Medical Sciences, College of Medicine, Texas A&M University, Houston, Texas 77030, United States; § Department of Biochemistry and Biophysics, Texas A&M University, College Station, Texas 77843, United States; ∥ Department of Cell Biology and Genetics, College of Medicine, Texas A&M University, College Station, Texas 77843, United States; ⊥ Department of Pharmaceutical Sciences, Texas A&M University, College Station, Texas 77843, United States

## Abstract

Phage display of macrocyclic peptide libraries has proven highly effective for ligand discovery, yet the impact of target immobilization and structural integrity on selection outcomes has not been systematically examined. Using the ZNRF3 ectodomain as a model, we incorporated p-azidophenylalanine (AzF) at three phenylalanine residues with distinct solvent exposures (F217, F85, F156) to enable selective perturbation of the protein’s structure via site-specific strain-promoted azide–alkyne cycloaddition (SPAAC) immobilization. Biochemical evaluation of the mutants confirmed efficient conjugation and structural disruption of the protein, with the F156AzF mutant displaying the most significant reduction in activity. Phage selections using a CX_12_C macrocyclic library demonstrated that enrichment efficiency and sequence diversity correlated with structural preservation: F217AzF and F85AzF yielded robust and overlapping peptide pools, while F156AzF produced few and modestly enriched sequences. Biophysical characterization of top hits indicated that peptides derived from structurally intact immobilizations were most likely to bind wild-type ZNRF3, with the highest-affinity ligand, 85–2 (*K*_D_ = 124 nM), emerging from the shared pool. This work reports a technique to selectively disrupt protein domains during phage selections, while also demonstrating that the structural integrity of immobilized targets is a primary determinant of phage display success. Not only does the necessity to maintain structural integrity influence sequence composition and affinities of peptides toward native protein targets, but also the overall enrichment efficiency of the selection itself. While disruptive immobilization may still yield useful ligands, strategies that preserve native folds enhance phage enrichment and maximize the identification of biologically relevant binders.

## Graphical abstract

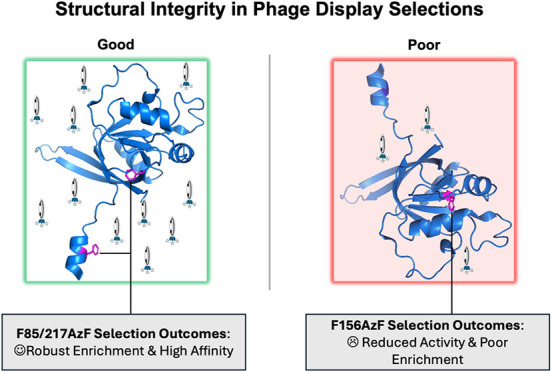

## Introduction

Macrocyclic peptide libraries displayed on filamentous phage have emerged as fertile sources of high-affinity ligands for challenging protein targets.^1,2^ Although downstream hit-to-lead optimization is now routine, the earliest step in any biopanning campaign—the way in which a protein target is immobilized on a solid support—remains poorly examined. The immobilization chemistry dictates which epitopes are accessible, which off-targets are copresented, and which conditions preserve the native fold of the protein.^2,3^ Together, these factors profoundly influence both the enrichment process and the biochemical relevance of discovered binders. Conventional immobilization strategies include immunocapture, biotin–streptavidin capture, and direct covalent coupling.^4^ Immunocapture is typically achieved using antibodies or antibody fragments that coat beads or plates for an affinity tag such as FLAG, His-tag, HA, or GST.^5^ For biotin–streptavidin capture, biotinylation of a protein target can be accomplished site-specifically on a genetically fused Avi-tag or nonselectively on lysine residues using the NHS-biotin reagent, and then streptavidin-coated beads or plates are used to capture the biotinylated target.^4,6^ In both methods, coating proteins on beads or plates that are used for target capture are off-targets and can potentially lead to false-positive hits. Moreover, random lysine biotinylation can perturb tertiary structure, especially when essential lysines participate in salt bridges or ligand binding. Direct covalent coupling uses NHS-activated beads or plates, allowing direct amide bond formation with lysine-side-chain amines of a target protein.^7,8^ This strategy obviates secondary protein reagents for target capture to avoid off-target effects. However, the approach itself is fundamentally “structure-blind,” and cross-linking at surface lysines can distort protein epitopes or fix the target in non-native orientations, reducing the likelihood that selected peptides will recognize the protein in its native state. Therefore, there is a structural integrity concern for the protein target used in phage display. However, despite more than three decades of phage display applications, there has been no systematic study to quantify how the structural integrity of a protein target influences the identity, affinity, and specificity of macrocyclic peptide ligands. Here, we attempt to address this gap.

ZNRF3 is a single-chain transmembrane E3 ligase whose extracellular domain binds R-spondins, while its intracellular RING domain ubiquitinates Frizzled and LRP5/6 receptors.^9,10^ This topology, with an accessible ectodomain coupled to a catalytically active cytosolic tail, makes ZNRF3 an ideal recruitable ligase for antibody-based proteolysis-targeting chimeras (AbTACs), a concept that has been demonstrated.^11,12^ We selected the ectodomain of ZNRF3 as a model protein target to investigate the impact of target structural integrity on phage display-based ligand selection, as this domain has previously been used successfully to identify macrocyclic peptide ligands that bind ZNRF3.^13,14^ For site-specific immobilization that allows precise assessment of structural perturbation to the ZNRF3 ectodomain, we employed genetic code expansion to incorporate p-azidophenylalanine (AzF) at three distinct phenylalanine residues, enabling copper-free strain-promoted azide–alkyne cycloaddition (SPAAC) with dibenzocyclooctyne (DBCO)-functionalized magnetic beads.^15,16^ The three sites—F217, F85, and F156—as shown in Figure 1, were selected based on their solvent accessibility. F217 is fully exposed on a flexible loop, F85 is partially solvent-exposed but with its side chain buried for interactions with other residues, and F156 is fully buried within the protein’s folded core.^9^ As such, conjugation at these sites is expected to have different effects on ZNRF3 ectodomain structural integrity. F217AzF is anticipated to react with minimal perturbation to the protein structure due to its loop flexibility; F85AzF will need partial structural rearrangement to expose F85AzF and allow DBCO access; and F156AzF, deeply buried and located in a structurally constrained region, will require significant disruption to the protein structure to flip it out for conjugation with DBCO, potentially destabilizing the overall fold. Because F85AzF and F156AzF require conformational shifts to accommodate the SPAAC reaction, we hypothesize that the extent of structural distortion will correlate with the quality and relevance of ligands discovered through phage display when they are used for phage enrichment. In particular, the F156AzF mutant is expected to yield fewer functional ligands that bind the native ZNRF3 structure. Below, we present our phage selection results using these three site-specifically immobilized ZNRF3 ectodomain variants.

**Figure 1:**
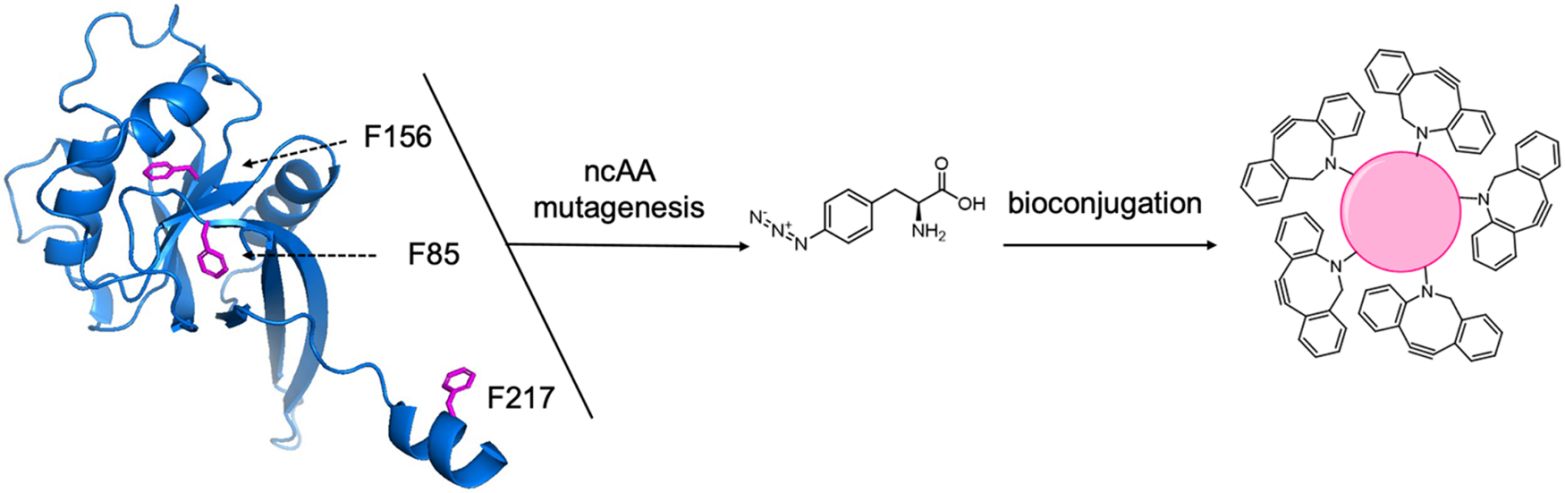
Site-specific incorporation of p-azidophenylalanine (AzF) into the ZNRF3 ectodomain via amber codon mutagenesis. Residues F85, F156 (magenta), and F217 (illustrated) were selected based on varying solvent exposure to probe structural integrity and enable subsequent copper-free click chemistry-based bioconjugation to DBCO-coated magnetic beads. The protein representation was extracted from the AlphaFold Protein Database (AF-Q9ULT6-F1-v4).

## Results and Discussion

### Production and Validation of Mutant ZNRF3 Ectodomain Proteins

To generate the AzF mutant ZNRF3 ectodomain proteins, amber (UAG) codons were introduced at sites F85, F156, and F217 in three distinct plasmids expressing the ectodomain of ZNRF3 to evaluate varying degrees of structural integrity probing. (Figure 1; amino acids 56–219). AzF incorporation was then performed using a previously developed AzPheRS/mutRNA tRNA synthetase/tRNA pair originally derived from the *Methanococcus jannaschii* tyrosyl-tRNA synthetase (MjTyrRS).^15^ All constructs gave high protein expression of the mutants, and high-resolution mass spectrometry (HRMS) experiments of the purified ZNRF3 mutants confirmed intact AzF in the isolated proteins (Figures S1–S3). As a control protein, we also expressed and purified wild-type ZNRF3 with an Avi-tag fused to the C-terminal domain for immobilization during analysis (Figure S4).

With the AzF mutants in hand, we sought to evaluate their reactivity with DBCO and structural integrity prior to performing phage biopanning experiments. In the initial assay, the protein samples were diluted in assay buffer to 25 μM and incubated overnight at RT with a 3-fold molar excess of commercially available fluorescein-DBCO. Analysis by fluorescent SDS-PAGE revealed that each AzF mutant produced a distinct fluorescent band at the expected molecular weight when compared to the wild-type, and HRMS on samples exchanged into ammonium bicarbonate buffer confirmed the addition of a single fluorescein-DBCO moiety to only the mutant proteins, with the observed mass corresponding exactly to the expected mass (Figures S5,S6). Although the wild-type protein showed minimal labeling on the fluorescent SDS-PAGE gel, likely due to nonspecific interactions between DBCO and accessible cysteine or lysine residues during the boiling step, the mutants displayed markedly enhanced fluorescent signals, indicating the dependence on AzF for labeling. To further examine the reactivity profile of each mutant, dose–dependency experiments were performed by reacting 25 μM of protein with varying concentrations of fluorescein-DBCO (0, 1, 5, 25, and 50 μM) under identical conditions. Fluorescent imaging was conducted before Coomassie Blue staining, and the quantification of the band intensity ratios provided a comparative assessment of reactivity across the different mutants and wild-type (Figure 2A). The integrated analyses from SDS-PAGE and HRMS established the relative reactivity order as follows: F217AzF > F156AzF > F85AzF ≫ wild-type. Collectively, these detailed evaluations confirm the reactivity of the AzF handle and underscore the feasibility of using SPAAC for controlled modulation of the ZNRF3 mutant proteins.

**Figure 2:**
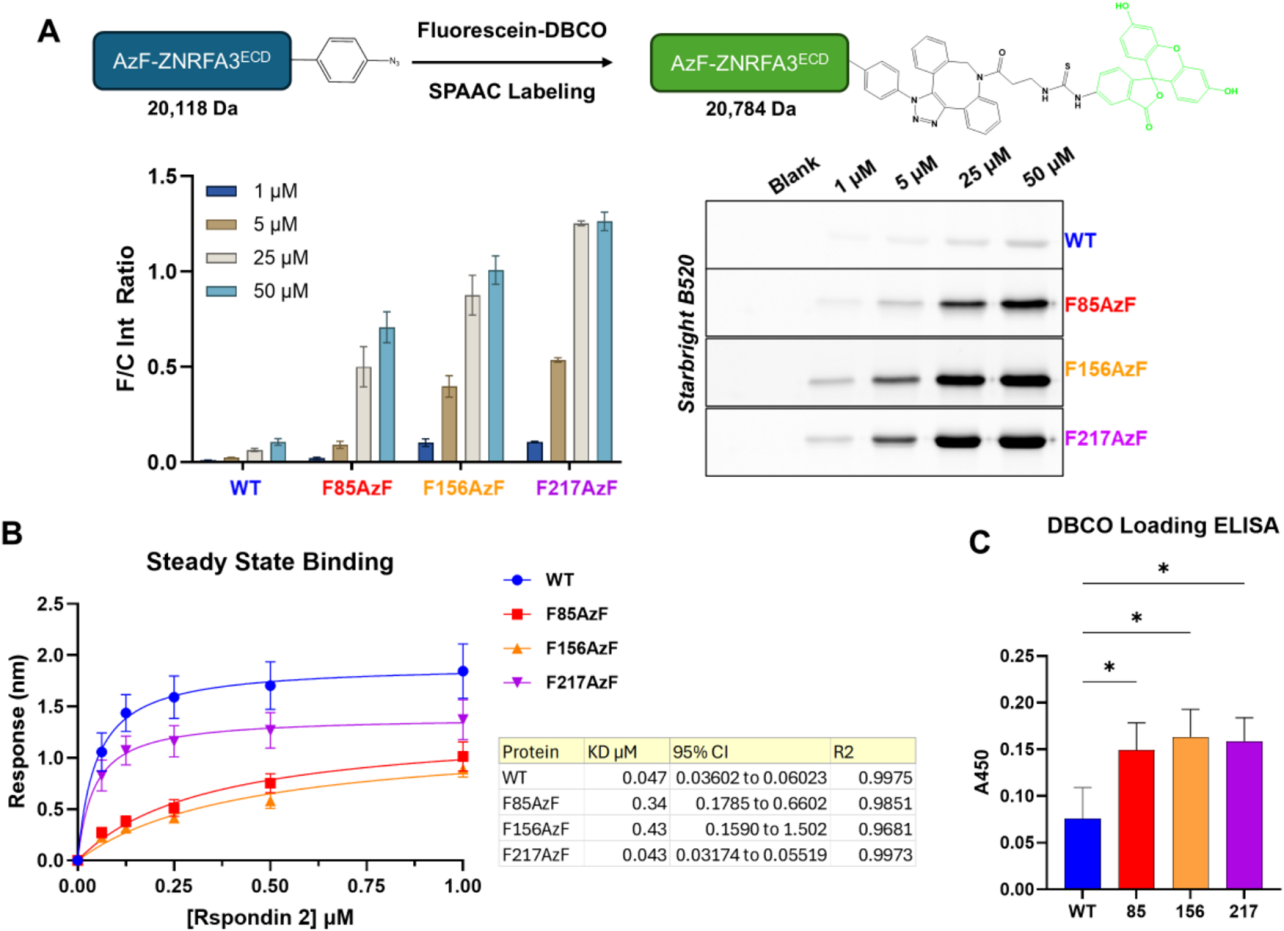
Wild-type (WT) ZNRF3 and single p-azido-l-phenylalanine (AzF) mutants (F85AzF, F156AzF, F217AzF) assessed for click-mediated conjugation and R-spondin 2 binding. (A) Fluorescein–DBCO SPAAC labeling, quantified as fluorescence-to-Coomassie band intensity ratios in ImageJ, revealed site-dependent differences in labeling efficiency. (B) Steady-state binding of WT and AzF mutants to R-spondin 2 was fitted to a nonlinear one-site specific binding model. The table reports calculated dissociation constants (*K*_d_), 95% confidence intervals, and goodness-of-fit (*R* ^2^). WT and F217AzF exhibited similar affinities, whereas F85AzF and F156AzF showed significantly reduced binding. (C) Click-mediated DBCO magnetic bead loading, quantified by ELISA at A450, demonstrated significant differences among mutant loading when compared to WT by one-way ANOVA (*p < 0.05). All experiments in performed in triplicate.

Following establishment of the reactivity of AzF in the expressed proteins, we then looked to test whether modulation of each site would cause perturbations to ZNRF3 structural integrity. Following SPAAC reactions of each mutant with DBCO-PEG12-biotin, their interaction with a commercially purchased R-spondin 2 (RSPO-2) was assessed using biolayer interferometry (BLI) with streptavidin charged sensors (Figure S7). As expected, the wild-type ZNRF3 protein gave high-binding to RSPO-2, with an observed *K*_D_ of 47 nM. The F217AzF mutant also demonstrated similarly high binding affinity toward RSPO-2, resulting in a *K*_D_ of 42 nM and kinetic curves that were similar to the wild-type protein (Figure 2B). However, mutants F85AzF and F156AzF exhibited significantly reduced affinity for RSPO-2 with steady-state *K*_D_ values of 340 (∼7 fold increase) and 430 nM (∼9 fold increase), respectively, along with dose–response kinetic profiles that indicated a similar affinity trend toward RSPO-2 (Figures 2B and S8). These data further corroborate the initial hypothesis that introduction of AzF mutants within the core folding regions of the ZNRF3 ectodomain would result in controlled perturbations of structural integrity. To provide direct structural evidence for the integrity of the ZNRF3 ectodomain variants, we collected far-UV circular dichroism (CD) spectra for WT and the three single-site AzF mutants (Figure S9). The spectra exhibited highly similar overall shapes across 190–250 nm, consistent with preservation of global secondary-structure architecture across constructs. Secondary-structure deconvolution (**BeStSel**) yielded good fits for all samples (NRMSD 0.016–0.020), supporting that each variant remains folded under the measurement conditions; detailed secondary-structure percentages are reported in Table S1.^17^ Importantly, none of the mutants displayed spectral features consistent with extensive unfolding or aggregation. However, the most extensive changes were observed in the F156AzF mutant that were consistent with disruption of the expected β-sheet core. Together, these data indicate that AzF incorporation does not grossly destabilize the ZNRF3 ectodomain in solution, rather suggesting that the reduced R-spondin 2 binding observed for F85AzF and F156AzF (Figure 2B) reflected the localized perturbations to the core that could have been amplified following immobilization/conjugation. As such, we believed these mutants would be good candidates to begin the phage biopanning experiments exploring the relationship between selection results and protein structural integrity.

### Phage Biopanning of ZNRF3Mutants

Prior to biopanning experiments, we first ensured immobilization of the ZNRF3 mutants could be achieved through reactivity with the AzF handles. To evaluate efficiency of the SPAAC immobilization, the purified AzF mutants were incubated with DBCO-functionalized magnetic beads at 4 °C overnight. The low-temperature incubation was chosen to minimize protein degradation and preserve native conformation. After bead conjugation and washing, bound protein was detected via an HRP-conjugated anti-ZNRF3 antibody in a modified ELISA format (Figure 2C). Absorbance measurements revealed statistically significant increases of loading of all AzF-containing mutants onto the beads when compared to the wild-type control. This corresponds well with our previous fluorescence labeling data, which investigated the reactivity of the AzF residues in each mutant. Overall, these experiments further validated the SPAAC-based site-directed conjugation strategy and encouraged us to continue with phage biopanning.

Following immobilization of the mutant ZNRF3 proteins onto the DBCO resin, a disulfide-cyclized CX_12_C phage library was used to identify cyclic peptides that bind to each protein (Figure 3A). Three independent biopanning experiments were conducted using the different AzF mutants, of which, when bound, should result in distinct orientations on the beads. Each selection process involved three rounds of biopanning, each incorporating a negative selection step to eliminate phages that bound nonspecifically to the resin. Phage titers to estimate the percent of phages binding to the protein increased sequentially, indicating successful enrichment of peptides throughout the selection (Figure 3B). Following the third round of biopanning, phagemids from each round were isolated and amplified for next-generation sequencing to detect differences in the composition of enriched libraries. Differential enrichment analysis was performed on the peptides identified after the final round of selection for each mutant. Interestingly, there was a clear relationship between the overall enrichment of peptides and the structural integrity of the protein target. The number of sequences with abundances over 0.1% of total sequences correlated with the structural integrity of the target, with 45, 27, and 4 peptides identified in the F217AzF, F85AzF, and F156AzF mutants, respectively (Figure 3C). F156AzF, the mutant with the lowest structural integrity, gave considerably lower enrichment than the other mutants, with over 80% of its sequences being found in low abundance (less than 10 copies) in the final round of selection. Mutants F217AzF and F85AzF, on the other hand, displayed enhanced enrichment profiles that were similar to each other (Figure S10). While phage selections should, in theory, be able to enrich peptides regardless of the structure of the target, our data indicate that the success of overall enrichment actually depends on the structural integrity of the target. To further benchmark our site-specific AzF-based immobilization, we also performed three rounds of selection against wild-type ZNRF3 ECD immobilized via lysine residues on NHS-activated magnetic beads following the manufacturer’s recommended protocol. In round 3 of this NHS-based selection, the library exhibited a comparatively diffuse enrichment profile, with only 4 peptide sequences above 0.1% abundance out of approximately 5 × 10^4^ unique sequences, closely resembling the low-enrichment behavior observed for the structurally compromised F156AzF mutant (Figure S11). This similarity suggests that both reduced target integrity and nonsite-specific lysine immobilization are associated with less focused convergence onto a small set of high-abundance binders under our conditions.

**Figure 3:**
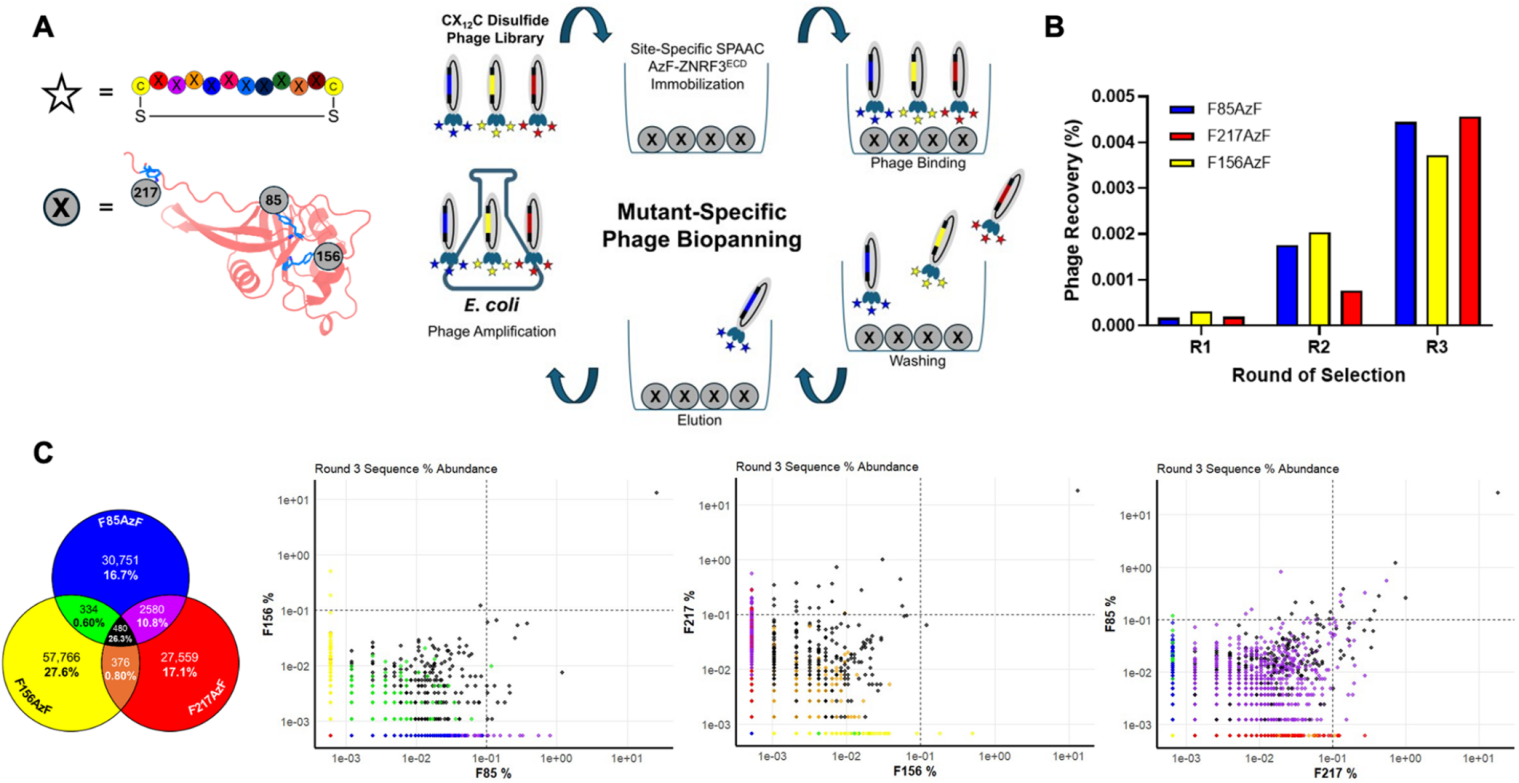
Mutant-Specific Macrocyclic Peptide Phage Display Selections against AzF Mutants. (A) Schematic of phage display biopanning against AzF-containing ZNRF3 mutants with orientation-specific immobilization. (B) Phage elution percentages across three rounds of selection for each mutant, showing progressive enrichment. (C) Illumina next-generation sequencing of round 3 peptide pools, visualized as a triple Venn diagram indicating mutant-specific and shared sequences (percentages shown relative to all reads). Corresponding scatter plots illustrate sequence enrichment from round 1 to round 3, with dashed lines marking the 0.1% abundance threshold.

Having established that the enrichment profiles were related to the structural integrity of the ZNRF3 mutants, we then looked to see how this affected the abundance of particular sequences within each sample. Out of 252,521 total reads among the three different selections, 124,096 unique peptide sequences were identified (Figure S10). Similar to the enrichment data, there was considerable overlap in sequence identities between the F85AzF and F217AzF mutants, with 2580 sequences shared between the two samples that made up over 10% of the entire sequencing population (Figure 3C). On the other hand, overlap with the F156AzF mutant was much lower, consisting of only 334 peptides (0.6% overall abundance) and 376 peptides (0.8% overall abundance) shared between the F85AzF and F217AzF mutants, respectively. Despite the low prevalence of overlap of F156AzF with either sequence, 480 sequences were found in all three samples, making up 26.3% of the total sequenced population and the top sequence in each sample was the same. Looking at the sequence composition of the hits above 0.1% abundance in round 3 for the F217AzF selection, 20/45 peptides were common to all samples and 20/45 were shared uniquely with the F85AzF mutant, whereas only 4 were unique to the F217AzF mutant (Table S1). In the F85AzF selection, 16/27 of the top hits were common to all, 10 were shared only with the F217AzF mutant, and none were unique to the F85AzF. In the four hits above 0.1% of the F156AzF mutant, two were common to all selections and the others were unique to the F156AzF; notably, the NHS-based wild-type ZNRF3 selection also displayed a predominance of low- and intermediate-abundance sequences with very few peptides exceeding 0.1% abundance in round 3, mirroring the diffuse enrichment profile observed for F156AzF (Figure S11). Considering these data, key deviations from a predicted, randomized enrichment between mutants occurred in two main areas: the overabundance of common sequences and the reduced overlapping of the F156AzF mutant with others (Figure S12). This further indicates a unique relationship in the structures of the F217AzF and F85AzF mutants in comparison to the F156AzF mutant. The lack of overlap with the F156AzF mutant seemed reasonable due to its low structural integrity. However, further exploration of the individual peptide sequences was warranted to better understand the differential enrichment patterns in the selection.

### Characterization of Peptide Hits for Binding ZNRF3

Following identification of hit sequences using next-generation sequencing, individual peptides representing a variety of groups within the analysis were then characterized for binding to the wild-type ZNRF3 ectodomain. For a general peptide BLI survey, the top five peptide sequences with the highest abundances in each mutant group were synthesized using solid-phase peptide synthesis and purified using high-performance liquid chromatography prior to testing (Figures S13–S49 and Tables S2–S4). The peptides were then characterized for interactions with ZNRF3 using BLI, and those shown in Figure S13A,B correspond to peptides that bound to wild-type ZNRF3 while those shown in Figure S14 correspond to nonbinders. First, we noticed peptides selected from the F85AzF and F217AzF mutants showed a higher likelihood of binding the wild-type ZNRF3, with 2/5 and 3/5 sequences, respectively, exhibiting measurable affinity. On the other hand, the top five peptides from the F156AzF mutant produced the fewest binders, with only 1/5 showing affinity toward ZNRF3. This is likely due to greater structural disruption in the F156AzF mutant compared to the other two mutants. To broaden the BLI analysis, we selected the two most abundant sequences from each Venn diagram category and grouped them according to their overlap region prior to testing against wild-type ZNRF3 (Figure 4A).

**Figure 4:**
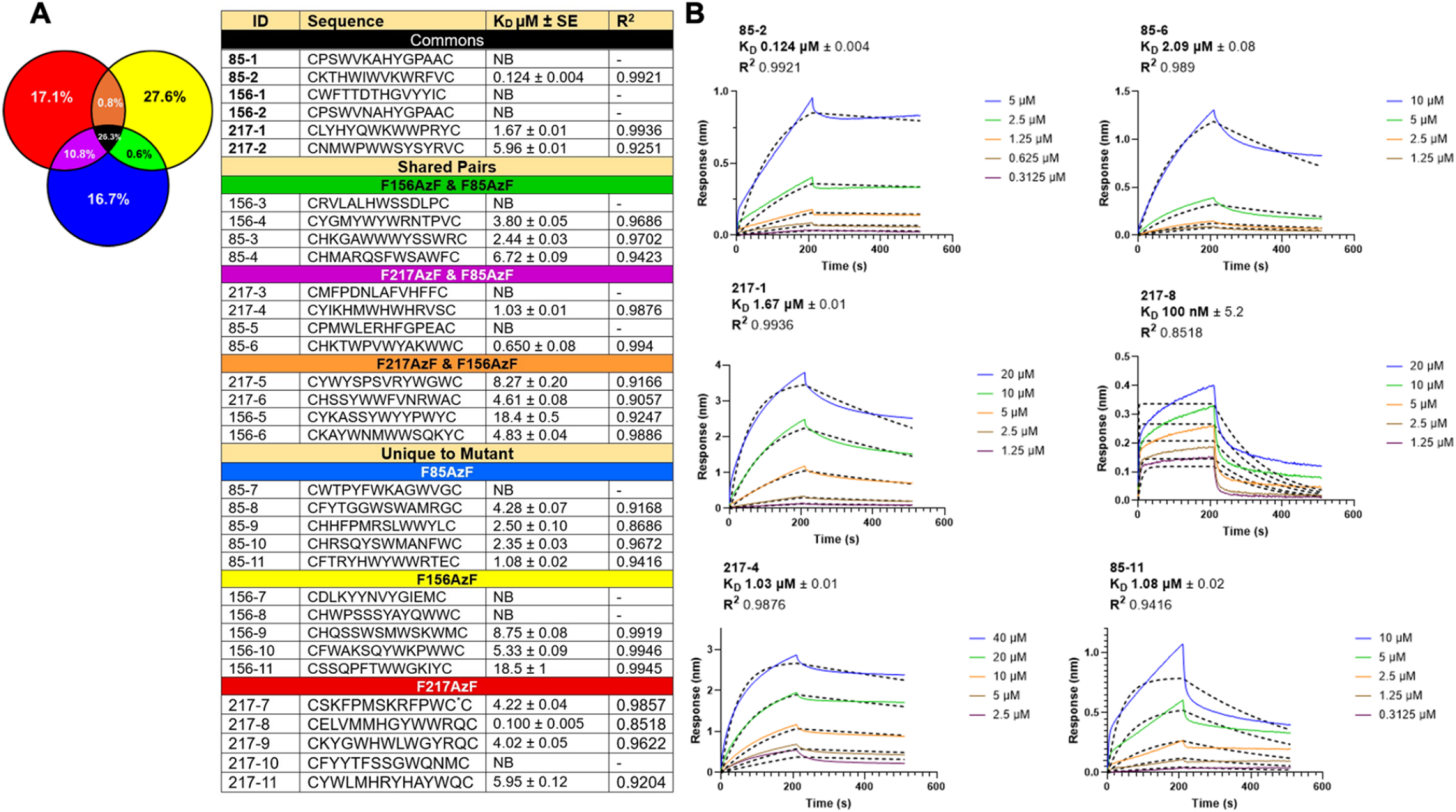
(A) Venn diagram and associated table showing peptide sequence overlap across F85AzF, 217AzF, and F156AzF selections. Categories are color-coded: Common (black), F85/156AzF shared (green), F85/217AzF shared (purple), 217/156 shared (orange), F85AzF unique (blue), F156AzF unique (yellow), and 217 unique (red). The two most abundant sequences from each category were selected for BLI analysis. Table summarizes peptide ID, sequence, dissociation constant (*K*_d_ ± SE), and *R* ^2^ values. C* = Abu as this kept disulfide positioning consistent across all peptides tested. (B) Representative BLI sensorgrams for peptides with *K*_d_ values ≤ 2 μM, underscoring the role of mutant structural integrity in shaping the recovery of high-affinity binders. Raw traces (multicolored by concentration) were fitted (black dotted lines) to a 1:1 protein/ligand binding model to calculate kinetic parameters.

We hypothesized that there may also be a strong chance of identifying binders within the sequences common to all three mutants, since these corresponded to the peptide sequences with the highest abundance in each selection. BLI studies are summarized in Figure 4, with representative curves for high affinity peptides and low affinity/nonbinders being found in Figures 4B and S13, respectively. While only 3/6 of these peptides bound to the protein, the highest affinity peptide of all mutants was found in this section, with a *K*_D_ of 124 nM (Figure 4B). Unexpectedly, the most enriched sequence was observed to be a nonspecific binder, likely enriched through binding to the plastic or resin scaffold during the selection. This led us to conclude that, even when performing negative selections, sequences commonly found in all three mutants were equally likely to be derived from either nonspecific artifacts present during the selection or from actual interactions with ZNRF3. However, looking at the overlapping sequences between two mutants alone seemed to mitigate the propensity to identify nonselective binders, where 9/12 peptides in this class exhibited binding toward the wild-type ZNRF3. As expected, F85AzF and 217AzF produced the most binders with 4/5 peptides in their top 5 unique-to mutant sequences, most of which had *K*_D_ values under 5 μM. For F156AzF, there were only 3/5 peptides that bound wild-type in this subset, but the affinities of these peptides were lower in comparison. These data suggest a rather complex relationship between peptide abundance, structural integrity of the target, and overlapping regions between different selections that make it difficult to predict binders from nonbinders. In future studies, it may be useful to employ larger-scale peptide surveys in combination with machine learning to better predict peptide binders directly from next-generation sequencing data. Nonetheless, the general trend in the unique sequences identified from each mutant is consistent with our previous observations on enrichment, in which proteins with higher structural integrity are more likely to produce productive binders during a phage selection.

## Conclusions

In summary, our side-by-side selections demonstrate that target structural integrity is one of most significant determinants of phage display success. Site-specific conjugation at the fully buried residue F156AzF produced a markedly smaller set of functional macrocyclic peptides that recognize the native ZNRF3 fold than conjugation at the solvent-exposed, loop-localized F217AzF or partially exposed F85AzF, both of which preserve the folded core. Our results confirm that structural disruption of a protein target negatively impact phage display and sharply reduces the probability of enriching functional ligands. Nonetheless, a handful of potent binders were discovered even from the highly perturbed F156AzF variant, indicating that severe immobilization artifacts do not abolish, but merely, diminish, the chance of identifying functional ligands. Therefore, the practical message from this study is 2-fold. It is optimal to choose immobilization strategies that preserve native structure and a site-specific modification strategy allows precise structural perturbation assessment; when such strategies are not available, more disruptive methods can still yield useful hits, provided that downstream validation is rigorous and expectations of hit frequency are adjusted accordingly. This conclusion parallels a long-standing practice in antibody discovery, where peptide immunogens, which are typically unstructured and therefore structurally decoupled from their parent proteins, often elicit binders that require extensive screening to find those that recognize the full-length antigen. Likewise, peptide fragment-based phage selections should be used with caution, as they are functionally analogous to the F156AzF scenario where sequence identity alone is not a guarantee of structural fidelity and the cost is a lower yield of truly functional ligands.

Although this study uses the ZNRF3 ectodomain as a model system, the central observation that the structural integrity of an immobilized target can be a primary determinant of enrichment efficiency and downstream binder relevance is expected to generalize to other protein classes in which immobilization can bias orientation, surface accessibility, and conformational stability^18,19^ In practice, additional target specific factors are likely to modulate the severity of immobilization artifacts, including protein size, intrinsic folding stability, and surface charge distribution, each of which can influence multipoint attachment, nonspecific adsorption, and the energetic cost of presenting a reactive handle at the surface.^18^ Larger or less stable proteins may be more susceptible to partial unfolding upon tethering, whereas highly charged proteins may exhibit stronger electrostatic interactions with surfaces that alter presentation of functional epitopes.^18,20^ Future applications of this strategy could therefore benefit from incorporating orthogonal stability readouts and from selecting immobilization sites and chemistries that minimize perturbation while maintaining a controlled orientation.

## Supplementary Material



## Notes

Additional experimental details; additional sequencing analysis; characterization of peptides; and R scripts can be found in the attached Supporting Information (PDF)
