# Supplementary material for: Site-Specific Immobilization of ZNRF3 Reveals the Importance of Target Structural Integrity on Macrocyclic Peptide Selections
Source: ACS Chem Biol. 2026 Apr 27;21(5):1038–44. doi: 10.1021/acschembio.6c00015 (PMC13184931; doi:10.1021/acschembio.6c00015)
Supplement: Supplementary file 1 [file cb6c00015_si_001.pdf]

SUPPORTING INFORMATION

Site-Specific Immobilization of ZNRF3 Reveals the Importance of Target Structural Integrity on Macrocyclic Peptide Selections

Demonta D. Coleman[a], Linnette Arceo[a], Armita Paydar[a], Alyssa Toner[a], Arya Kodali[a], Jacqueline Wright[a], J. Trae Hampton[a]*, and Wenshe Ray Liu[a,b,c,d,]*

[a]Texas A&M Drug Discovery Center and Department of Chemistry, Texas A&M University, College Station, TX 77843, USA

[b]Institute of Biosciences and Technology and Department of Translational Medical Sciences, College of Medicine, Texas A&M University, Houston, TX 77030, USA

[c]Department of Biochemistry and Biophysics, Texas A&M University, College Station, TX 77843, USA

[d]Department of Cell Biology and Genetics, College of Medicine, Texas A&M University, College Station, TX 77843, USA

[e]Department of Pharmaceutical Sciences, Texas A&M University, College Station, TX 77843, USA

*Correspondence should be addressed to Wenshe Ray Liu and J. Trae Hampton: wsliu2007@tamu.edu, jhampton1@tamu.edu

METHODS

Site-Directed Mutagenesis Primer List

1) F85TAG-For:

CTTACGGGTCGTTAGTCACGCGCTG

2) F85TAG-Rev:

CAGCGCGTGACTAACGACCCGTAAG

3) F156TAG-For:

CGGCAGTCATTTAGGATGTATCAG

4) F156TAG-Rev:

CTGATACATCCTAAATGACTGCCG

5) F217TAG-For:

CCGACAGAATATTAGGATATGGGTTTA

6) F217TAG-Rev:

TAAACCCATATCCTAATATTCTGTCGG

NGS Primers List

7) NGS-F1:

5’TCGTCGGCAGCGTCAGATGTGTATAAGAGACAGGCCCAGCCGGCCATG3’

8) NGS-R1:

5’GTCTCGTGGGCTCGGAGATGTGTATAAGAGACAGCGGCCGCTTTCGCCGC3’

5) NGS-i7: 5’CAAGCAGAAGACGGCATACGAGAT[i7]GTCTCGTGGGCTCGG3’

6) NGS-i5: 5’AATGATACGGCGACCACCGAGATCTACAC[i5]TCGTCGGCAGCGTC3’

Plasmid Construction

pET28a-His$_6$-SUMO-ZNRF3^{ECD}(AA:56-219)/ FXTAG-Avi

The *ZNRF3*^{ECD} FXTAG mutations were generated in the *pET28a-His$_6$-SUMO-ZNRF3-Avi* plasmid via site-directed mutagenesis. The primers used to do so are listed above. The primers were used to mutate phenylalanine at the specified positions in the ZNRF3(AA: 56-219) gene. The template was mutated using a one-step gradient PCR (30 cycles with annealing at 62 °C). The PCR products were treated with DpnI at 37 °C for 1 h, then gel extracted using a 1% agarose gel and a commercially available purification kit (Gencatch). The purified products were then transformed

into Top10 Chemical Competent cells (ThermoFisher) and clones were verified with Sanger sequencing.

pADL-(NNK)$_{12}$-gIII

The plasmid *pADL-(NNK)$_{12}$-gIII* was previously constructed and transformed into electrocompetent E. coli ER2738, yielding 1.8 x 10^9 transformants.[14]

Expression of *ZNRF3*^{ECD} FXAzF Mutants

Plasmids *pET28a-His6-SUMO-ZNRF3*^{ECD}-FXTAG*/(AA:56-219)-Avi* and *pAzF* (gift from Peter Schultz, Addgene plasmid # 31186) were cotransformed into E. coli (BL21). The cells were then inoculated into 2xYT containing 50 µg/mL kanamycin and 34 µg/mL chloramphenicol and grew at 37 °C until OD = 0.5. Expression was then induced by media supplementation with 1 mM IPTG, 2.5 mM 4-Azido-L-phenylalanine hydrochloride, and 0.2% arabinose. The protein was expressed for 18 h at 16 °C. The cells were then pelleted (6000 x rpm, 15 min) and then resuspended in buffer A (50 mM Tris-HCl, 10% glycerol, 150 mM NaCl, 25 mM imidazole, pH 7.5, 2 mL/100ml of expression). The cells were then lysed by sonication (3 s on, 3 s off, 6 s, 65% A) and the lysate was clarified by centrifugation (17000 x g, 20 min). The supernatant was then incubated with a Ni-NTA column for 1 hour at 4 °C. The column was then washed with 5 CV of buffer A before the addition of 30 µg/mL 6xhis-tagged SUMO protease. The protein was cleaved overnight on the Ni-NTA column at 4 °C. The sumo-cleaved protein was collected by polling the initial flowthrough and a 2 CV column rinse. The FXAzF mutants were concentrated to 1 mL by centrifugation before being used for assays. ZNRF3 ECD wild-type expression followed the same steps, except induction proceeded with only IPTG due to the absence of the pAzF plasmid in the expressing cells.

Click-promoted Fluorescein-labeling

The AzF mutants or wild-type protein were diluted to 25 µM (0.5 mg/ml) in buffer A. After the dilution, 1, 5, 25, 50, or 75 µM fluorescein-DBCO (Aurum Pharmatech) was added, and the reaction solution was left at 4 °C or room temperature overnight with shaking. The next day, the reaction solutions were either exchanged into ammonium bicarbonate buffer for high-resolution mass spectrometry or quenched by adding 4xLaemmle SDS sample buffer (ThermoFisher) and boiling for 5 min. The fluorescent SDS-PAGE-specific samples were loaded

onto a 15% Bis-Tris gel and ran for 15 min at 120V followed by 30 min at 180V. The resulting gels were imaged with Starbright B520 using a Bio-Rad ChemiDoc before being subjected to Coomassie blue staining and imaging. Band intensities (fluorescence or Coomassie) were quantified using Image J software.

Circular dichroism spectroscopy

Far-UV CD spectra were acquired on a JASCO J-1500 spectropolarimeter using a 1 mm pathlength quartz cuvette at 25 °C in 10 mM phosphate, 50 mM NaF (pH 7.5). Spectra were collected from 250–190 nm and recorded as the mean of four technical replicate scans for each construct, with buffer baselines acquired under identical conditions and subtracted during data processing. The resulting spectra were converted to Δε (M^{-1} cm^{-1}) using the measured protein concentration and pathlength and analyzed using BeStSel for secondary-structure estimation (NRMSD reported as a fit-quality metric).[17]

Click-promoted Biotin Labeling for Biolayer Interferometry against Rspondin-2

The AzF mutants were diluted to 25 µM (0.5 mg/ml) in buffer A. EZ-Link™ Biotin-PEG12-DBCO (Thermo Fisher) was added to 200 µM, and the reaction was left to incubate at 30°C overnight. The samples were then desalted with buffer A using a 10k MWCO Amicon spin filter (Millipore) to remove unreacted biotin-PEG12-DBCO. The resulting samples were either used directly for BLI streptavidin sensor loading for Rspondin 2 (Thermo Fisher) affinity assessment or exchanged into ammonium bicarbonate buffer for HRMS analysis.

DBCO Bead-Loading ELISA

Dibenzocyclooctyne (DBCO)- coated magnetic beads (10 µL, 10mg/mL slurry, Kerafast) were placed in a 1.5 mL tube and washed three times with 1 mL of buffer A. Next, 60 µg/ml of the FXTAG mutants or wild-type protein in 250 µL of buffer A was added to the washed magnetic beads. The samples were allowed to react while shaking for three hours at 4°C. After the reaction, the tubes were positioned on a magnetic rack, and the supernatants were discarded. The beads were washed three times with 1 mL of PBST (phosphate-buffered saline with 0.05% Tween 20) before adding 1 mL of blocking buffer (5% BSA/PBST) to the tubes. The beads were left to block for 45 minutes with end-over-end agitation at room temperature. Following

blocking, the supernatants were removed as before, and the anti-ZNRF3 HRP antibody (Epigentek) was diluted (1:5000) in PBST and added to each tube for a 45-minute incubation at room temperature. The supernatants were removed, and the beads were washed five times with PBST before adding 100 uL of 1-Step™ TMB ELISA substrate solution (ThermoFisher). The bead/substrate solutions were incubated for 20 min. The supernatants were added to a 96-well plate before the stop solution (1M sulfuric acid) was added to each well. The absorbance at 450 nm was measured with a Synergy Neo2 plate reader (Biotek). Reactions were done in triplicate.

Phage Expression and Purification

E. coli ER2738 containing the pADL-NNK$_{12}$-gIII or pADL-CA$_5$C-gIII phagemid library were grown at 37°C in 250 mL of 2xYT media supplemented with 100 µg/mL ampicillin, 10 µg/mL tetracycline, and 1% glycerol. Upon reaching OD_{600} = 0.5-0.6, 20 mL of the culture of was transferred to a small flask and infected with 20µL of CM13d3 helper phage (MOI > 5, Antibody Design Labs, San Diego, CA) at 37°C with shaking. After 45 min the cells were pelleted (3750 x*g*, 15 min) and resuspended in 200 mL of 2xYT media containing 10 µg/mL tetracycline, 100 µg/mL ampicillin, 25 µg/mL kanamycin, and 1 mM IPTG and incubated at 30 °C. 18 h post induction, the culture was transferred to 50 mL tubes, cells were pelleted (3750 x*g*, 20 min), and the supernatant was decanted into new tubes. Phages were precipitated by the addition of appropriate amounts of 5x Precipitation Buffer (20% polyethylene glycol 8000, 2.5 M NaCl) to afford a 1x solution, then incubated at 4 °C for 1.5 h. The solution was centrifuged at 10,000 x*g* for 30 min, then the supernatant was discarded, and the pellet resuspended in Binding Buffer (10 mM HEPES, 150 mM NaCl, 10 mM $MgCl_2$, 1 mM KCl, pH 7.4; 2 mL per 50 mL tube). The resuspended phages were combined to one tube, the phage precipitation was repeated, and phages were ultimately resuspended in 2 mL of Binding Buffer. Any residual bacteria were then pelleted (13,500 x*g*, 20 min) and the supernatant was transferred to a fresh tube. The solutions incubated at 65 °C for 15 min to kill any remaining bacteria before being stored at 4 °C until further use.

Phage Quantification

For all experiments, phages were quantified via a colony forming unit assay. In this assay, serial dilutions of the phage solution were prepared in 2xYT media and 10 µL of each dilution was added to 90 µL of log-phase *E. coli* ER2738. Following addition of the phage dilutions, the culture

was incubated at 37 °C for 45 min and then 10 μL was spotted in triplicate onto agar selection plates containing either 100 μg/mL ampicillin and 10 μg/mL tetracycline, which were incubated at 37 °C overnight. The following day, colonies in each spot were counted and this number was used to calculate the number of colony-forming units in the solution.

Affinity Selection Against *ZNRF3*^{ECD} FXAzF Mutants

Dibenzocyclooctyne (DBCO) coated magnetic beads (100 μL,, 10mg/mL slurry, Kerafast) were transferred to a 1.5 mL tube, washed three times with 1 mL of buffer A, resuspended in 100 uL of buffer A, and split into two tubes. The FXTAG/ZNRF3 mutant was added in one of the tubes, and an equal volume of buffer A was added to the other tube (hereafter referred to as + tube and – tube, respectively). The beads/protein mixture was incubated with shaking for 4 h at 4 °C. The supernatant was then removed and the beads were washed three times with 1ml of Binding Buffer (10 mM HEPES, 150 mM NaCl, 10 mM $MgCl_2$, 1 mM KCl, pH 7.4). 0.25 mL of 5x blocking buffer (Binding Buffer + 5% BSA + 0.5% Tween 20) was added to each tube and phage solution, and they incubated at room temperature with end-over-end rotation for 30 min. The blocking buffer was removed from the – tube, and the purified phage library was incubated with the beads for 30 min at room temperature as a negative selection. The supernatant was then transferred to the resin of the + tube and left to incubate at room temperature for 30 min. After 30 min the supernatant was removed and the resin was washed (5 x 1mL) with Wash Buffer (Binding Buffer + 0.1% Tween 20) to remove nonspecifically bound phages. During each washing step the resin was completely resuspended by inversion. The resin was transferred to fresh tubes after every other wash to remove phages binding to the polypropylene tube. After the last wash, phages were eluted by incubating for 15 min with 100 μL of Elution Buffer (50 mM glycine, pH 2.2). The supernatant was then removed and immediately added to 50 μL of Neutralization Buffer (1 M Tris, pH 8.0). The neutralized elution was immediately used for amplification.

Phage Amplification: A small aliquot (10 μL) of the phage elution was removed for quantification of phages. The remaining solution was added to an actively growing culture of *E. coli* ER2738 (OD_{600} = 0.5-0.6) in 20 mL 2xYT containing 10 μg/mL tetracycline for 45 min at 37 °C with rotation. After 45 min the cells were pelleted (3750 x *g*, 15 min), resuspended in 200 mL of 2xYT containing 100 μg/mL ampicillin and 10 μg/mL tetracycline, and amplified overnight at 37 °C. The following day, phagemids were extracted from the amplified culture using a

commercial plasmid purification kit. The remaining culture was then inoculated into 100 mL of fresh 2xYT containing 100 µg/mL ampicillin and 10 µg/mL tetracycline for subsequent phage expression.

NHS Control Selection: NHS-functionalized magnetic beads (100 µL, Cytiva, NHS Mag Sepharose) were washed with 1 mL of Equilibration Buffer (1 mM HCl, cold) then separated into two tubes (+/-). Following buffer exchange into Binding Buffer (10 mM HEPES, 150 mM NaCl, 10 mM $MgCl_2$, 1 mM KCl, pH 7.4), 26 µg of WT ZNRF3 ECD was added to the positive tube and incubated for 15 min at room temperature. The supernatant was then removed and the residual active groups were blocked by six alternative washes of buffer 1 (0.5 M ethanolamine, 0.5 M NaCl, pH 8.3) and buffer 2 (0.1 M NaAcO, 0.5 M NaCl, pH 4.0). During the second wash with buffer 1, the beads were incubated for 15 min at room temperature. Following blocking with buffers 1 and 2, the beads and phages were then incubated with 1x Blocking Buffer (Binding Buffer + 0.1% Tween 20 + 1% BSA) for 30 min at room temperature. Phages were added to the negative tube and incubated for 30 min with shaking. The supernatant was removed, then added to the positive tube for a 30 min incubation with shaking. The bound phages were then washed with Wash Buffer (5 x 1 mL), and transferred into a fresh tube after every other wash. Bound phages were then eluted for 15 min in 100 µL of Elution Buffer (0.1 M glycine, pH 2.2) and the supernatant was neutralized with 50 µL of 1 M Tris, pH 8.0. Eluted phages were then directly used for amplification.

Illumina Sequencing of Selected Phage Libraries

A four-step PCR cycle was used to amplify the library region out of the original phagemid library using primers NGS-F1 and NGS-R1, as has been previously described.[36] The amplicons were purified and extracted from a 3% agarose gel according to a GenCatch gel extraction kit, then indices were attached using a subsequent PCR with NGS-i7 and NGS-i5 primers. To identify the rounds of selection, each round contained a unique combination of i7 or i5 indices. The PCR products were purified using a GenCatch gel extraction kit and submitted to the Genomics and Bioinformatics Center at Texas A&M University for sequencing on an Illumina iSeq (4M Reads, 2x150bp). Sequences underwent paired-end filtering and were analyzed for enrichment in R. All scripts are available in the Supporting Information.

Biolayer Interferometry Assays

Biolayer interferometry (BLI) experiments were performed using streptavidin-coated biosensors (Sartorius Bio) on an Octet R8 biolayer interferometer (Sartorius Bio). All experiments were performed in assay buffer (10 mM HEPES, 150 mM NaCl, 0.01% Tween 20, pH 7.5). Biotinylated ZNRF3 and/ or Biotinylated mutants (28 µg/mL in assay buffer) were loaded onto the sensor to at least 2 but no greater than 5 nm of loading over 10 min, then the sensors were quenched for one minute with a 1% BSA solution in assay buffer. This loading range was used to minimize variation in immobilized protein density between experiments. For RSPO-2 kinetic characterization with wildtype and mutant proteins, RSPO-2 was serially diluted in assay buffer, and the association and dissociation to immobilized proteins were measured over 5 min each. Peptides were serially diluted in assay buffer containing 0.8% DMF and the association and dissociation to ZNRF3 were measured over 3.5 and 5 min, respectively. Between each experiment, sensors were regenerated using 10 mM glycine, pH 1.5. Binding curves were referenced against a buffer-only blank. All curves were fit in the Octet Kinetic Analysis program (Sartorius Bio) using a 1:1 protein: ligand binding model, and affinities (K_D) were obtained from global kinetic fits of multi-concentration sensorgrams.

Peptide Synthesis

Automated Peptide Synthesis

Initial sequences of peptides were synthesized on a low loading ProTide rink amide resin (CEM #R002) using a MultiPep 2 Peptide Synthesizer (CEM). All amino acid derivatives were standard derivatives for Fmoc peptide synthesis commercially purchased from Chem-Impex. Fmoc-amino acids were deprotected using 20% piperidine in DMF (2 x 2 mL, 5 min at 50 °C). Amino acids were coupled using HATU/NMM double coupling cycles (4.2 eq amino acid, 4.2 eq HATU, 8 eq NMM, 15 min at 50 °C). Following synthesis, the resin was dried with ethanol washes (3 x 3 mL) followed by dichloromethane washes (3 x 3 mL) and evaporated under vacuum.

Peptide Cleavage and Oxidation of Disulfide-Cyclized Peptides

Peptides were cleaved in 2 mL of 92.5:2.5.2.5:2.5 TFA:TIS:DODT:H_2O for 3 hours at room temperature. Following cleavage, the resin was filtered, and peptides were precipitated from the filtrate by an 8-fold dilution in cold diethyl ether. After collection by centrifugation (5251 x*g*, 15 min), the pellet was dried under nitrogen gas. Linear peptides were oxidized by dissolving them

in 1 mL of 1:2:2 DMSO:ACN:H_2O and incubated for 24 h at 37°C with gentle shaking. The mixture was then diluted in water and lyophilized before purification with HPLC-MS. Refer to the conditions in Table S2 for the purification conditions of each peptide.

SUPPLEMENTARY FIGURES

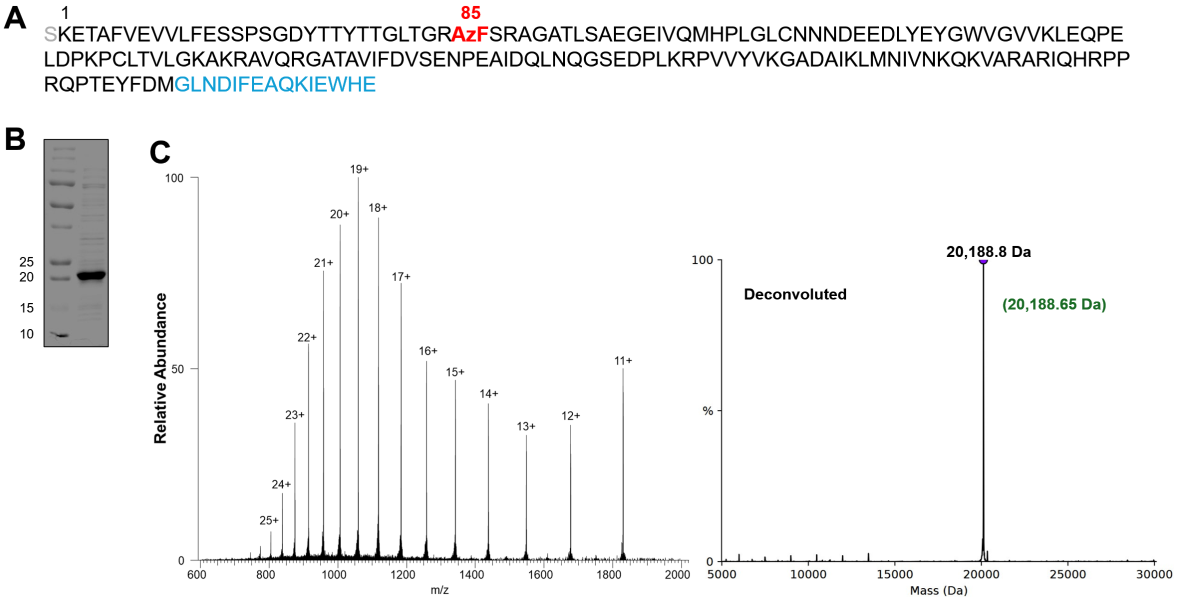

Fig. S1 Characterization of F85AzF/ZNRF3 extracellular domain. A) Protein sequence for the mutant fused to the AviTag (blue) with the indicated mutation. B) SDS-PAGE analysis of the purified sample. Molecular weight standards are labelled KDa. C) ESI-MS spectra and deconvoluted mass. The value in green indicates the theoretical mass of the protein.

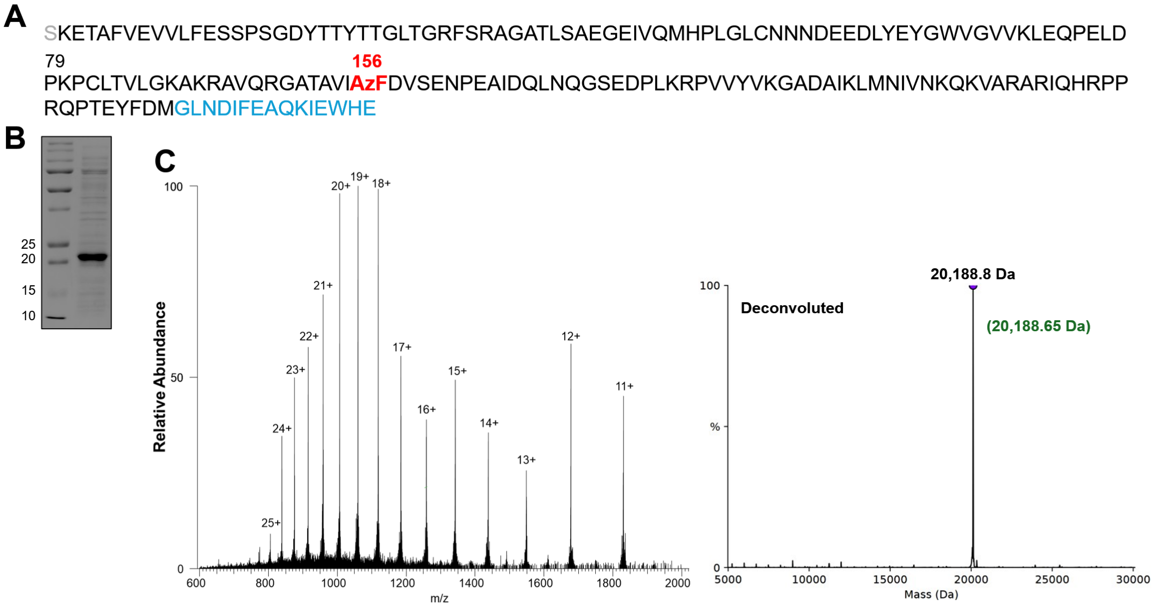

Fig. S2 Characterization of F156AzF/ZNRF3 extracellular domain. A) Protein sequence for the mutant fused to the AviTag (blue) with the indicated mutation. B) SDS-PAGE analysis of the purified sample. Molecular weight standards are labelled KDa. C) ESI-MS spectra and deconvoluted mass. The value in green indicates the theoretical mass of the protein.

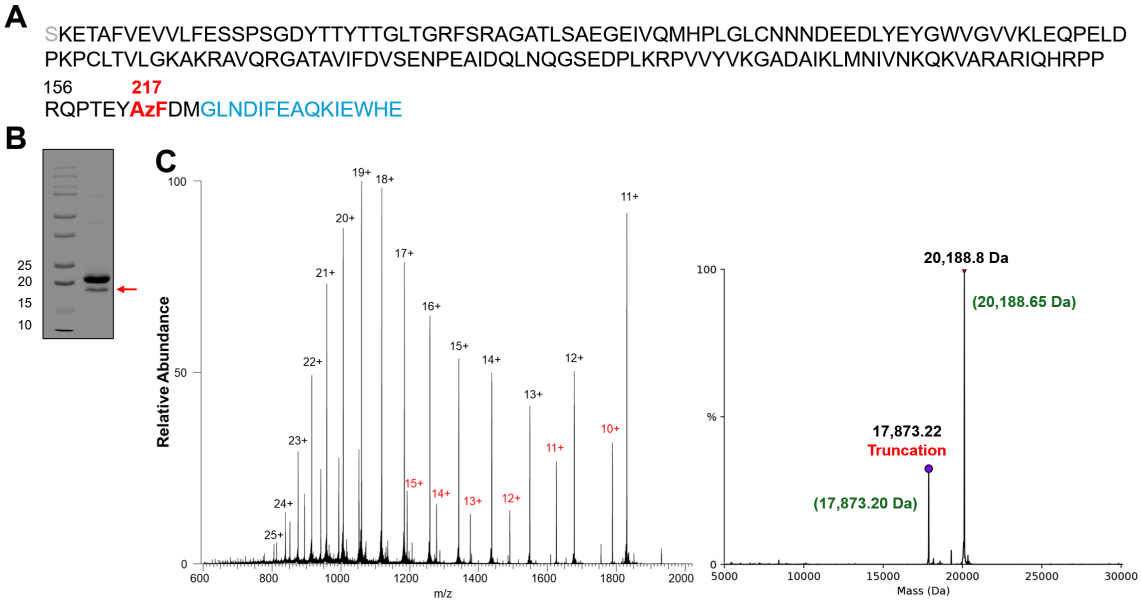

Fig. S3 Characterization of F217AzF/ZNRF3 extracellular domain. A) Protein sequence for the mutant fused to the AviTag (blue) with the indicated mutation (red). B) SDS-PAGE analysis of the purified sample. The red arrow indicates the truncation that was a result of failed AzF incorporation via amber suppression. Molecular weight standards are labelled in KDa. C) ESI-MS spectra and deconvoluted mass. The green

value indicates the theoretical mass of the protein. Charge states in black are for the full-length protein; red indicates truncation charge states.

A

1
SKETAFVEVVLFESSPSGDYTTYTTGLTGR**F**SRAGATLSAEGEIVQMHPLGLCNNNDEEDLYEYGWVGVVKLEQPELD
PKPCLTVLGKAKRAVQRGATAVI**F**DVSENPEAIDQLNQGSEDPLKRPVVYVKGADAIKLMNIVNKQKVARARIQHRPPR
QPTEY**F**DMGLNDIFEAQKIEWHE

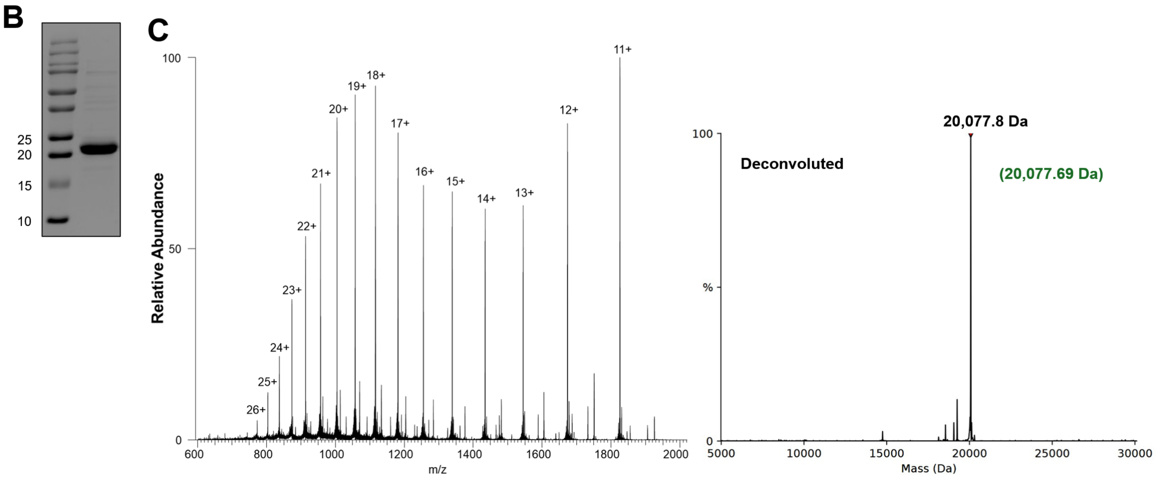

Fig. S4 Characterization of WT ZNRF3 extracellular domain. A) Protein sequence for the protein fused to the AviTag (blue) with the subsequent phenylalanine mutation sites indicated in bold. B) SDS-PAGE analysis of the purified sample. Molecular weight standards are labelled in KDa. C) ESI-MS spectra and deconvoluted mass. The green value indicates the theoretical mass of the protein.

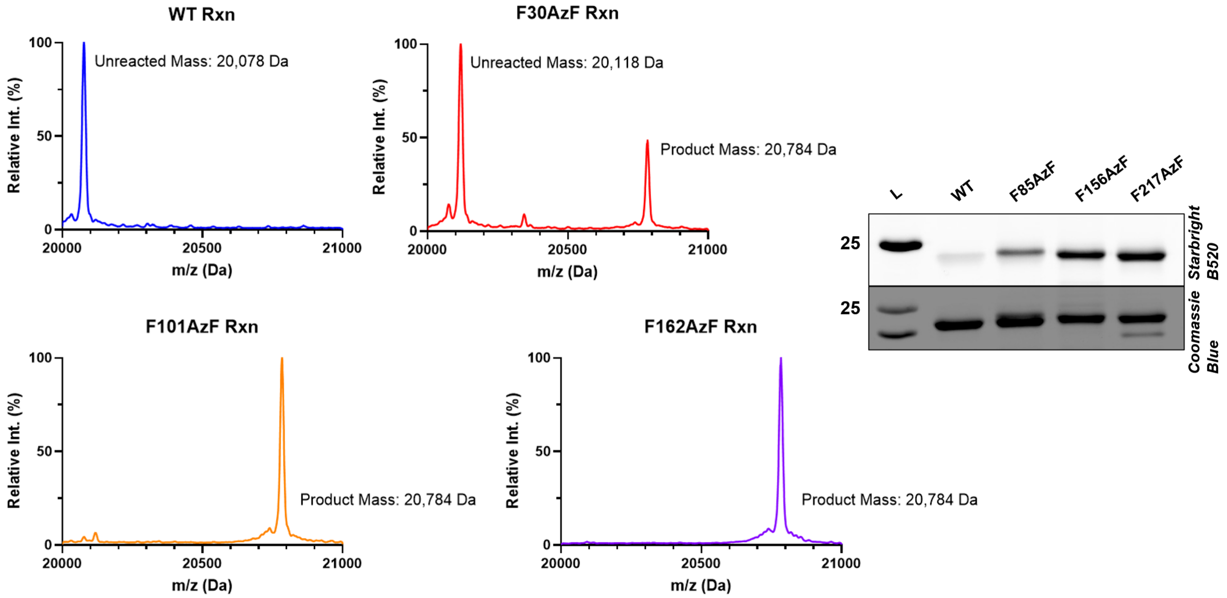

Fig. S5. Wild type and AzF Mutant SPAAC reaction with DBCO-Fluorescein Characterization via HRMS and Fluorescent & Coomassie Blue SDS-PAGE Analysis

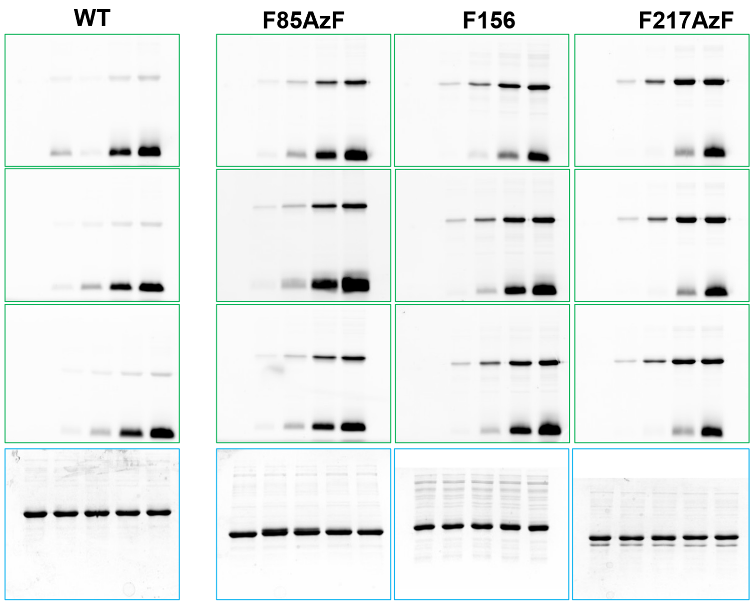

Fig. S6. Fluorescent and Coomassie Triplicate Gels used for the quantification of SPAAC fluorescein labeling reactions in Image J software. Green border: Fluorescent Gel (Starbright B525 imaging), Blue Border: Coomassie Blue Staining

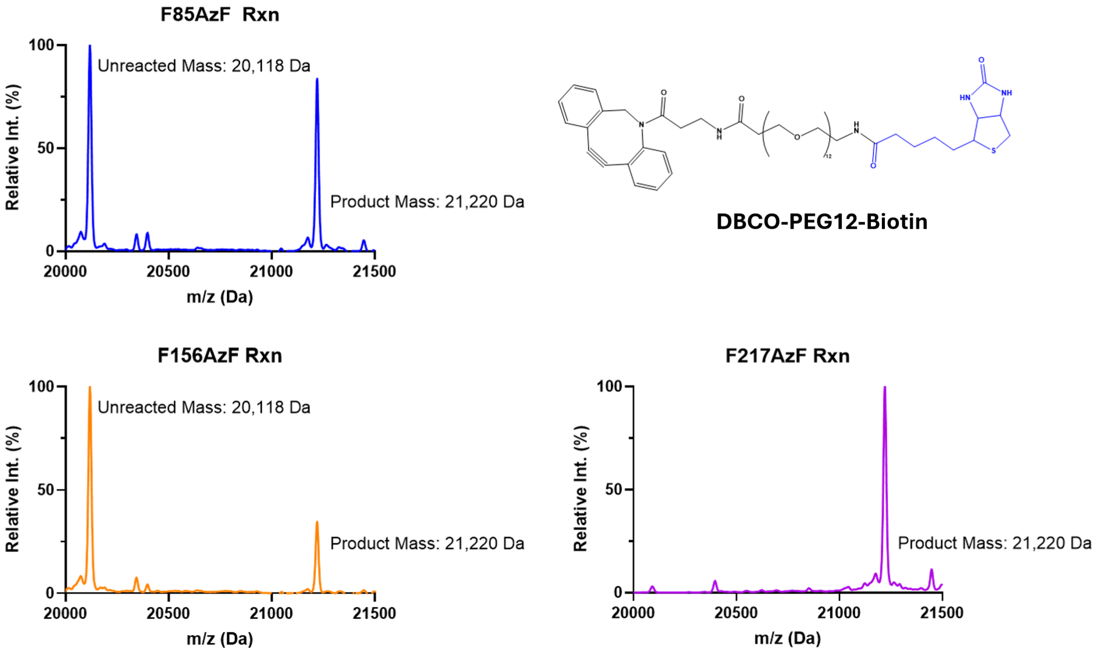

Fig. S7. AzF Mutant SPAAC reaction with DBCO-PEG12-Biotin for Rspondin-2 affinity probing HRMS Characterization

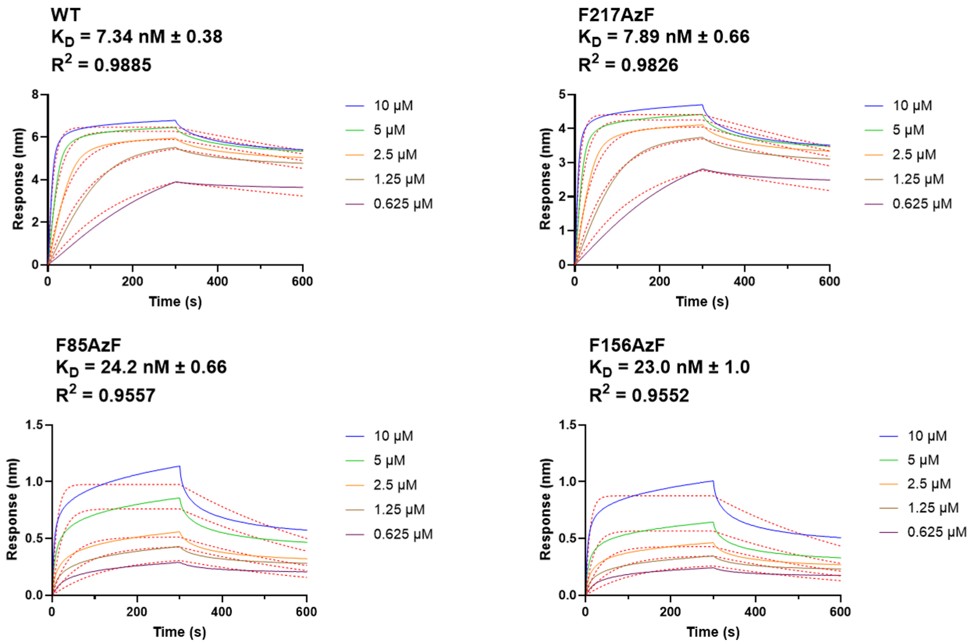

Fig. S8. Dose-Response Curves of Rspondin 2 Binding to Mutant Proteins Compared to Wildtype. KD values are reported as the mean ± s.d. of three independent experiments (n = 3). Raw traces (multicolored by concentration) were fitted (red dotted lines) to a 1:1 protein/ligand binding model to calculate kinetic parameters.

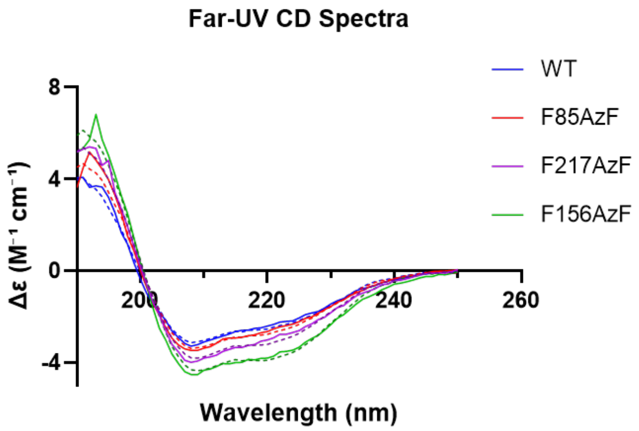

Fig. S9. Overlaid Far-UV CD Spectra of WT and AzF Mutant ZNRF3 Ectodomain Proteins. CD spectra (Δε, M^{-1} cm^{-1}) were collected from 250–190 nm at 25 °C in 10 mM phosphate, 50 mM NaF (pH 7.5). Spectra are shown as the mean of four technical replicate scans (n = 4) following buffer baseline subtraction. Raw traces (solid lines; color-coded by protein) were fit (dashed lines; color-coded by construct) using secondary-structure deconvolution (BeStSel)

Comparison of Round 3 Sequences

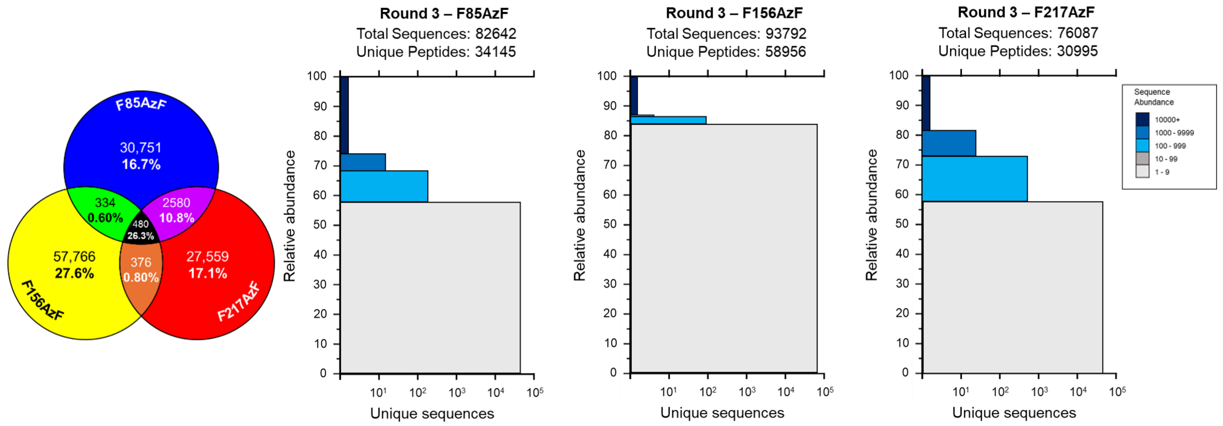

Fig. S10. Comparative Round 3 Next-generation Sequencing Enrichment Analysis.

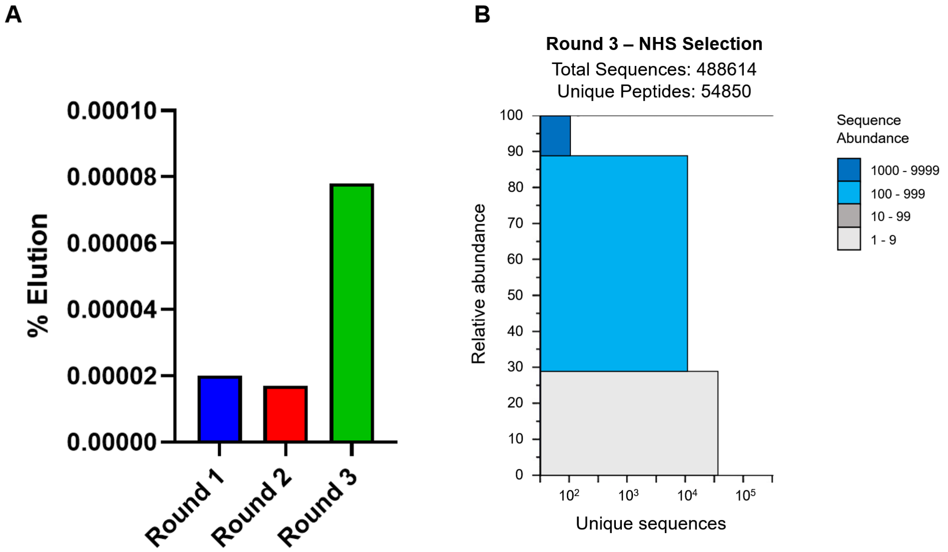

Figure S11. NHS-based selection against wild-type ZNRF3. (A) Phage recovery (% elution) over three rounds of selection with wild-type ZNRF3 ECD immobilized on NHS-activated magnetic beads. (B) Round 3 enrichment profile showing 488,614 total sequences and 54,850 unique peptides, with most peptides present at low or intermediate copy number, indicative of a diffuse enrichment outcome.

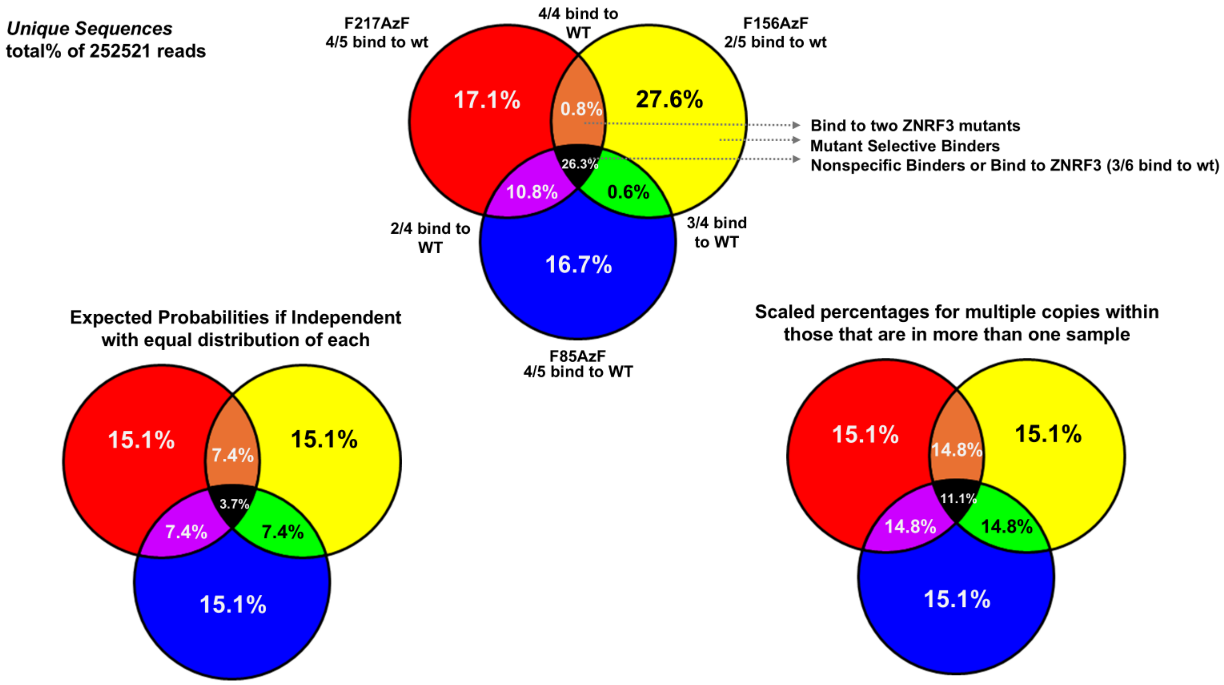

Fig. S12. Venn Diagram Grouping of Round 3 Sequences

A

Sequence	N	% Abundance	K_D μM
**F85AzF Top 5**			
CPSWVKAHYGPAAC	21813	26.4	NB
CKTHWIWVKWRFVC	995	1.2	0.124
CPMWLERHFGPEAC	662	0.80	NB
CMFPDNLAFVHFFC	459	0.55	NB
CFPWWDGHTWHLFC	311	0.38	0.500
**F156AzF Top 5**			
CPSWVKAHYGPAAC	12440	13.3	NB
CDLKYYNVYGIEMC	475	0.506	NB
CHWPSSSYAYQWWC	173	0.184	NB
CWFTTDTHGVYYIC	114	0.122	NB
CHQSSWSMWSKWMC	84	0.0896	8.75
**F217AzF Top 5**			
CPSWVKAHYGPAAC	13871	18.2	NB
CLYHYQWKWWPRYC	765	1.01	1.67
CKTHWIWVKWRFVC	551	0.724	0.124
CMFPDNLAFVHFFC	421	0.553	NB
CNMWPWWSYSYRVC	284	0.373	5.96

B

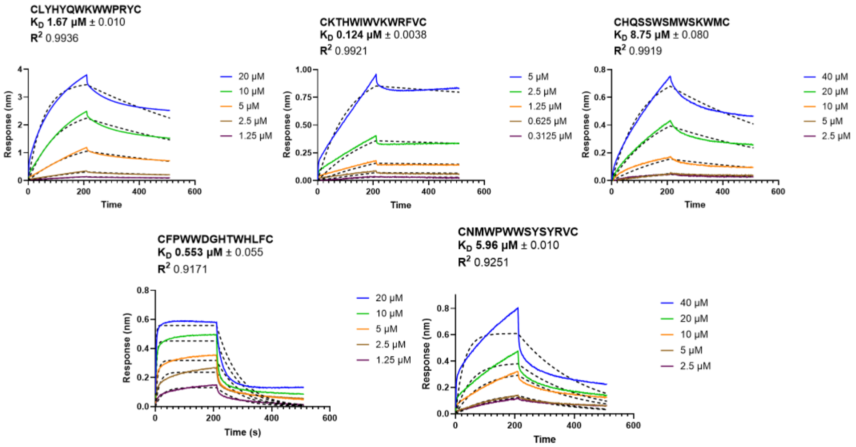

Fig. S13. Characterization of mutant-selected macrocyclic peptides for binding to wild-type ZNRF3 ectodomain. (A) Top five peptide sequences from each mutant group identified by next-generation sequencing were synthesized by solid-phase peptide synthesis, purified by HPLC, and ranked by relative abundance. (B) Biolayer interferometry analysis of representative peptides revealed measurable binding interactions with wild-type ZNRF3.

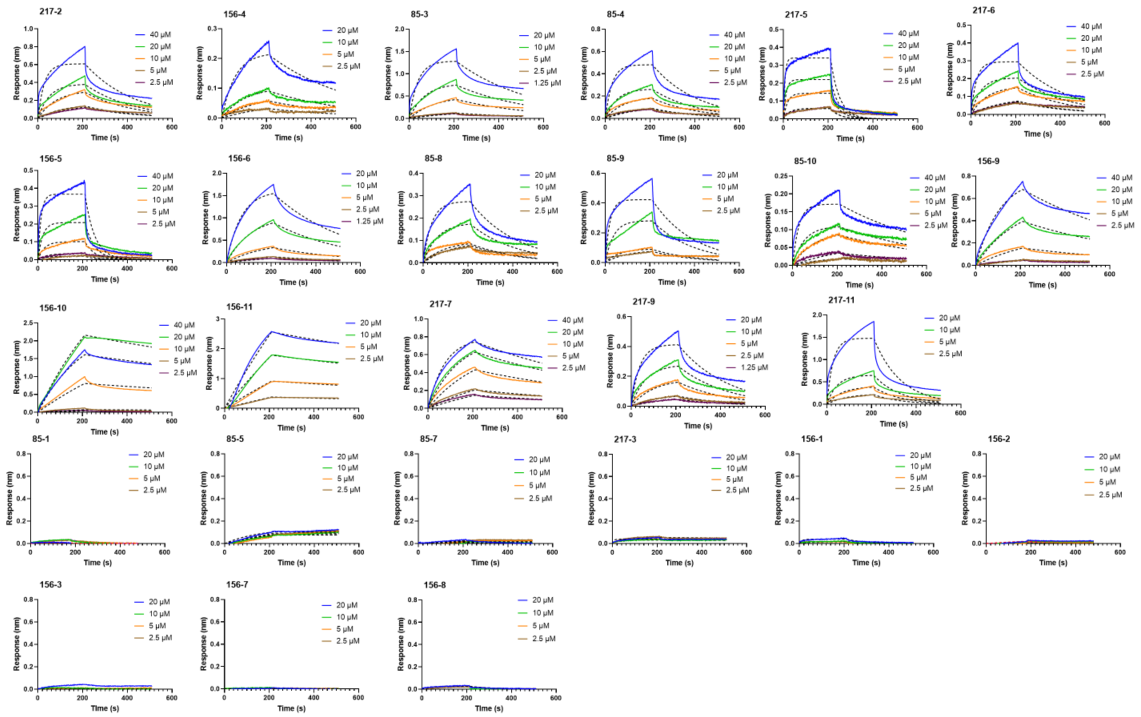

Fig. S14. BLI Traces of Surveyed Peptides: Binders and Nonbinders.

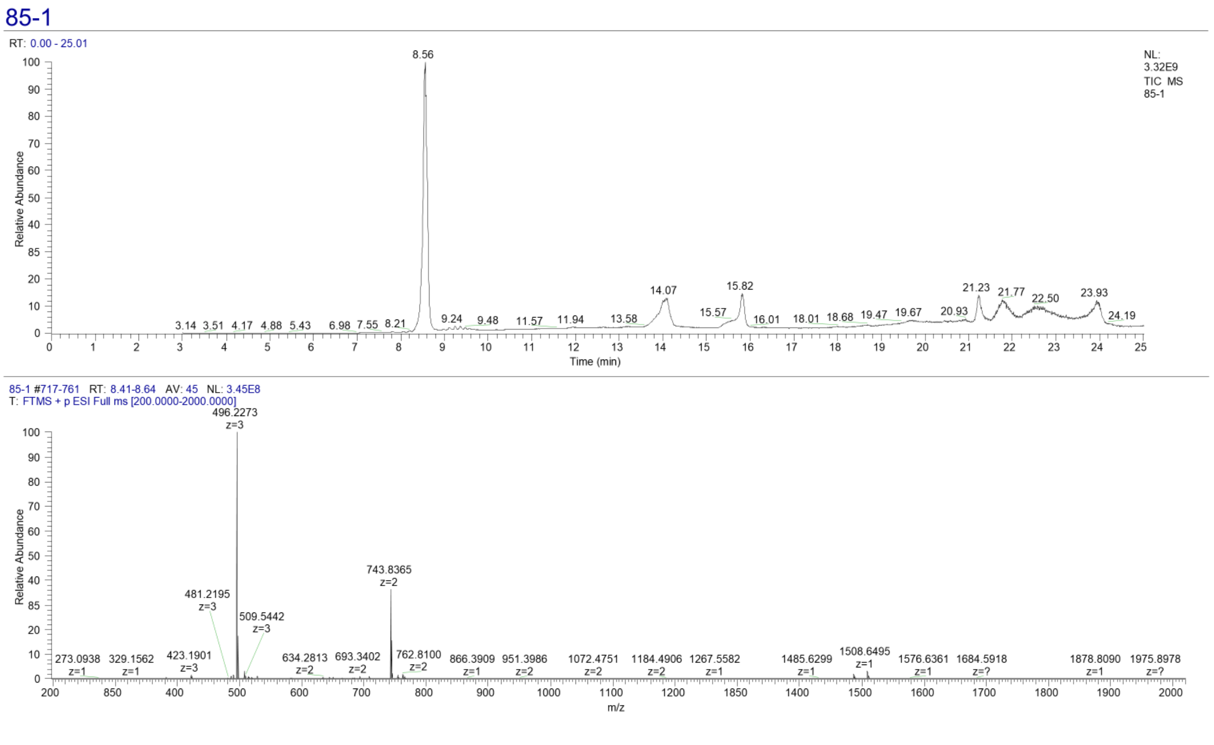

Figure S15: LC-MS Data for 85-1. The TIC chromatogram is shown on top and the extracted masses from the peak are below.

85-2

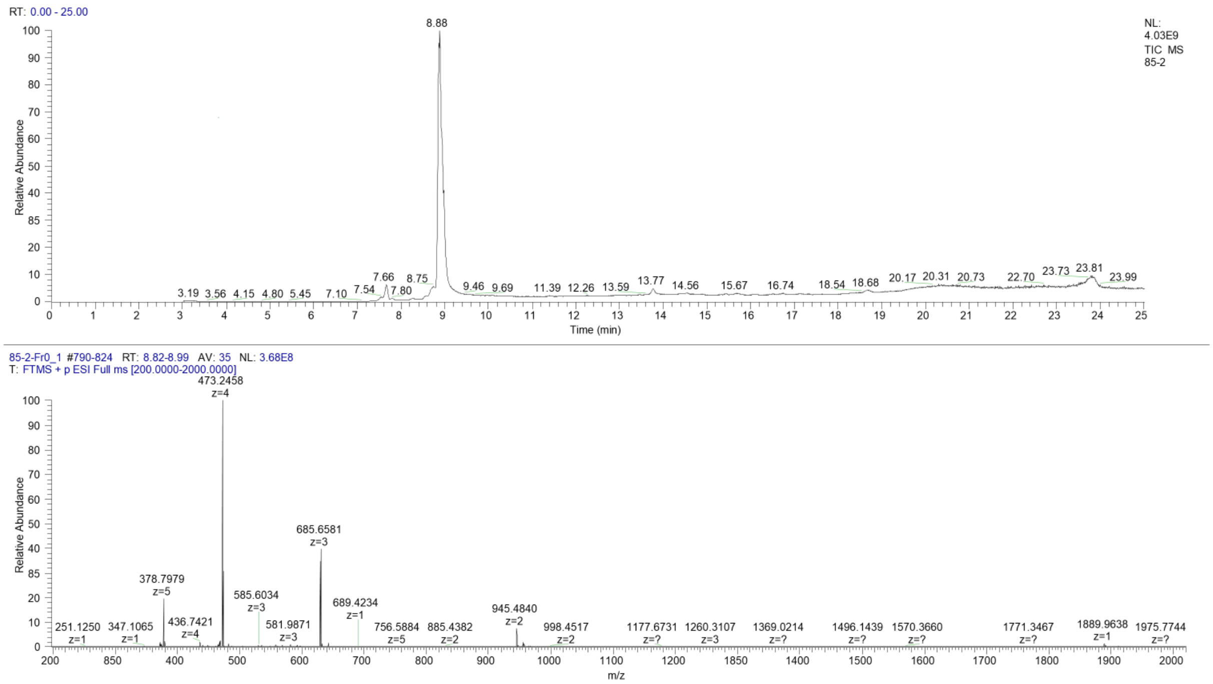

Figure S16: LC-MS Data for 85-2. The TIC chromatogram is shown on top and the extracted masses from the peak are below.

85-3

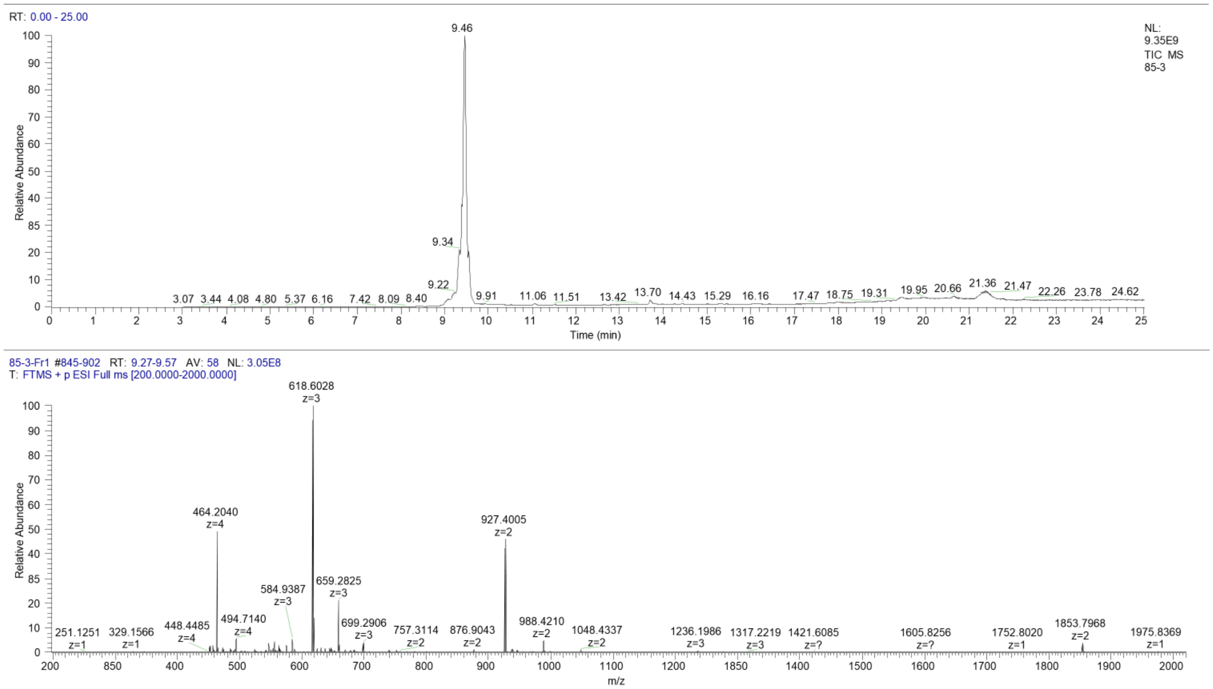

Figure S17: LC-MS Data for 85-3. The TIC chromatogram is shown on top and the extracted masses from the peak are below.

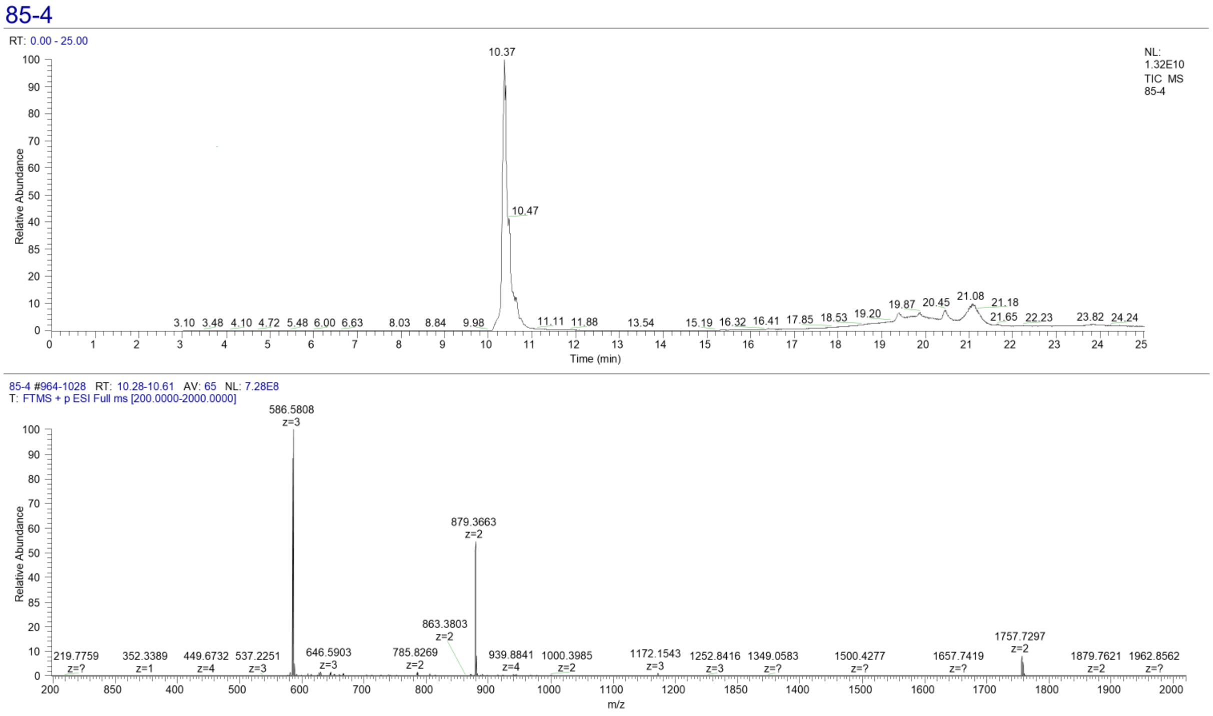

Figure S18: LC-MS Data for 85-4. The TIC chromatogram is shown on top and the extracted masses from the peak are below.

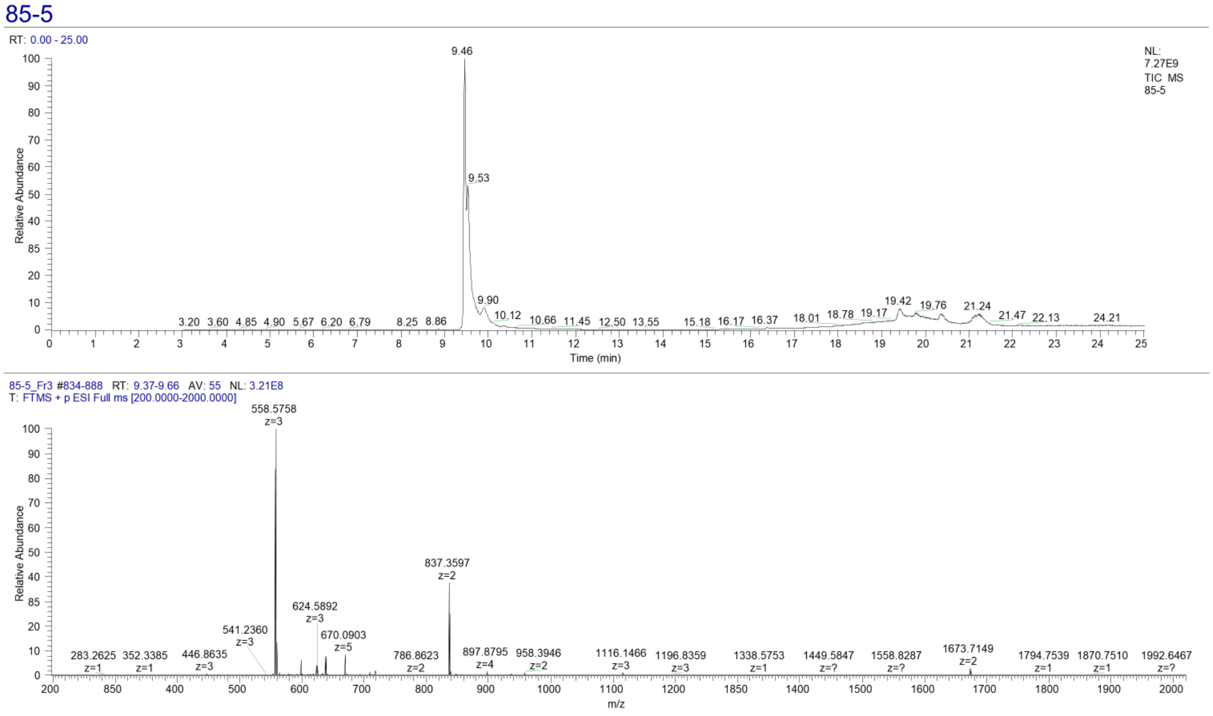

Figure S19: LC-MS Data for 85-5. The TIC chromatogram is shown on top and the extracted masses from the peak are below.

85-6

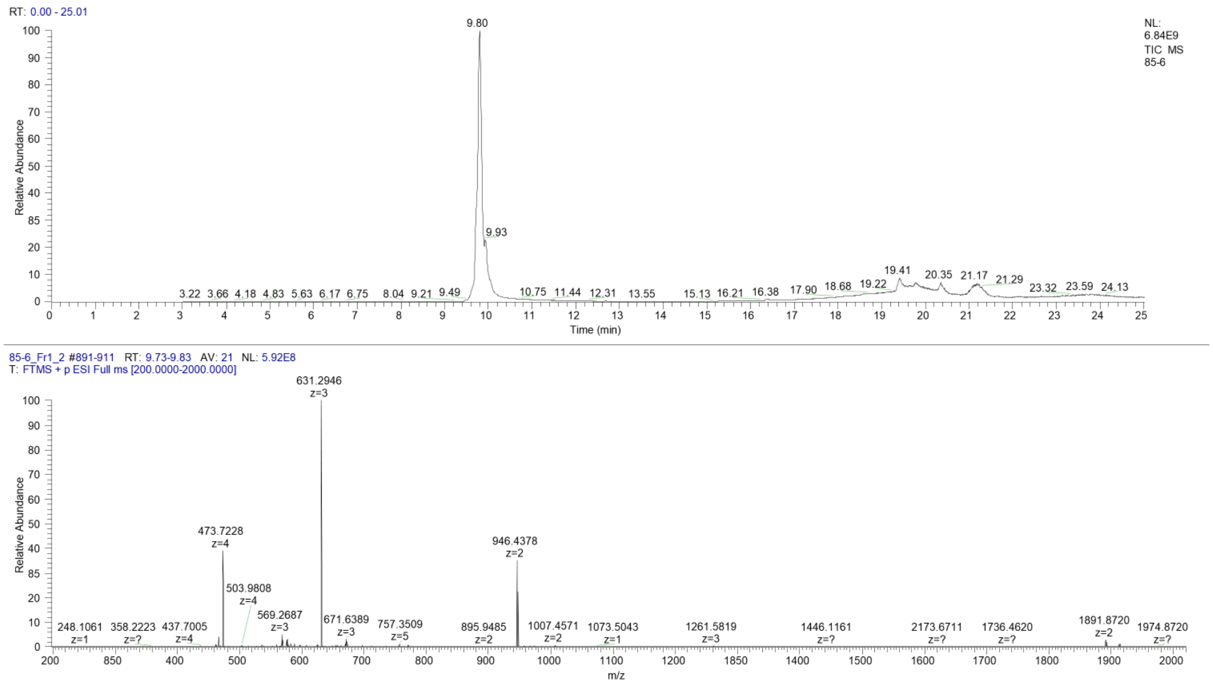

Figure S20: LC-MS Data for 85-6. The TIC chromatogram is shown on top and the extracted masses from the peak are below.

85-7

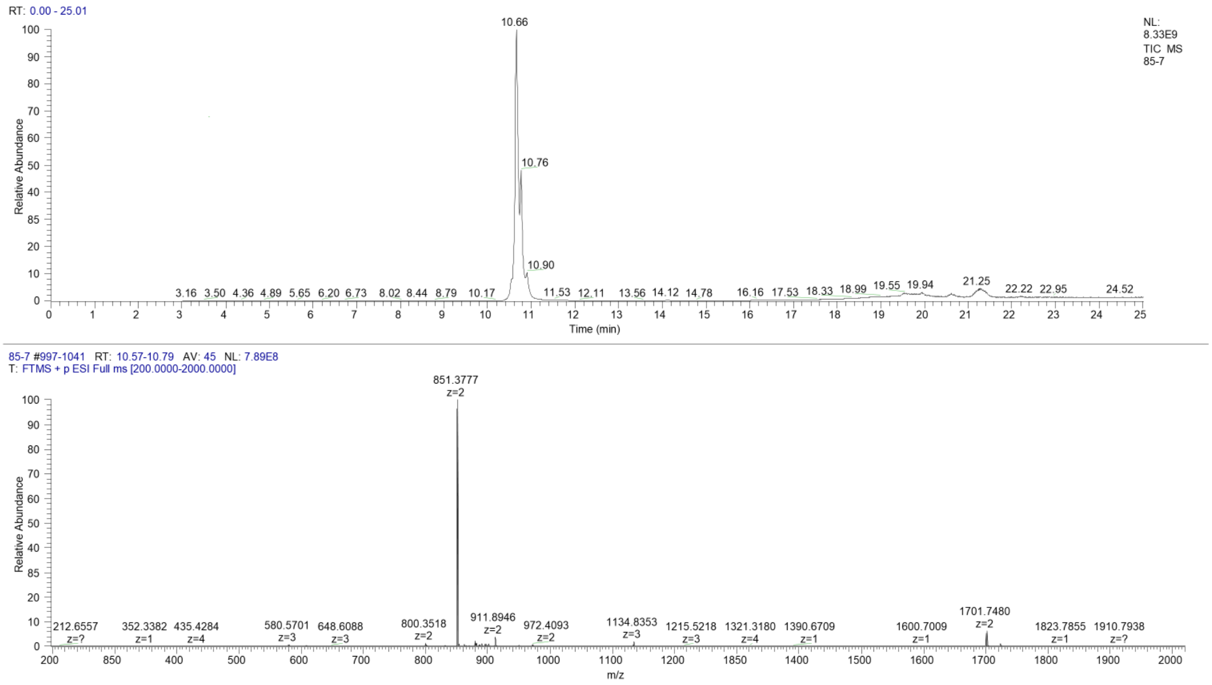

Figure S21: LC-MS Data for 85-7. The TIC chromatogram is shown on top and the extracted masses from the peak are below.

85-8

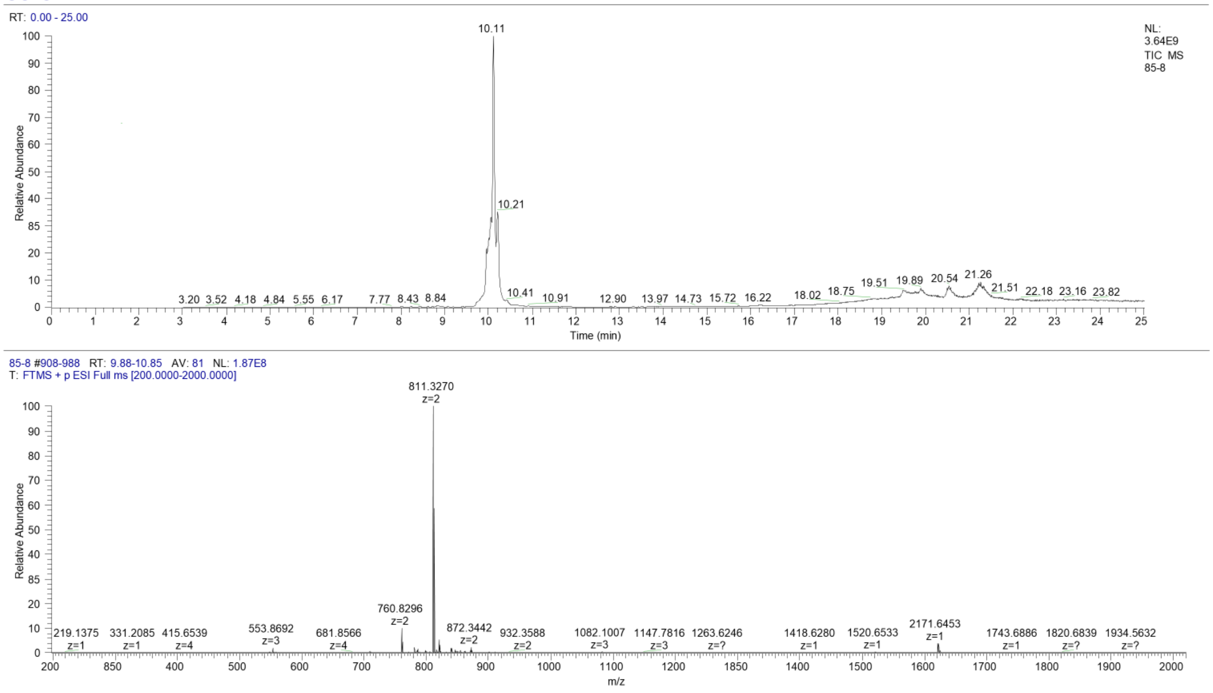

Figure S22: LC-MS Data for 85-8. The TIC chromatogram is shown on top and the extracted masses from the peak are below.

85-9

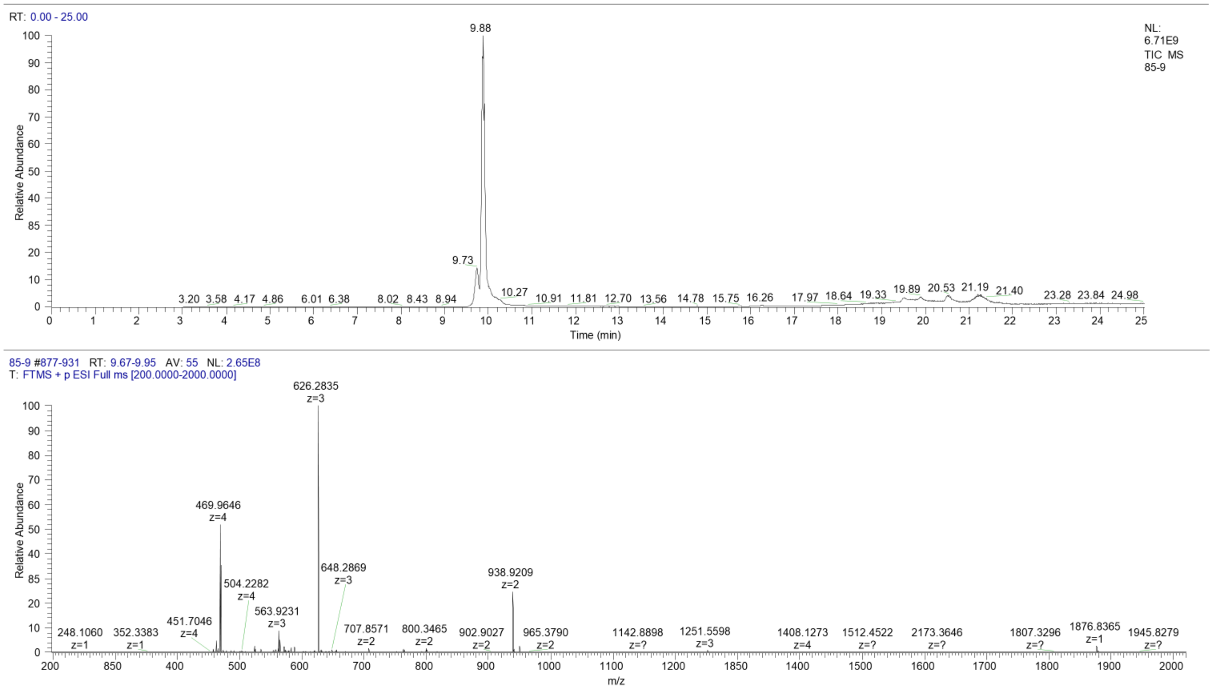

Figure S23: LC-MS Data for 85-9. The TIC chromatogram is shown on top and the extracted masses from the peak are below.

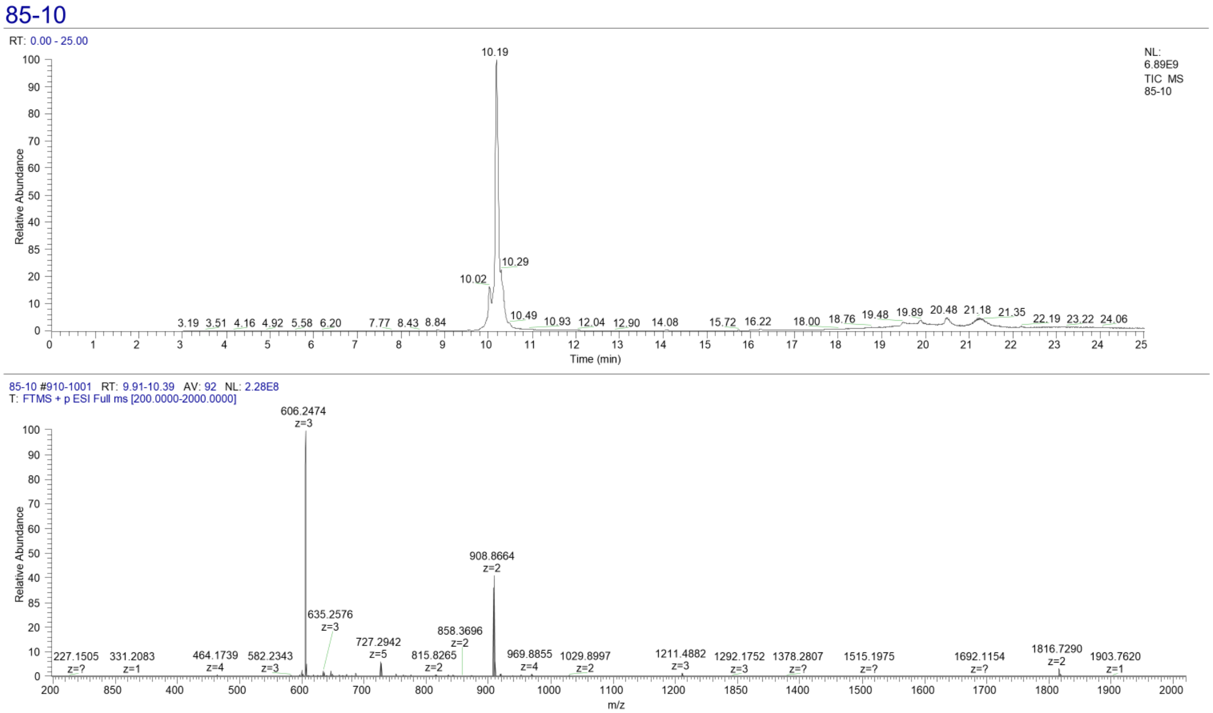

Figure S24: LC-MS Data for 85-10. The TIC chromatogram is shown on top and the extracted masses from the peak are below.

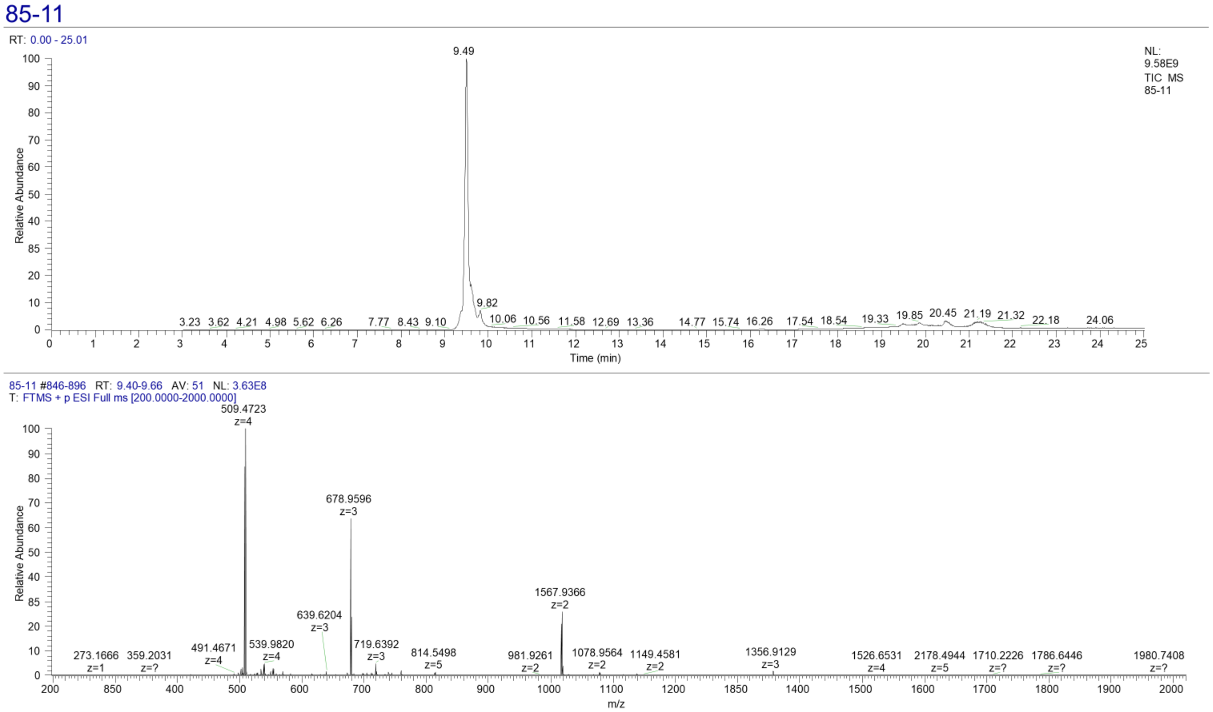

Figure S25: LC-MS Data for 85-11. The TIC chromatogram is shown on top and the extracted masses from the peak are below.

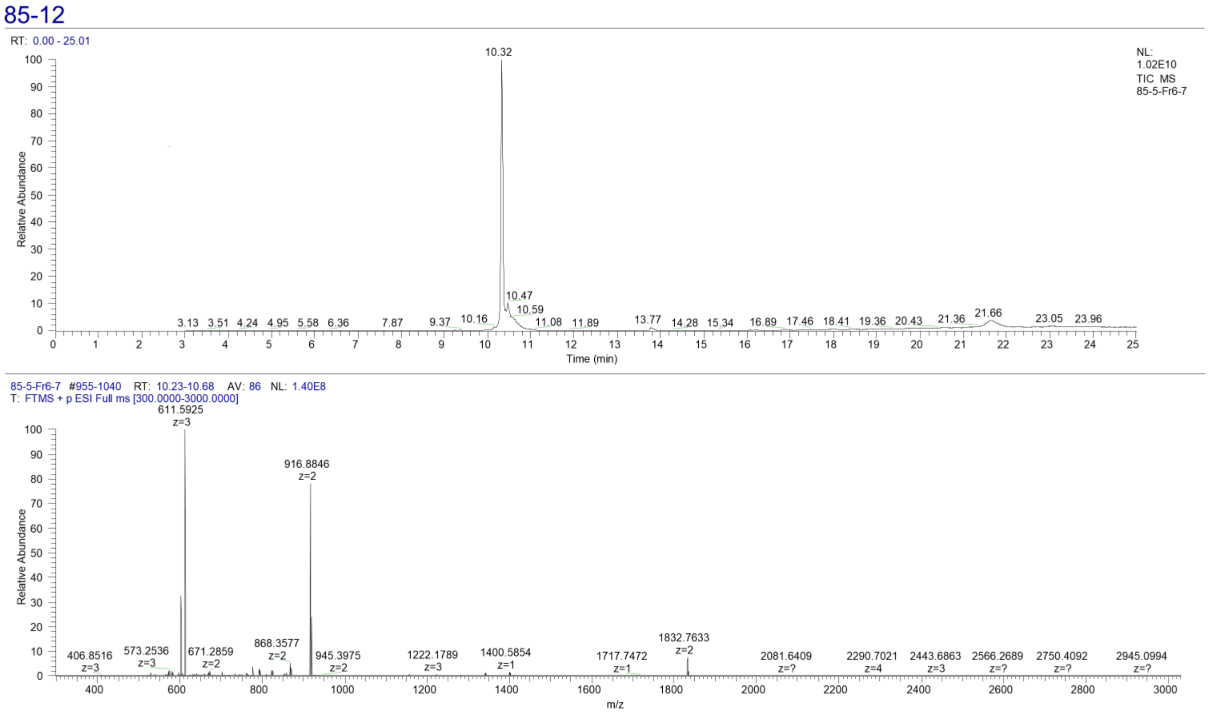

Figure S26: LC-MS Data for 85-12. The TIC chromatogram is shown on top and the extracted masses from the peak are below.

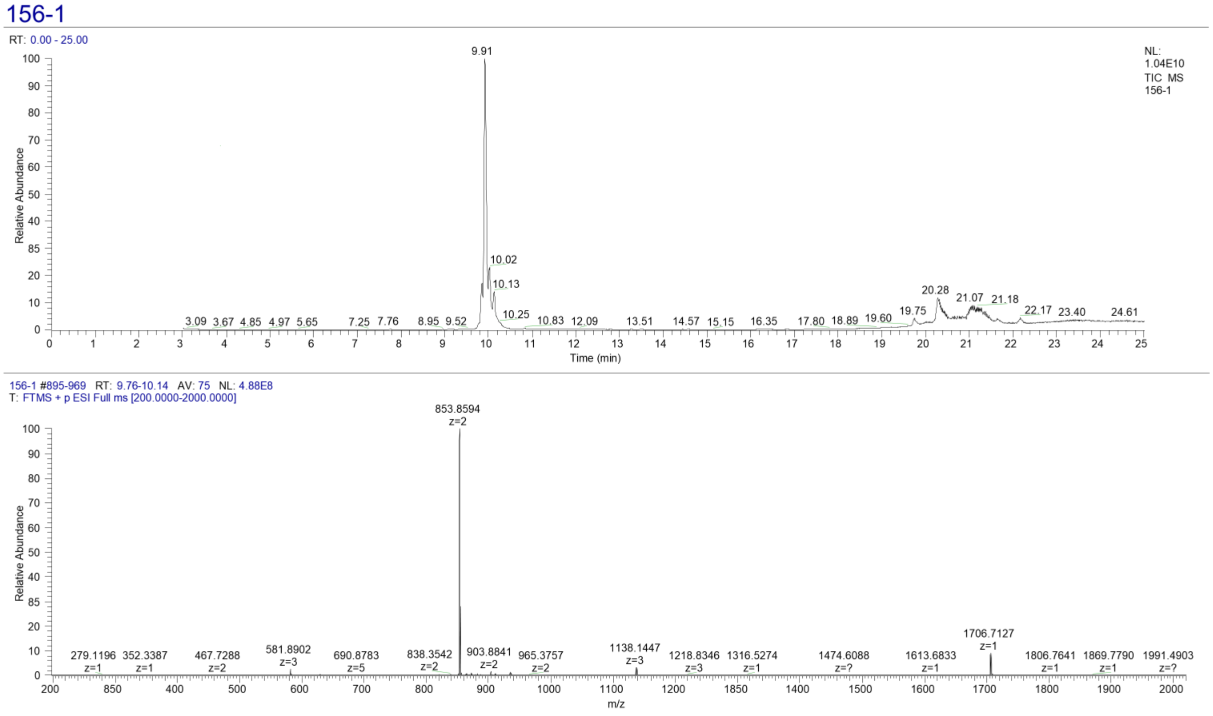

Figure S27: LC-MS Data for 156-1. The TIC chromatogram is shown on top and the extracted masses from the peak are below.

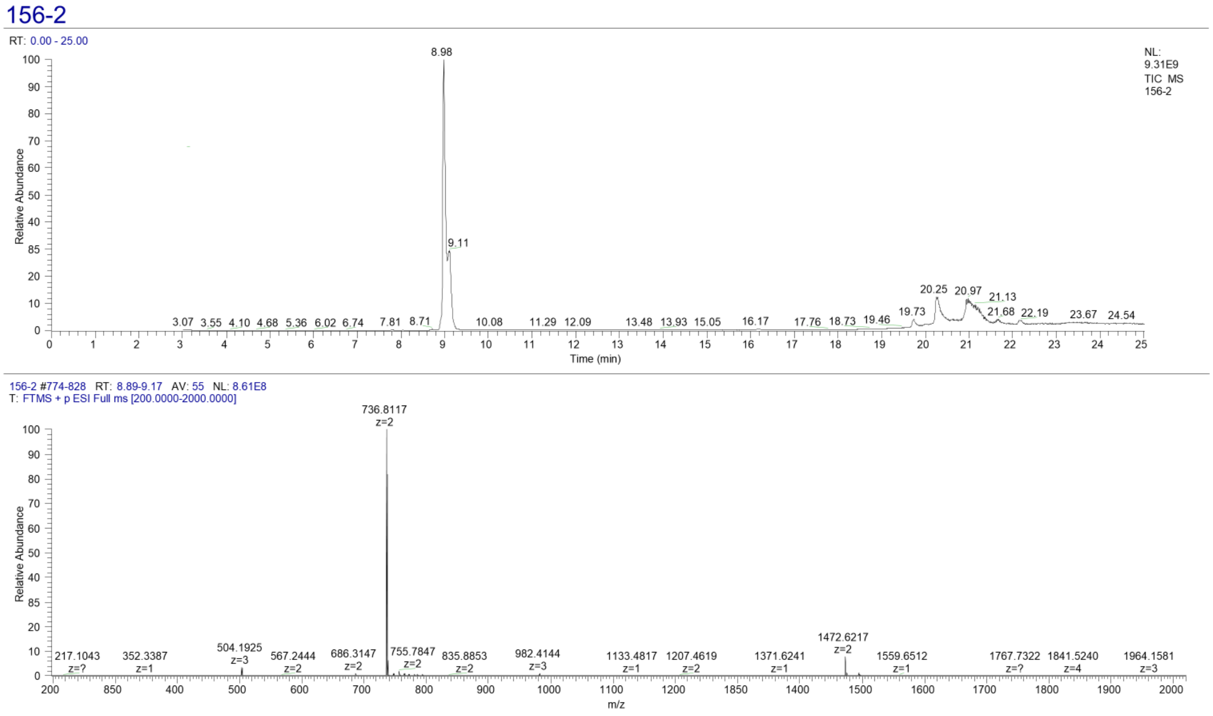

Figure S28: LC-MS Data for 156-2. The TIC chromatogram is shown on top and the extracted masses from the peak are below.

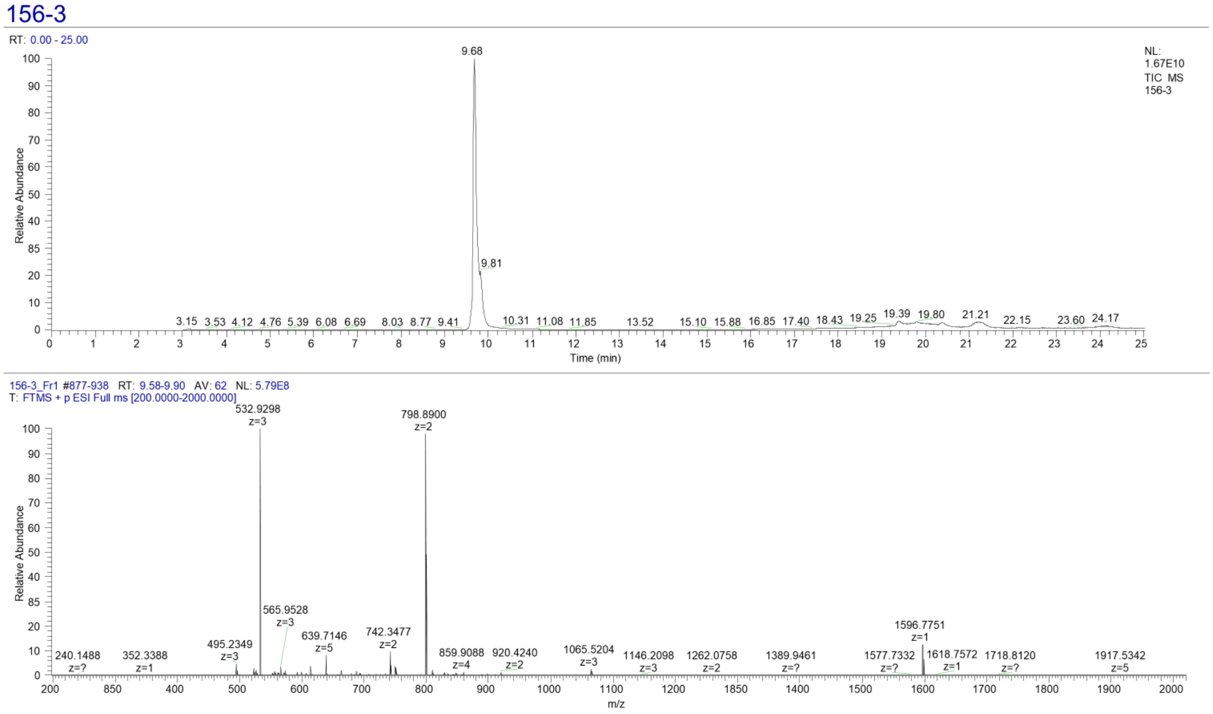

Figure S29: LC-MS Data for 156-3. The TIC chromatogram is shown on top and the extracted masses from the peak are below.

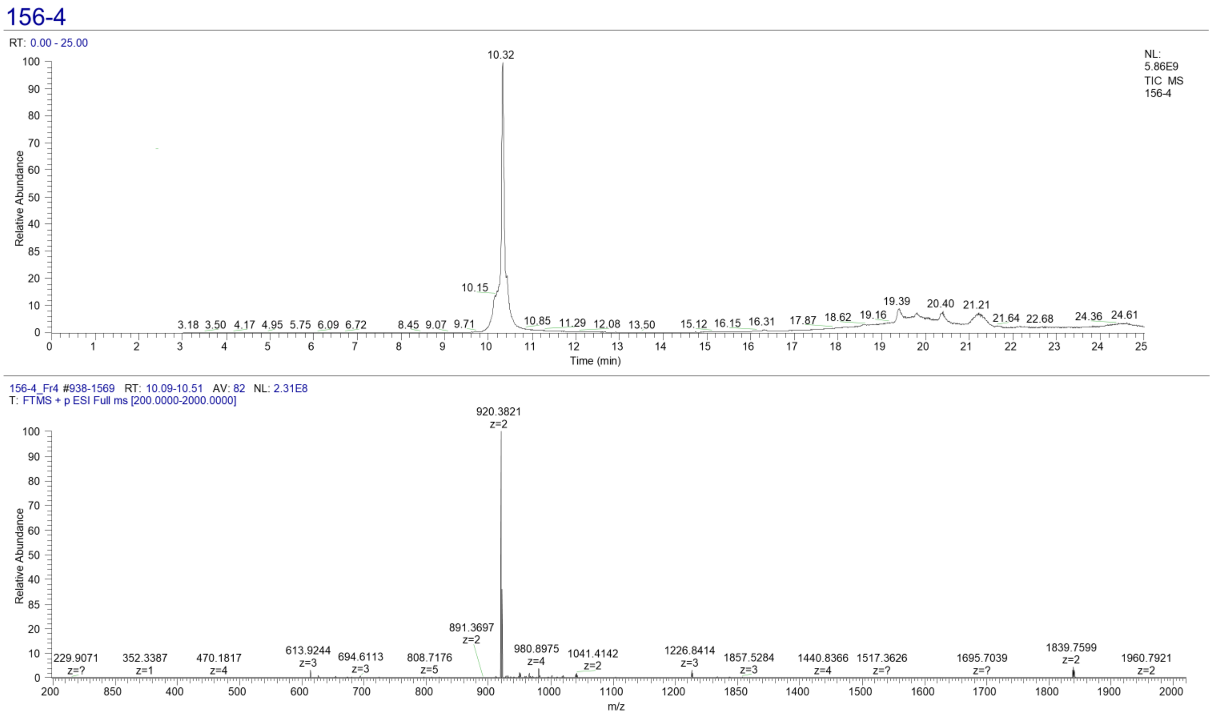

Figure S30: LC-MS Data for 156-4. The TIC chromatogram is shown on top and the extracted masses from the peak are below.

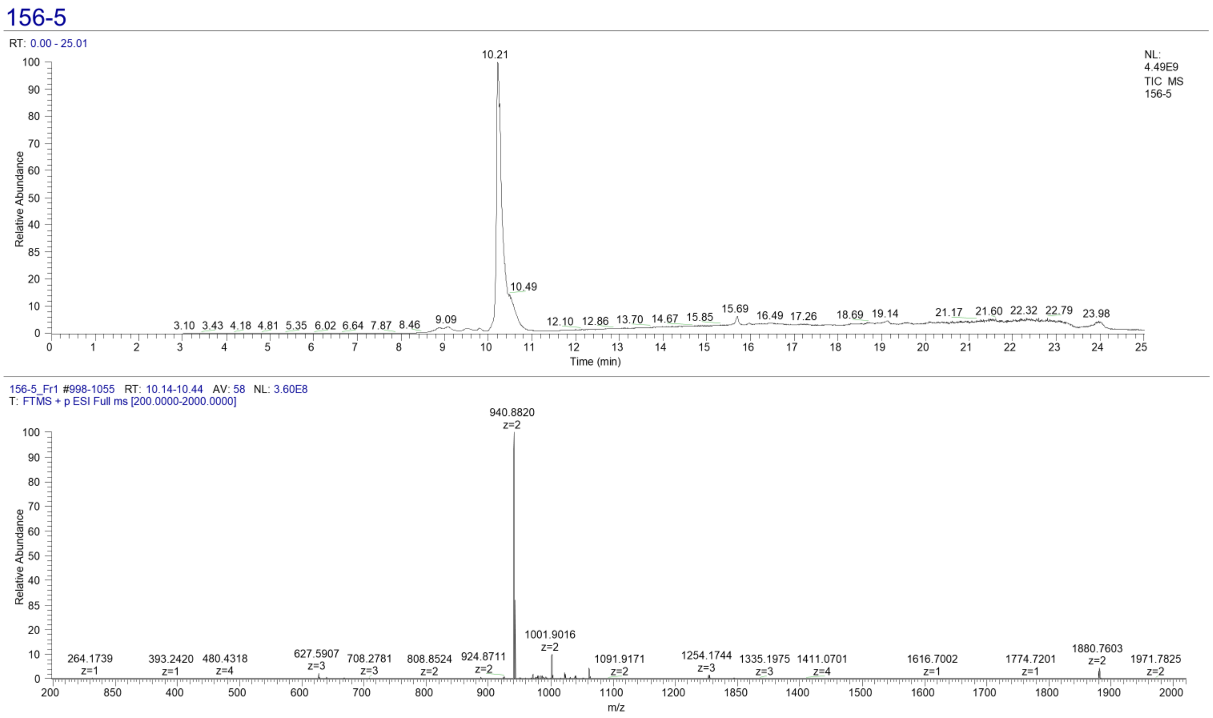

Figure S31: LC-MS Data for 156-5. The TIC chromatogram is shown on top and the extracted masses from the peak are below.

156-6

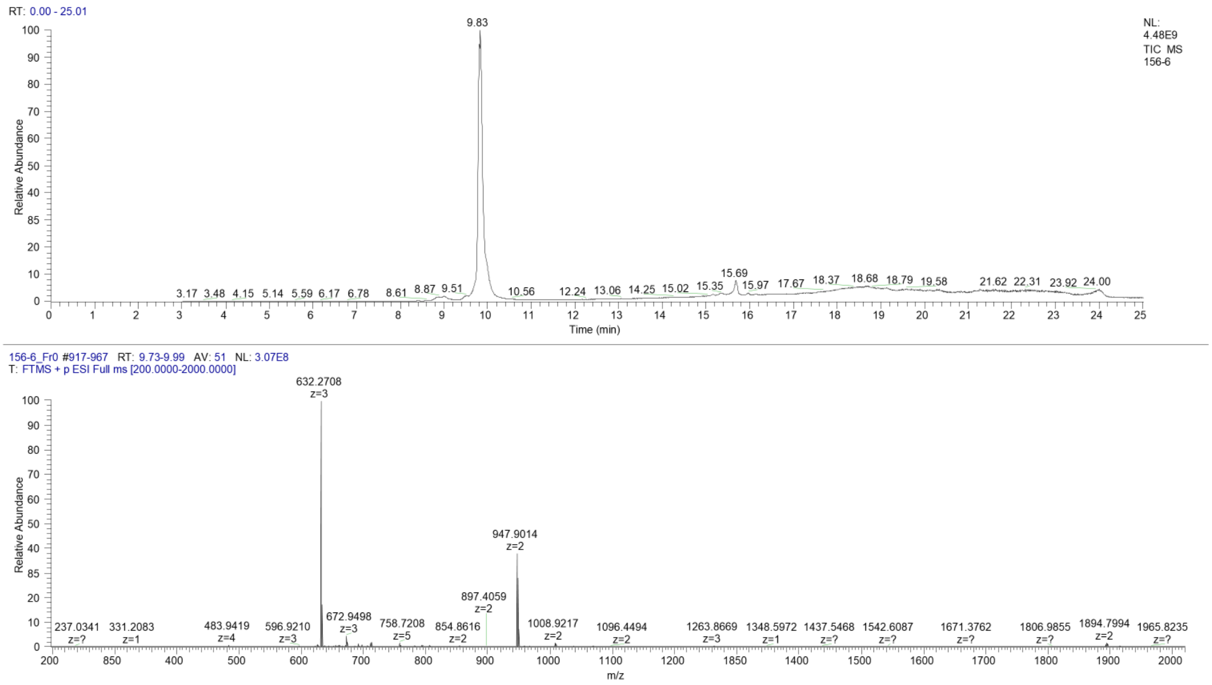

Figure S32: LC-MS Data for 156-6. The TIC chromatogram is shown on top and the extracted masses from the peak are below.

156-7

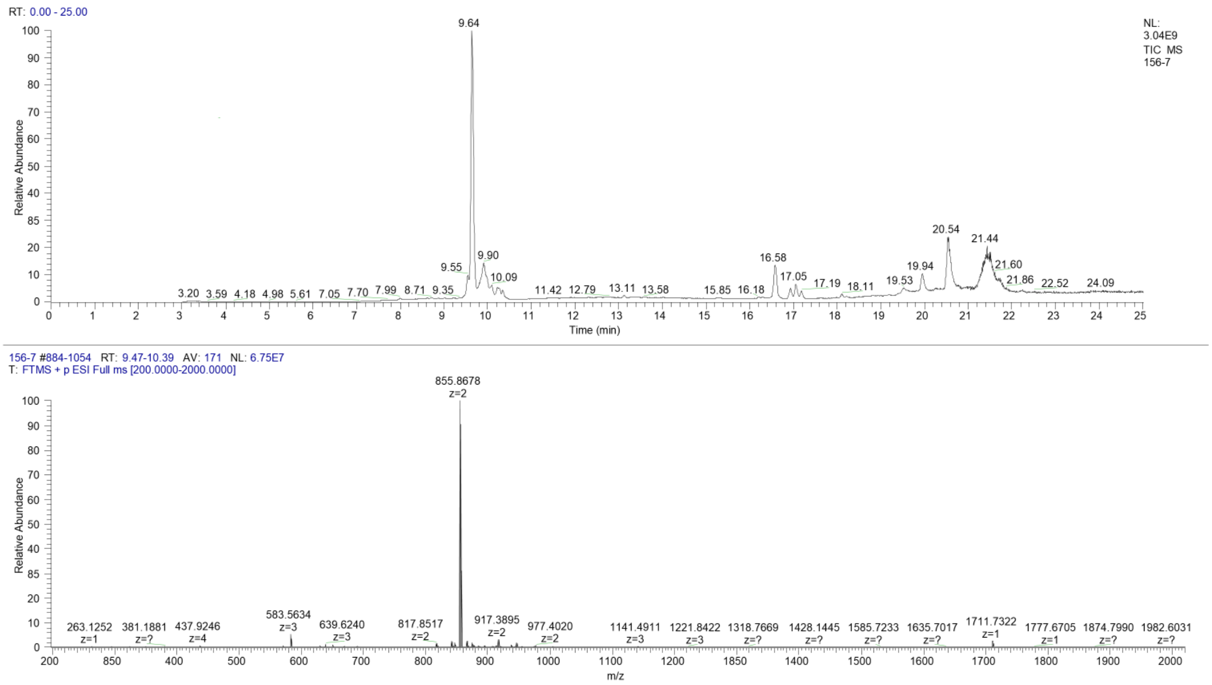

Figure S33: LC-MS Data for 156-7. The TIC chromatogram is shown on top and the extracted masses from the peak are below.

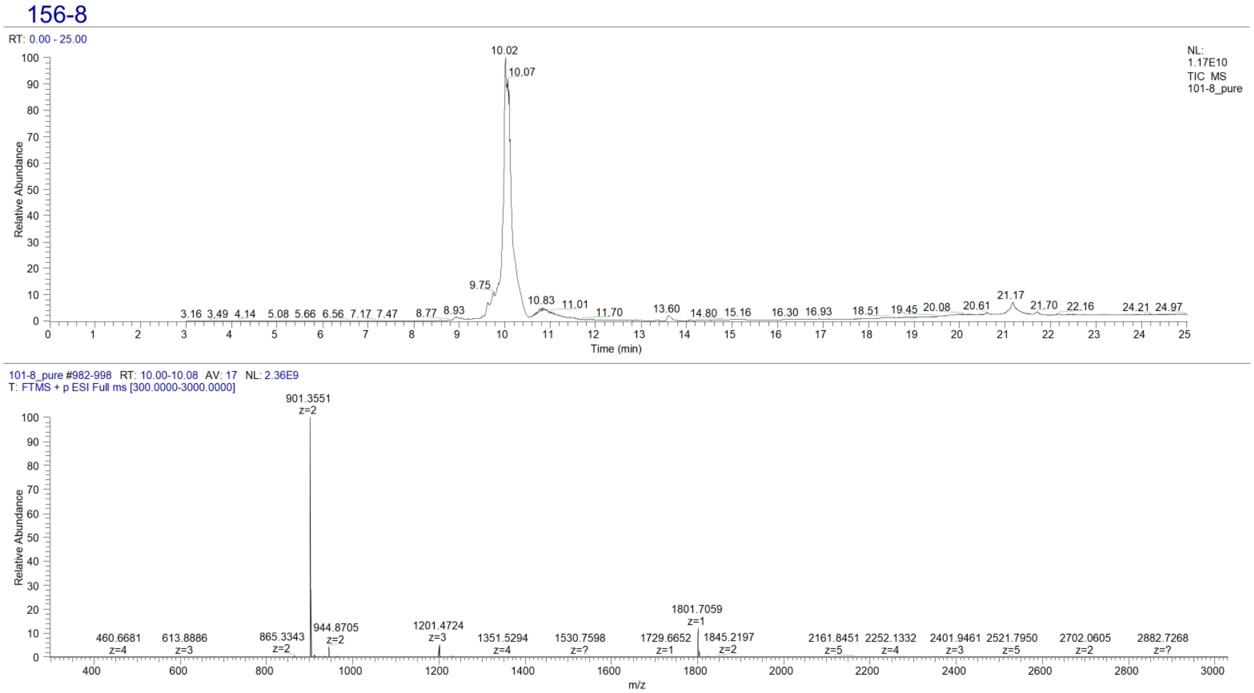

Figure S34: LC-MS Data for 156-8. The TIC chromatogram is shown on top and the extracted masses from the peak are below.

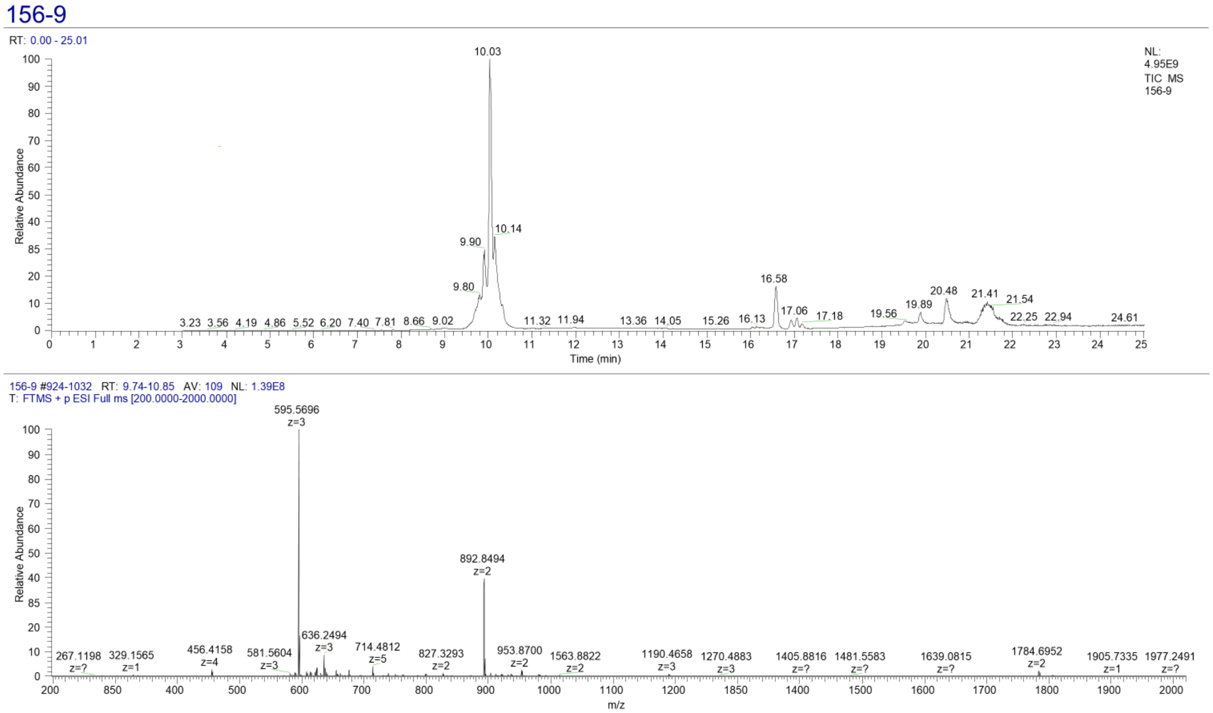

Figure S35: LC-MS Data for 156-9. The TIC chromatogram is shown on top and the extracted masses from the peak are below.

156-10

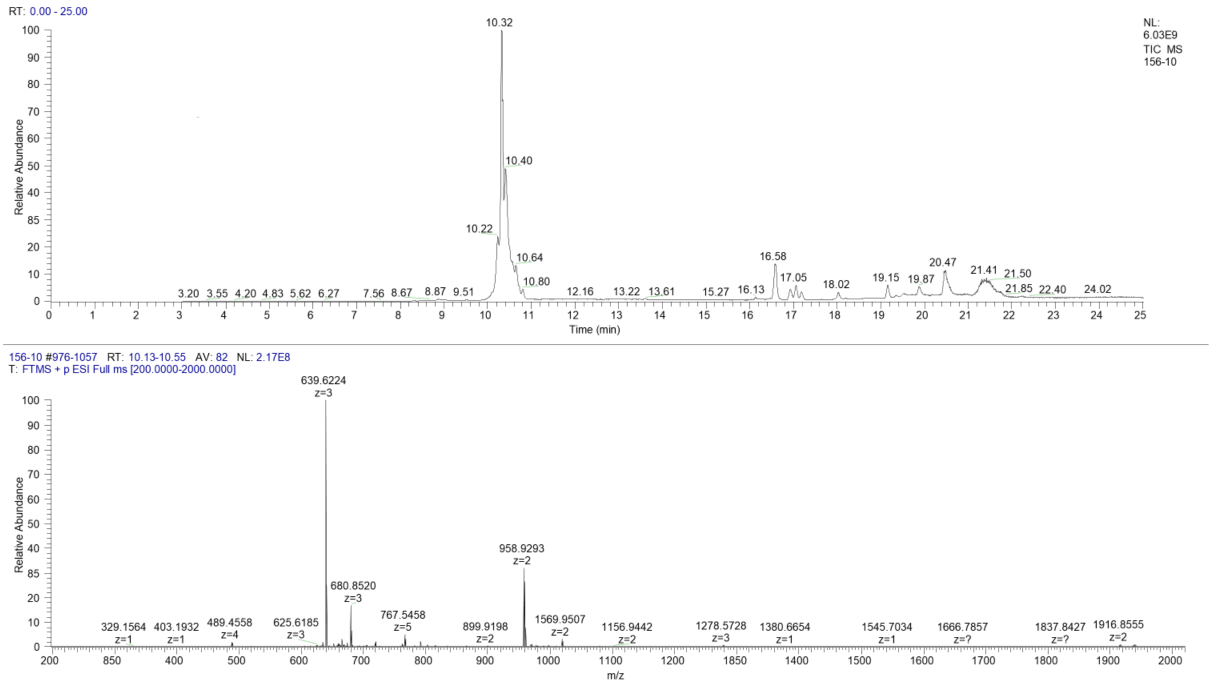

Figure S36: LC-MS Data for 156-10. The TIC chromatogram is shown on top and the extracted masses from the peak are below.

156-11

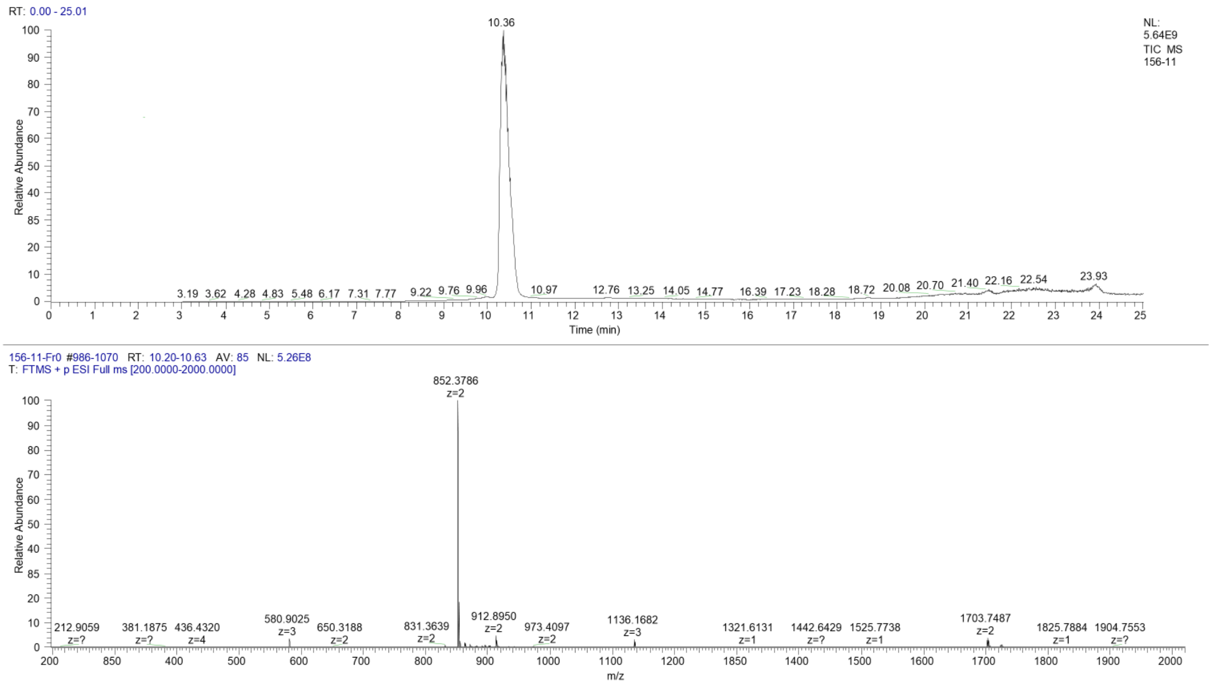

Figure S37: LC-MS Data for 156-11. The TIC chromatogram is shown on top and the extracted masses from the peak are below.

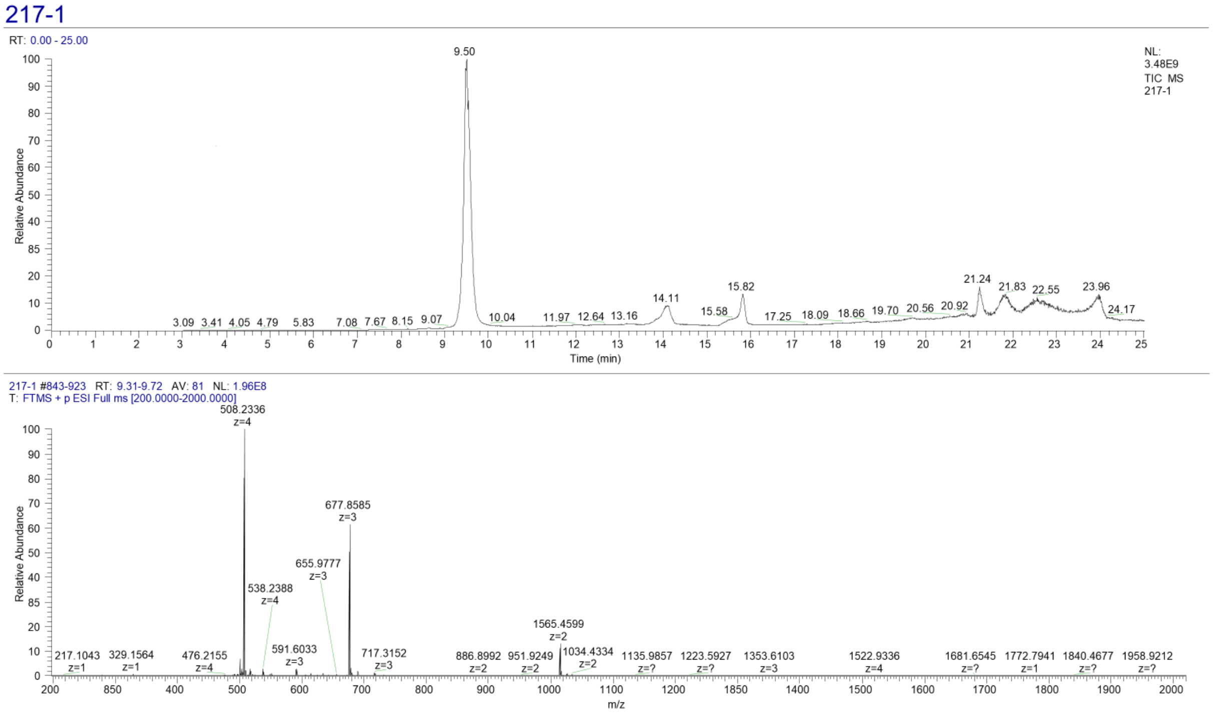

Figure S38: LC-MS Data for 217-1. The TIC chromatogram is shown on top and the extracted masses from the peak are below.

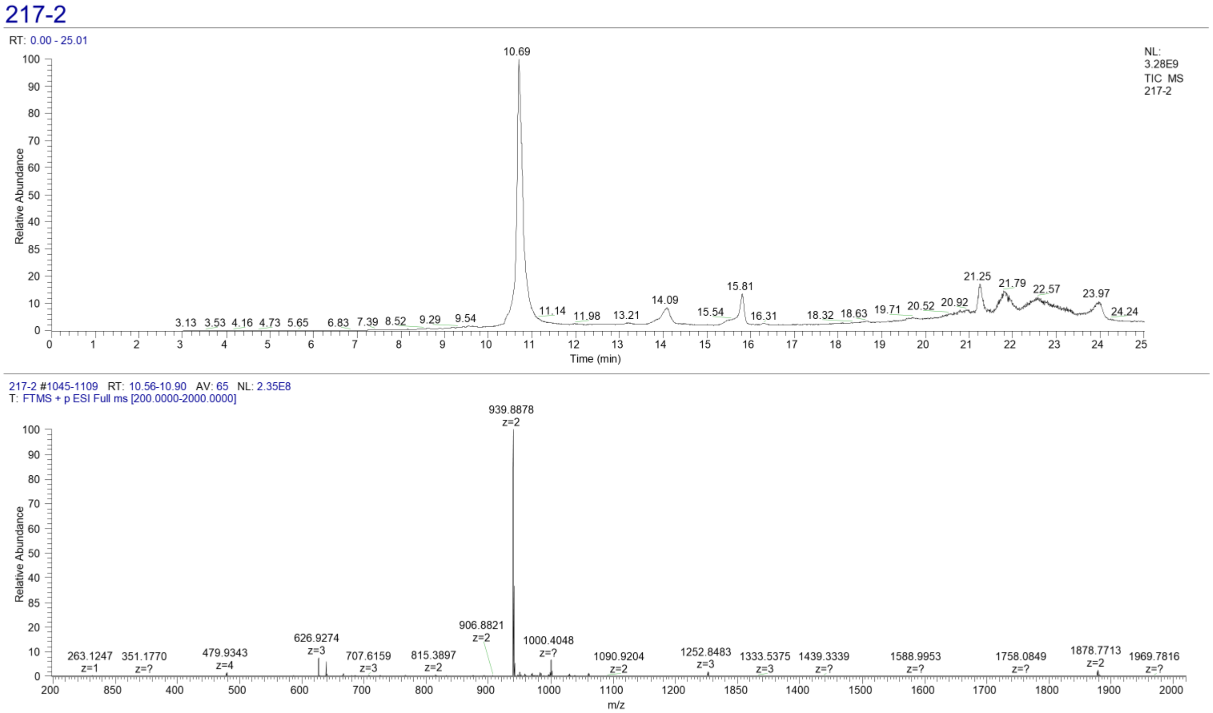

Figure S39: LC-MS Data for 217-2. The TIC chromatogram is shown on top and the extracted masses from the peak are below.

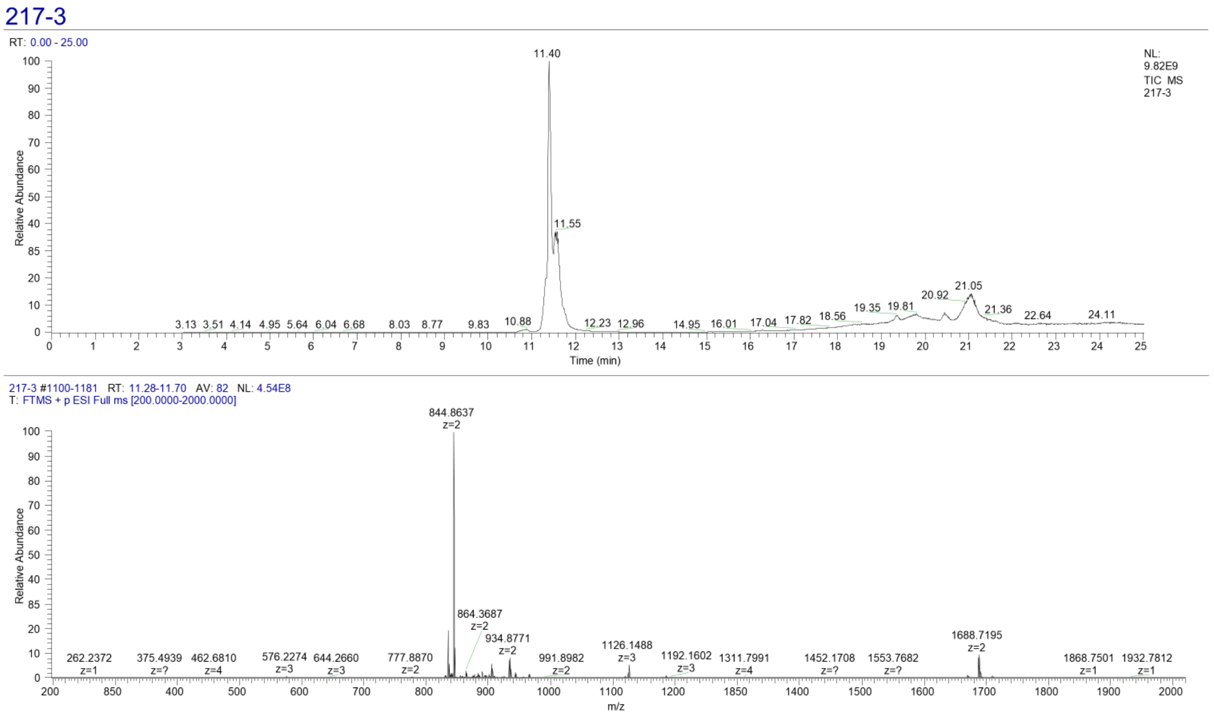

Figure S40: LC-MS Data for 217-3. The TIC chromatogram is shown on top and the extracted masses from the peak are below.

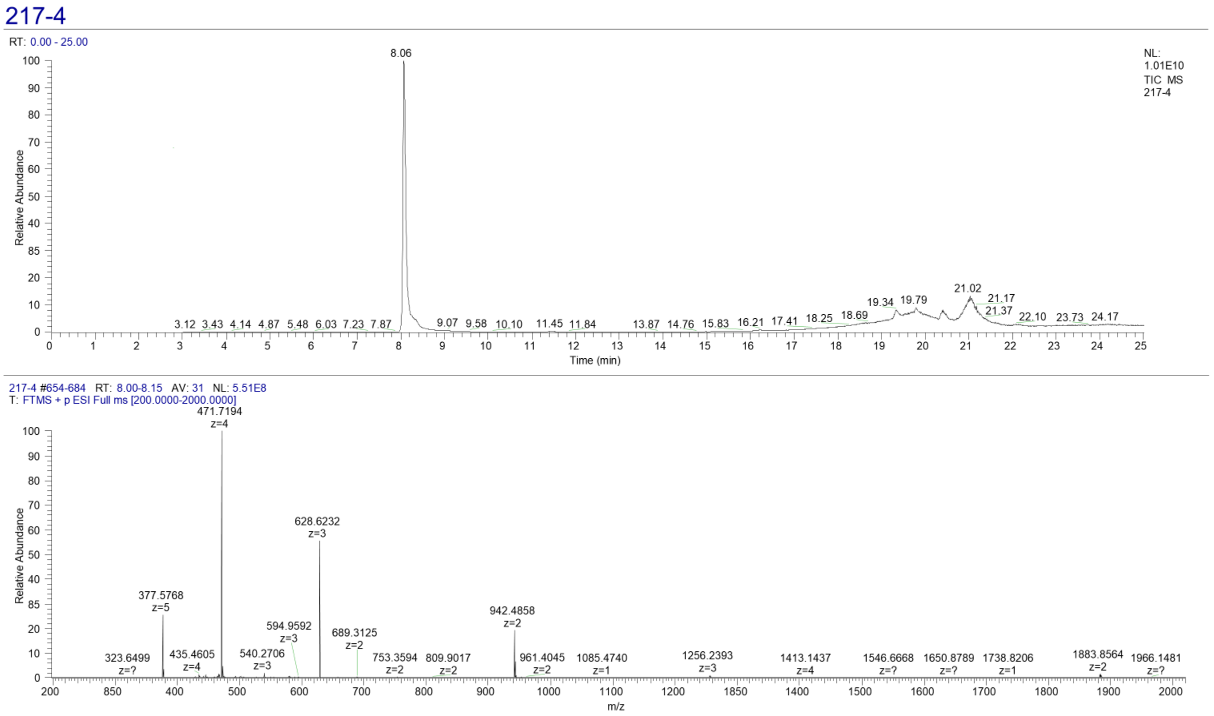

Figure S41: LC-MS Data for 217-4. The TIC chromatogram is shown on top and the extracted masses from the peak are below.

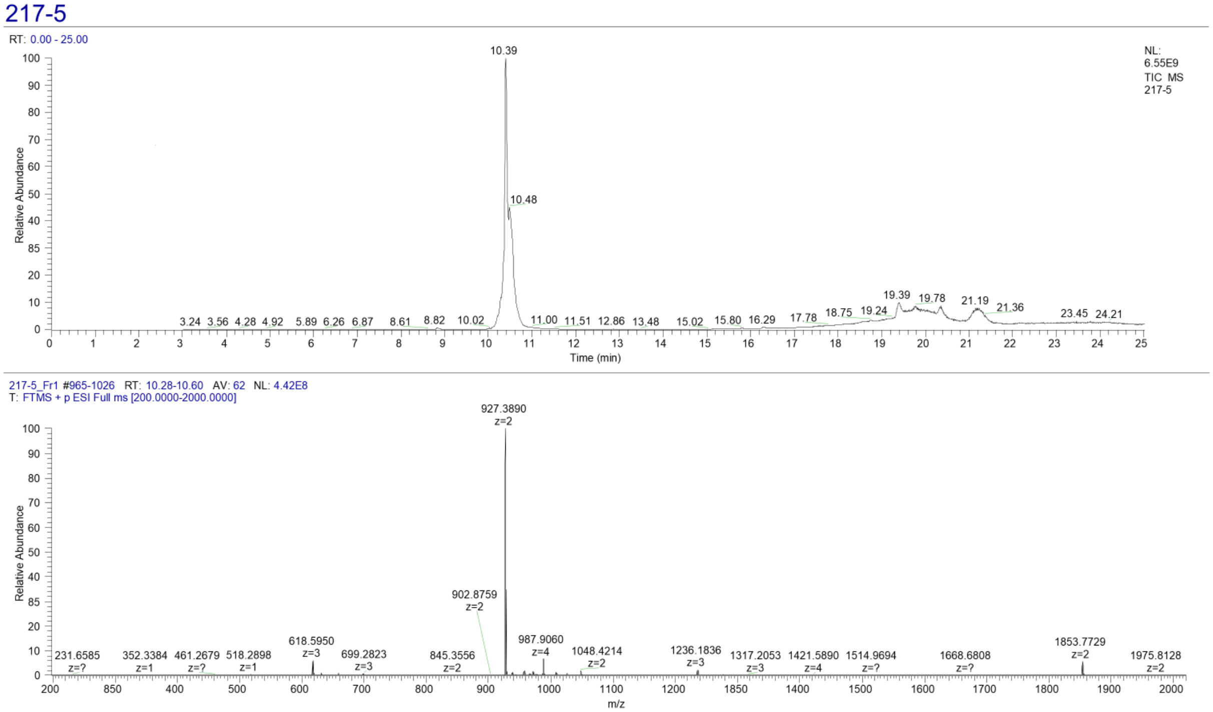

Figure S42: LC-MS Data for 217-5. The TIC chromatogram is shown on top and the extracted masses from the peak are below.

217-6

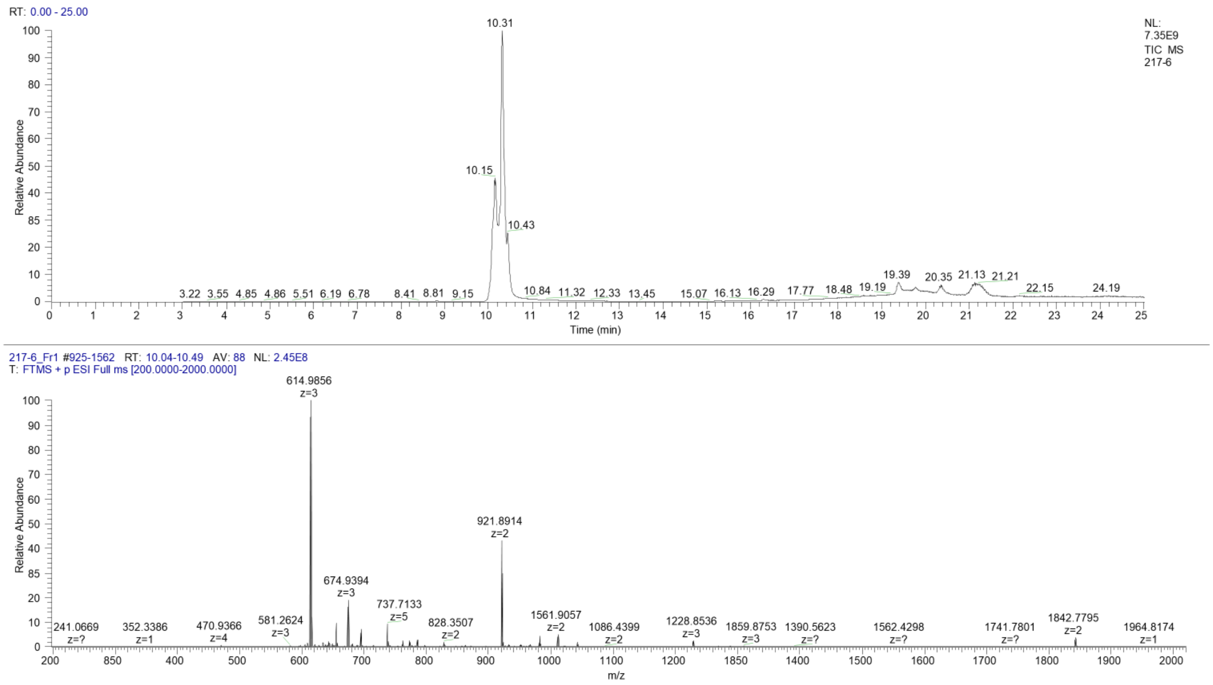

Figure S43: LC-MS Data for 217-6. The TIC chromatogram is shown on top and the extracted masses from the peak are below.

217-7-AbuC

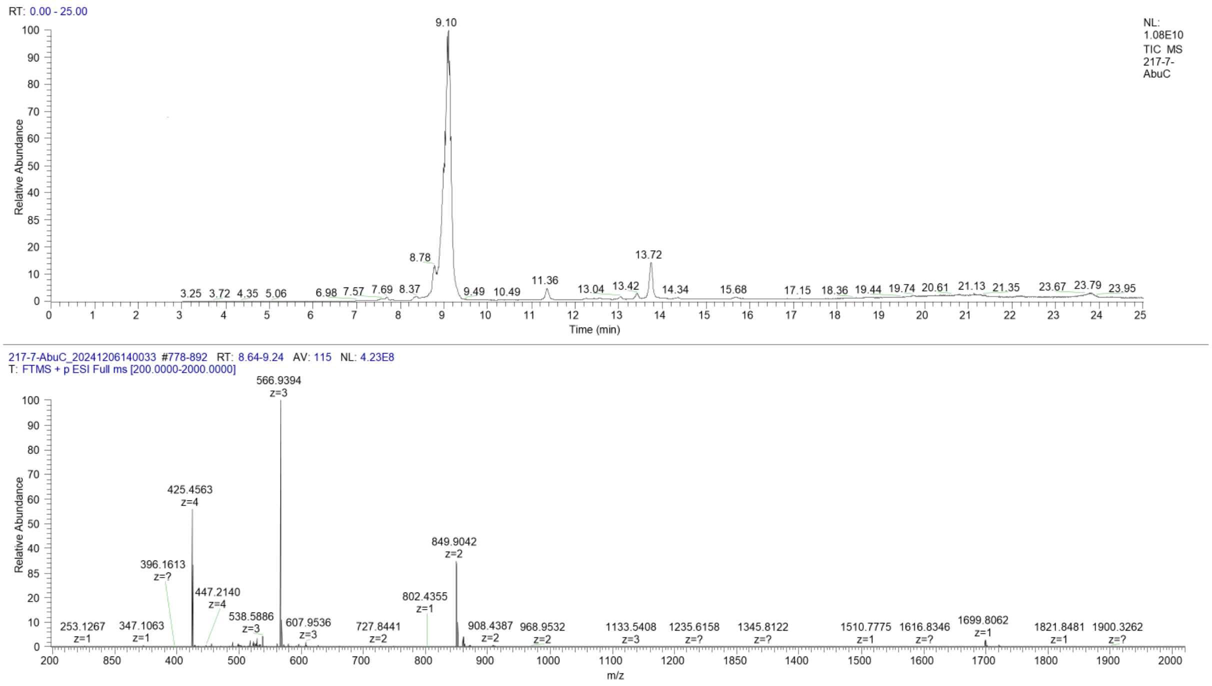

Figure S44: LC-MS Data for 217-7-AbuC. The TIC chromatogram is shown on top and the extracted masses from the peak are below.

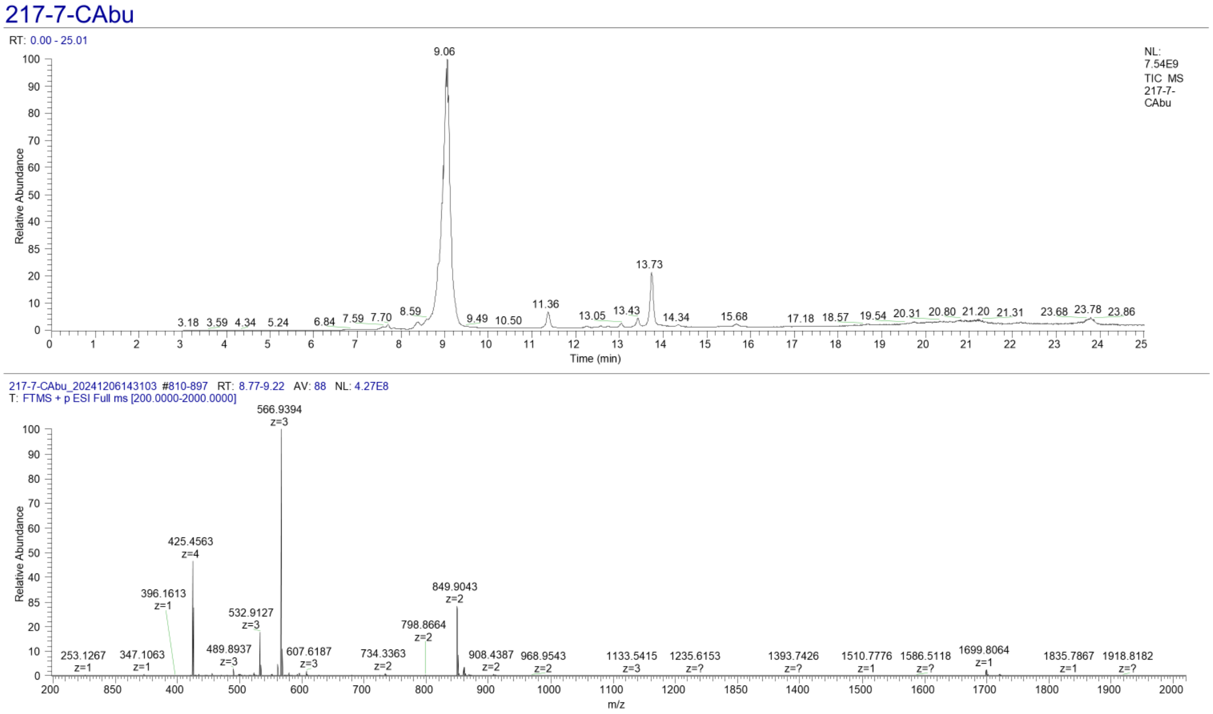

Figure S45: LC-MS Data for 217-7-CAbu. The TIC chromatogram is shown on top and the extracted masses from the peak are below.

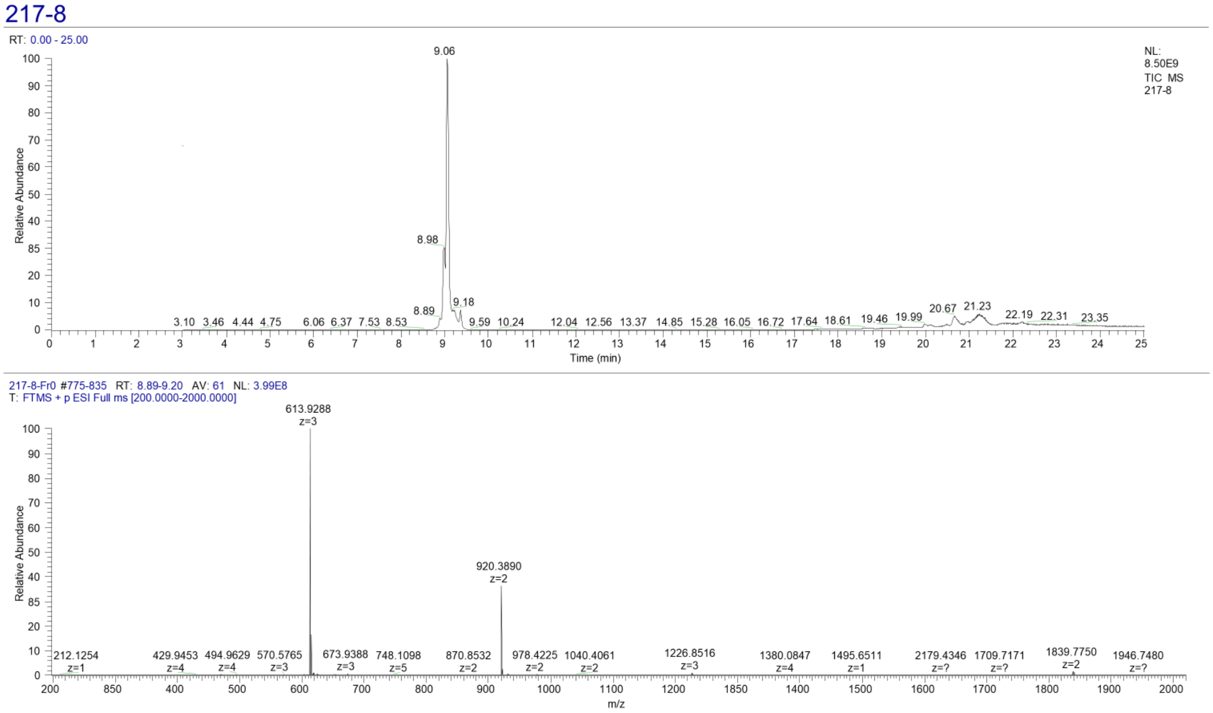

Figure S46: LC-MS Data for 217-8. The TIC chromatogram is shown on top and the extracted masses from the peak are below.

217-9

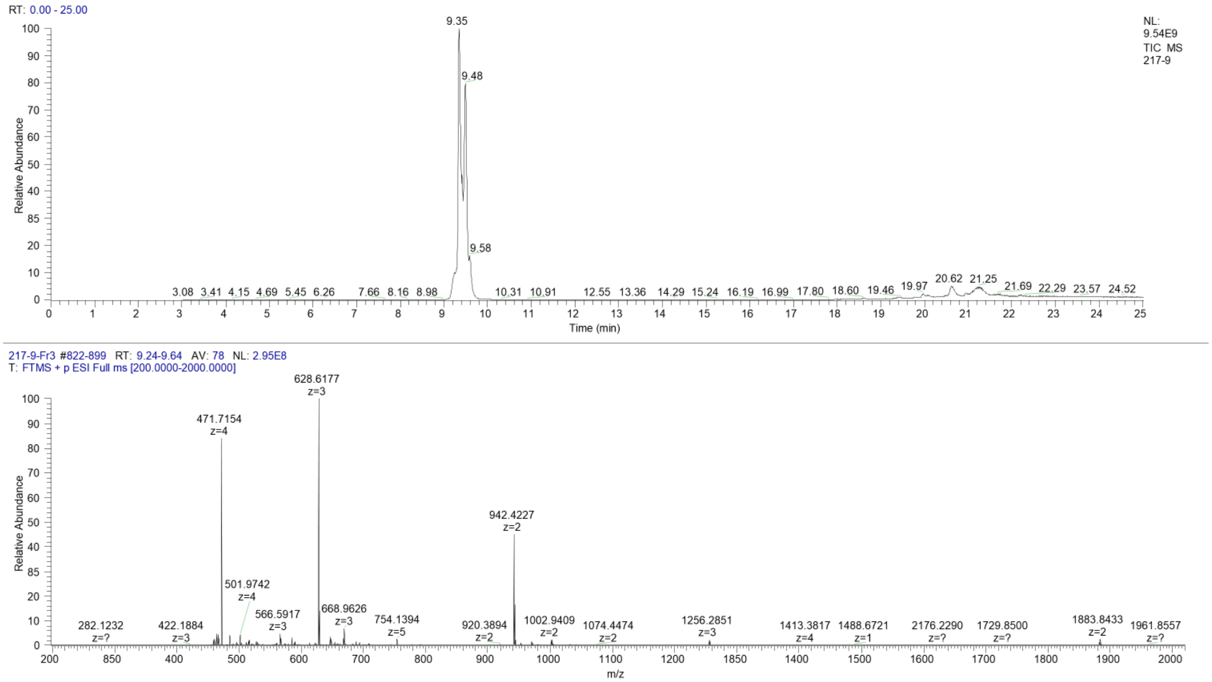

Figure S47: LC-MS Data for 217-9. The TIC chromatogram is shown on top and the extracted masses from the peak are below.

217-10

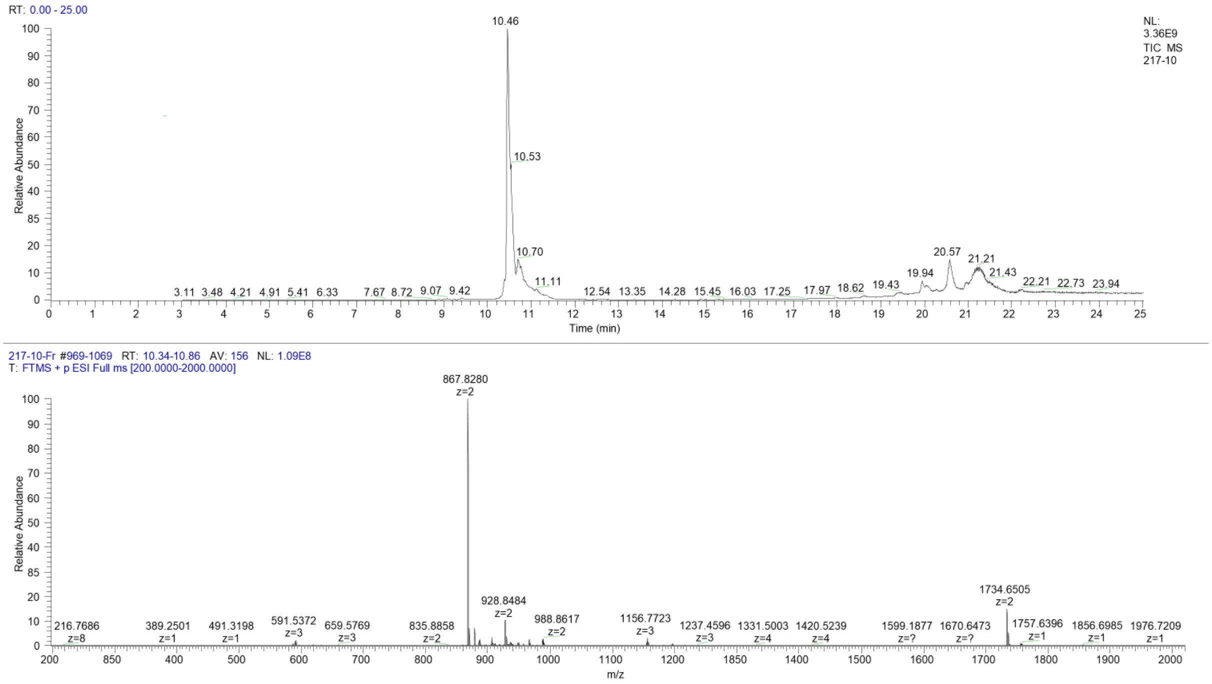

Figure S48: LC-MS Data for 217-10. The TIC chromatogram is shown on top and the extracted masses from the peak are below.

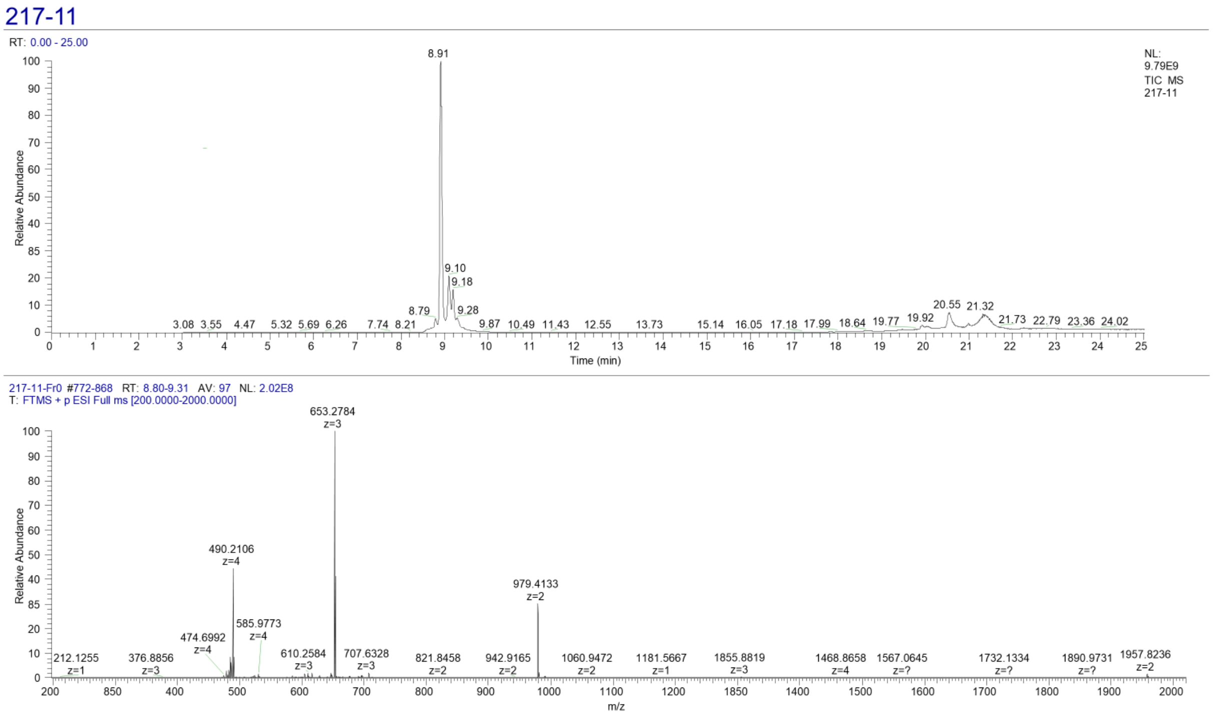

Figure S49: LC-MS Data for 217-11. The TIC chromatogram is shown on top and the extracted masses from the peak are below.

SUPPLEMENTARY TABLES

Table S1: Secondary-structure % estimates and fit metrics derived from far-UV CD spectra using BeStSel (NRMSD)

Protein	Helix (total)	β sheet (total)	Antiparallel (total)	Parallel	Turn	Others	NRMSD
WT	20.5	22.6	17.4	5.2	13.4	43.5	0.01788
F85AzF	22.6	21	16.4	4.6	13.1	43.2	0.01974
F156AzF	30.8	12.2	5	7.2	13.1	44	0.02048
F217AzF	28.1	13.1	8	5.1	14.4	44.5	0.01616

Table S2: Round 3 Sequence Abundance ≥ 0.1%

Sequence	F85 n	F156 n	F217AzF n
**85-1**: CPSWVKAHYGPAAC	21813	12440	13871
**217-1**: CLYHYQWKWWPRYC	210	29	765
**85-2**: CKTHWIWVKWRFVC	995	7	551
**217-3**: CMFPDNLAFVHFFC	459	n.a.	421
CCKLLAYGWVSCWC	229	50	330
**217-2**: CNMWPWWSYSYRVC	120	2	284

CVKAPYKWRTWFIC	49	6	275
CGTAHPYSWHRWWC	24	1	260
CTNYVKNTFWYWYC	83	4	249
**217-4**: CYIKHMWHWHRVSC	268	n.a.	213
CDFWWQYKFGPRWC	7	n.a.	209
**217-7**: CSKFPMSKRFPWCC	n.a.	n.a.	209
CSWHKPHWLWDRWC	51	15	208
CHSSWSPYWTRWWC	11	n.a.	153
CQRSHYWWGGWHSC	71	n.a.	151
CDSHPTWFTWRWGC	33	n.a.	151
CHVKYPALFWWKWC	5	n.a.	151
CEQWVRIKWGFWVC	15	1	145
CKCLRIKAWAYCLC	3	n.a.	145
CWSGRIPYWWASYC	70	6	130
CKYWYGHKYFWRWC	26	n.a.	130
CYSWYGWRYYGWYC	57	6	129
CGNKYHGYWYSIYC	33	2	129
CSPRAPYWYWTWVC	3	n.a.	125
CHWYWHSSGYWRHC	34	n.a.	123
CKSPYSWRWFGWWC	1	n.a.	119
CKRIYVPWYWSYYC	17	1	110
**85-12**: CFPWWDGHTWHLFC	311	54	107
CPYSFPWRWGYSWC	149	2	105
CNDGGRWHWYWWGC	15	3	99
**85-6**: CHKTWPVWYAKWWC	246	n.a.	98
CKWGRWMSWGMWDC	178	n.a.	98
CSTPQMWYWHYRYC	21	4	98
**217-8**: CELVMMHGYWWRQC	n.a.	n.a.	95
**217-9**: CKYGWHWLWGYRQC	n.a.	n.a.	91
CNYYWYSPHVWRYC	10	n.a.	90
CICRAHYYFWYCPC	n.a.	n.a.	89
CYTSYPYYWTRGWC	55	2	86
CHWTWPYWYHSRWC	62	n.a.	85
CFYGKGWWYPSAWC	73	n.a.	83
CYKAGHWWRYSWYC	42	n.a.	83
CFSKCYCVHRYCFC	n.a.	9	80
CYWYWPYMSKVRHC	87	n.a.	78
CTWNTGYWWTRWSC	43	n.a.	78
CSRIVKGWSYCKVC	4	9	77
**156-2**: CPSWVNAHYGPAAC	114	61	71
CPSCVKAHYGPAAC	85	57	69
CKYPWWVMRWYPQC	85	3	48
CQSPYLWWRYSAYC	99	1	46
CWSKCTRYYCYSVC	83	n.a.	45
CQGWWQHSRWSPWC	173	4	40

CWNGHCTWRQWWCC	87	n.a.	35
CVCTKFMSKFFCWC	176	3	24
CPTWYGVHFSSDEC	107	4	21
**85-5**: CPMWLERHFGPEAC	662	n.a.	15
CHWWCFAGYCRFGC	86	1	14
CVNGAFWWKHGYWC	130	n.a.	6
CYFGHWGWAYSRYC	105	n.a.	5
**85-3**: CHKGAWWWYSSWRC	96	9	n.a.
**156-1**: CWFTTDTHGVYYIC	68	114	48
**156-7**: CDLKYYNVYGIEMC	n.a.	475	n.a.
**156-8**: CHWPSSSYAYQWWC	n.a.	173	n.a.

Table S3: High-Resolution Mass Spectrometry Data for Synthesized Peptides

Peptide	Sequence	Expected Mass (Da)	Observed Mass (Da)
85-1	CPSWVKAHYGPAAC	1485.66	1485.67
85-2	CKTHWIWVKWRFVC	1888.97	1888.97
85-3	CHKGAWWWYSSWRC	1852.79	1852.80
85-4	CHMARQSFWSAWFC	1756.73	1756.73
85-5	CPMWLERHFGPEAC	1672.71	1672.72
85-6	CHKTWPVWYAKWWC	1890.87	1890.88
85-7	CWTPYFWKAGWVGC	1700.75	1700.76
85-8	CFYTGGWSWAMRGC	1620.64	1620.65
85-9	CHHFPMRSLWWYLC	1875.84	1875.84
85-10	CHRSQYSWMANFWC	1815.73	1815.73
85-11	CFTRYHWYWWRTEC	2033.86	2033.87
85-12	CFPWWDGHTWHLFC	1831.76	1831.77
156-1	CWFTTDTHGVYYIC	1705.71	1705.72
156-2	CPSWVNAHYGPAAC	1471.61	1471.62
156-3	CRVLALHWSSDLPC	1595.77	1595.78
156-4	CYGMYWYWRNTPVC	1838.76	1838.76
156-5	CYKASSYWYYPWYC	1879.76	1879.76
156-6	CKAYWNMWWSQKYC	1893.80	1893.80
156-7	CDLKYYNVYGIEMC	1709.73	1709.72
156-8	CHWPSSSYAYQWWC	1800.7	1800.71
156-9	CHQSSWSMWSKWMC	1783.69	1783.70
156-10	CFWAKSQYWKPWWC	1915.85	1915.86
156-11	CSSQPFTWWGKIYC	1702.75	1702.76
217-1	CLYHYQWKWWPRYC	2028.91	2028.92
217-2	CNMWPWWSYSYRVC	1877.77	1877.78
217-3	CMFPDNLAFVHFFC	1687.72	1687.73
217-4	CYIKHMWHWHRVSC	1882.85	1882.86
217-5	CYWYSPSVRYWGWC	1852.77	1852.78
217-6	CHSSYWWFVNRWAC	1841.77	1841.78
217-7	CSKFPMSKRFPWAbuC	1697.80	1697.81

217-8	CELVMMHGYWWRQC	1838.77	1838.78
217-9	CKYGWHWLWGYRQC	1882.84	1882.85
217-10	CFYYTFSSGWQNMC	1733.65	1733.66
217-11	CYWLMHRYHAYWQC	1956.82	1956.83

Abu: Aminobutyric acid in place of Cysteine

Table S4: HPLC-MS Purification Conditions for peptides

Peptide	Gradient*	Column	Ionization Mode	Flow Rate (mL/min)
85-1	0-15%ACN in water over 25 min	C18, 250x20 mm	ESI (+)	15
85-2	15-50%ACN in water over 25 min	C18, 250x20 mm	ESI (+)	15
85-3	10-60%ACN in water over 25 min	C18, 250x20 mm	ESI (+)	15
85-4	10-15%ACN in water over 25 min	C18, 250x20 mm	ESI (+)	15
85-5	0-55%ACN in water over 25 min	C18, 250x20 mm	ESI (+)	15
85-6	0-55%ACN in water over 25 min	C18, 250x20 mm	ESI (+)	15
85-7	15-60%ACN in water over 25 min	C18, 250x20 mm	ESI (+)	15
85-8	15-60%ACN in water over 25 min	C18, 250x20 mm	ESI (+)	15
85-9	15-60%ACN in water over 25 min	C18, 250x20 mm	ESI (+)	15
85-10	15-60%ACN in water over 25 min	C18, 250x20 mm	ESI (+)	15
85-11	0-50%ACN in water over 25 min	C18, 250x20 mm	ESI (+)	15
85-12	15-65%ACN in water over 30 min	C18, 250x20 mm	ESI (+)	15
156-1	15-60%ACN in water over 25 min	C18, 250x20 mm	ESI (+)	15
156-2	0-25%ACN in water over 25 min	C18, 250x20 mm	ESI (+)	15
156-3	15-60%ACN in water over 25 min	C18, 250x20 mm	ESI (+)	15
156-4	15-60%ACN in water over 25 min	C18, 250x20 mm	ESI (+)	15
156-5	10-60%ACN in water over 25 min	C18, 250x20 mm	ESI (+)	15
156-6	5-40%ACN in water over 25 min	C18, 250x20 mm	ESI (+)	15
156-7	15-60%ACN in water over 25 min	C18, 250x20 mm	ESI (+)	15
156-8	10-60%ACN in water over 25 min	C18, 250x20 mm	ESI (+)	15
156-9	10-60%ACN in water over 25 min	C18, 250x20 mm	ESI (+)	15
156-10	10-60%ACN in water over 25 min	C18, 250x20 mm	ESI (+)	15
156-11	15-60%ACN in water over 25 min	C18, 250x20 mm	ESI (+)	15
217-1	0-30%ACN in water over 25 min	C18, 250x20 mm	ESI (+)	15
217-2	10-50%ACN in water over 25 min	C18, 250x20 mm	ESI (+)	15
217-3	15-60%ACN in water over 25 min	C18, 250x20 mm	ESI (+)	15
217-4	0-35%ACN in water over 25 min	C18, 250x20 mm	ESI (+)	15
217-5	10-60%ACN in water over 25 min	C18, 250x20 mm	ESI (+)	15
217-6	10-60%ACN in water over 25 min	C18, 250x20 mm	ESI (+)	15
217-7	10-60%ACN in water over 25 min	C18, 250x20 mm	ESI (+)	15
217-8	15-50%ACN in water over 25 min	C18, 250x20 mm	ESI (+)	15
217-9	10-50%ACN in water over 25 min	C18, 250x20 mm	ESI (+)	15
217-10	0-60%ACN in water over 25 min	C18, 250x20 mm	ESI (+)	15
217-11	10-70%ACN in water over 25 min	C18, 250x20 mm	ESI (+)	15

*All solvents contained 0.1% formic acid as an additive

SUPPLEMENTARY SCRIPTS

Supplementary Script 1: Amino Acid Analysis

```
library(microseq)
library(RColorBrewer)
library(dplyr)
library(stringr)
library(gplots)
NNK7Ffilt<-readFastq("C:/Users/demon//Documents//NGS/F101/23316Wns_F101-
R1_S4_L001_R1_001.fastq")
NNK7Rfilt<-readFastq("C:/Users/demon//Documents//NGS/F101/23316Wns_F101-
R1_S4_L001_R2_001.fastq")
#Define the following variables
libraryseq <- "GCCCAG.{54}GCGGCG.{6}" #change this regex to match specific library
beginning <- 19 #beginning of library in DNA string
lib <- 14 #number of codons in the library region
end <- beginning+lib*3
initialcodon <- beginning%/%3*4-1
endcodon <- initialcodon + lib*4
del <- beginning%/%3 #number of codons before library
aa <- c("A","C","D","E","F","G","H","I","K","L","M","N","P","Q","R","S","T","V","W","Y","TAG")

#slices out matches that contain start followed by 24 bases to reverse primer
NNK7Ffilt21 <- gregexpr(libraryseq,NNK7Ffilt[[2]],extract = TRUE)
NNK7Rrevcomp <- reverseComplement(NNK7Rfilt[[2]],reverse = TRUE) #gives reverse complement of
reverse reads
NNK7Rcompfilt21 <- gregexpr(libraryseq,NNK7Rrevcomp,extract = TRUE)

#this compares the forward and reverse strands, only allowing for one mismatch in the primers, no
mismatches allowed in the library region
n <- length(NNK7Ffilt21)
NNK7Fgood <- vector()
for(i in c(1:n)){
  if(NNK7Ffilt21[[i]][1] == NNK7Rcompfilt21[[i]][1]){
    NNK7Fgood[i] <- NNK7Ffilt21[[i]]
  }
  else{
    split <- strsplit(c(NNK7Ffilt21[[i]],NNK7Rcompfilt21[[i]]), split = "")
    diff <- which(split[[1]] != split[[2]])
    if(length(diff) < 2 && length(diff) > 0){
      for(x in c(1:length(diff))){
        if(diff[[x]] < beginning || diff[[x]] > end){
          NNK7Fgood[i] <- NNK7Ffilt21[[i]]
        }
        else{
          NNK7Fgood[i] <- ""
        }
      }
    }
    else{
```

```
      NNK7Fgood[i] <- ""
    }
  }
}
NNK7Fgood <- as.data.frame(NNK7Fgood)
NNK7Fgood <- NNK7Fgood[!apply(is.na(NNK7Fgood) | NNK7Fgood == "", 1, all),]

#this separates nucleotides into codons
codons <- gsub("(...)", "\\1 \\2", NNK7Fgood)

#this creates dataframe of sequences with reads organized by frequency
seqcount <- as.data.frame(sort(table(codons), decreasing = TRUE))

#this generates a matrix that contains amino acids in library region
l <- length(codons)
AAs <- matrix(0,l,lib)
AA <- gregexpr("\\s(TT[TC])",codons,useBytes = FALSE)
  l <- length(AA)
  for(a in c(1:l)){
    l2 <- length(AA[[a]])
    for(b in c(1:l2)){
      value <- AA[[a]][b]
      if(value > initialcodon && value < endcodon){
        AAs[a,(value%/%4 - (del-1))] <- "F"
      }
    }
  }
AA <- gregexpr("(\\sTT[AG])|(\\sCT[GACT])",codons,useBytes = FALSE)
  l <- length(AA)
  for(a in c(1:l)){
    l2 <- length(AA[[a]])
    for(b in c(1:l2)){
      value <- AA[[a]][b]
      if(value > initialcodon && value < endcodon){
        AAs[a,(value%/%4 - (del-1))] <- "L"
      }
    }
  }
AA <- gregexpr("(\\sTC[GCAT])|(\\sAG[TC])",codons,useBytes = FALSE)
  l <- length(AA)
  for(a in c(1:l)){
    l2 <- length(AA[[a]])
    for(b in c(1:l2)){
      value <- AA[[a]][b]
      if(value > initialcodon && value < endcodon){
        AAs[a,(value%/%4 - (del-1))] <- "S"
      }
    }
  }
AA <- gregexpr("\\sTA[TC]",codons,useBytes = FALSE)
  l <- length(AA)
```

```
  for(a in c(1:l)){
    l2 <- length(AA[[a]])
    for(b in c(1:l2)){
      value <- AA[[a]][b]
      if(value > initialcodon && value < endcodon){
        AAs[a,(value%/%4 - (del-1))] <- "Y"
      }
    }
  }
AA <- gregexpr("\\sTAG",codons,useBytes = FALSE)
  l <- length(AA)
  for(a in c(1:l)){
    l2 <- length(AA[[a]])
    for(b in c(1:l2)){
      value <- AA[[a]][b]
      if(value > initialcodon && value < endcodon){
        AAs[a,(value%/%4 - (del-1))] <- "TAG"
      }
    }
  }
AA <- gregexpr("\\sTAA",codons,useBytes = FALSE)
  l <- length(AA)
  for(a in c(1:l)){
    l2 <- length(AA[[a]])
    for(b in c(1:l2)){
      value <- AA[[a]][b]
      if(value > initialcodon && value < endcodon){
        AAs[a,(value%/%4 - (del-1))] <- NA
      }
    }
  }
AA <- gregexpr("\\sTG[TC]",codons,useBytes = FALSE)
  l <- length(AA)
  for(a in c(1:l)){
    l2 <- length(AA[[a]])
    for(b in c(1:l2)){
      value <- AA[[a]][b]
      if(value > initialcodon && value < endcodon){
        AAs[a,(value%/%4 - (del-1))] <- "C"
      }
    }
  }
AA <- gregexpr("\\sTGA",codons,useBytes = FALSE)
  l <- length(AA)
  for(a in c(1:l)){
    l2 <- length(AA[[a]])
    for(b in c(1:l2)){
      value <- AA[[a]][b]
      if(value > initialcodon && value < endcodon){
        AAs[a,(value%/%4 - (del-1))] <- NA
      }
```

```
    }
  }
AA <- gregexpr("\\sTGG",codons,useBytes = FALSE)
  l <- length(AA)
  for(a in c(1:l)){
    l2 <- length(AA[[a]])
    for(b in c(1:l2)){
      value <- AA[[a]][b]
      if(value > initialcodon && value < endcodon){
        AAs[a,(value%/%4 - (del-1))] <- "W"
      }
    }
  }
AA <- gregexpr("\\sCC[GCAT]",codons,useBytes = FALSE)
  l <- length(AA)
  for(a in c(1:l)){
    l2 <- length(AA[[a]])
    for(b in c(1:l2)){
      value <- AA[[a]][b]
      if(value > initialcodon && value < endcodon){
        AAs[a,(value%/%4 - (del-1))] <- "P"
      }
    }
  }
AA <- gregexpr("\\sCA[CT]",codons,useBytes = FALSE)
  l <- length(AA)
  for(a in c(1:l)){
    l2 <- length(AA[[a]])
    for(b in c(1:l2)){
      value <- AA[[a]][b]
      if(value > initialcodon && value < endcodon){
        AAs[a,(value%/%4 - (del-1))] <- "H"
      }
    }
  }
AA <- gregexpr("\\sCA[AG]",codons,useBytes = FALSE)
  l <- length(AA)
  for(a in c(1:l)){
    l2 <- length(AA[[a]])
    for(b in c(1:l2)){
      value <- AA[[a]][b]
      if(value > initialcodon && value < endcodon){
        AAs[a,(value%/%4 - (del-1))] <- "Q"
      }
    }
  }
AA <- gregexpr("(\\sCG[GCAT])|(\\sAG[GA])",codons,useBytes = FALSE)
  l <- length(AA)
  for(a in c(1:l)){
    l2 <- length(AA[[a]])
    for(b in c(1:l2)){
```

```
      value <- AA[[a]][b]
      if(value > initialcodon && value < endcodon){
        AAs[a,(value%/%4 - (del-1))] <- "R"
      }
    }
  }
AA <- gregexpr("\\sAT[CAT]",codons,useBytes = FALSE)
  l <- length(AA)
  for(a in c(1:l)){
    l2 <- length(AA[[a]])
    for(b in c(1:l2)){
      value <- AA[[a]][b]
      if(value > initialcodon && value < endcodon){
        AAs[a,(value%/%4 - (del-1))] <- "I"
      }
    }
  }
AA <- gregexpr("\\sATG",codons,useBytes = FALSE)
  l <- length(AA)
  for(a in c(1:l)){
    l2 <- length(AA[[a]])
    for(b in c(1:l2)){
      value <- AA[[a]][b]
      if(value > initialcodon && value < endcodon){
        AAs[a,(value%/%4 - (del-1))] <- "M"
      }
    }
  }
AA <- gregexpr("\\sAC[GCAT]",codons,useBytes = FALSE)
  l <- length(AA)
  for(a in c(1:l)){
    l2 <- length(AA[[a]])
    for(b in c(1:l2)){
      value <- AA[[a]][b]
      if(value > initialcodon && value < endcodon){
        AAs[a,(value%/%4 - (del-1))] <- "T"
      }
    }
  }
AA <- gregexpr("\\sAA[CT]",codons,useBytes = FALSE)
  l <- length(AA)
  for(a in c(1:l)){
    l2 <- length(AA[[a]])
    for(b in c(1:l2)){
      value <- AA[[a]][b]
      if(value > initialcodon && value < endcodon){
        AAs[a,(value%/%4 - (del-1))] <- "N"
      }
    }
  }
AA <- gregexpr("\\sAA[AG]",codons,useBytes = FALSE)
```

```
  l <- length(AA)
  for(a in c(1:l)){
    l2 <- length(AA[[a]])
    for(b in c(1:l2)){
      value <- AA[[a]][b]
      if(value > initialcodon && value < endcodon){
        AAs[a,(value%/%4 - (del-1))] <- "K"
      }
    }
  }
AA <- gregexpr("\\sGT[GACT]",codons,useBytes = FALSE)
  l <- length(AA)
  for(a in c(1:l)){
    l2 <- length(AA[[a]])
    for(b in c(1:l2)){
      value <- AA[[a]][b]
      if(value > initialcodon && value < endcodon){
        AAs[a,(value%/%4 - (del-1))] <- "V"
      }
    }
  }
AA <- gregexpr("\\sGC[GACT]",codons,useBytes = FALSE)
  l <- length(AA)
  for(a in c(1:l)){
    l2 <- length(AA[[a]])
    for(b in c(1:l2)){
      value <- AA[[a]][b]
      if(value > initialcodon && value < endcodon){
        AAs[a,(value%/%4 - (del-1))] <- "A"
      }
    }
  }
AA <- gregexpr("\\sGA[TC]",codons,useBytes = FALSE)
  l <- length(AA)
  for(a in c(1:l)){
    l2 <- length(AA[[a]])
    for(b in c(1:l2)){
      value <- AA[[a]][b]
      if(value > initialcodon && value < endcodon){
        AAs[a,(value%/%4 - (del-1))] <- "D"
      }
    }
  }
AA <- gregexpr("\\sGA[AG]",codons,useBytes = FALSE)
  l <- length(AA)
  for(a in c(1:l)){
    l2 <- length(AA[[a]])
    for(b in c(1:l2)){
      value <- AA[[a]][b]
      if(value > initialcodon && value < endcodon){
        AAs[a,(value%/%4 - (del-1))] <- "E"
```

```
      }
    }
  }
AA <- gregexpr("\\sGG[GACT]",codons,useBytes = FALSE)
  l <- length(AA)
  for(a in c(1:l)){
    l2 <- length(AA[[a]])
    for(b in c(1:l2)){
      value <- AA[[a]][b]
      if(value > initialcodon && value < endcodon){
        AAs[a,(value%/%4 - (del-1))] <- "G"
      }
    }
  }
AAs <- as.data.frame(AAs)
#this gives unique amino acid sequences
UniqueAAs <- AAs %>% group_by_all() %>% count()
UniqueAAs <- UniqueAAs[order(-UniqueAAs$n),]
UniqueAAs <- UniqueAAs[apply(UniqueAAs,1,function(row) all(row != 0)),]
UniqueAAs <- na.omit(UniqueAAs)
F101UniqueAAsR2 <- UniqueAAs

#this counts sequences that have TAG codons, sequences that have more than one are only counted once
TAGreg <- regexpr("\\sTAG",codons)
TAGtable <- table(TAGreg)
percentTAG <- sum(TAGtable[2:length(TAGtable)])/length(codons)*100#percent of sequences containing TAG

#this creates a matrix of amino acid sequences that do not contain TAG codons
TAGpos <- which(AAs == "TAG")
TAGrow <- TAGpos%%nrow(AAs)
AAsnoTAG <- AAs[-TAGrow,]

#this creates heatmap for amino acid frequency per library position, change scale according to values
AAtable <- apply(AAs,2,function(x) table(factor(x,levels=aa)))
AAtable <- as.matrix(AAtable/length(codons))
colnames(AAtable) <- c(1:lib)
heatmapcolors <- colorRampPalette(brewer.pal(9,"Blues"))(100)
sc <- seq(0.0,0.6,by=0.006)
AAheatmap <- heatmap.2(AAtable, Rowv = NA, Colv = NA, col = heatmapcolors, density.info = "none", scale = "none", trace = "none", breaks = sc,  xlab = "Position in Library", ylab = "Codon", margins = c(3,4), dendrogram = "none")

#this creates projected heatmap based on NNK randomized codons
randomAAs <- matrix(0,21,lib,dimnames = list(rownames(AAtable),c(1:lib)))
randomAAs[c("A","G","P","T","V"),] <- 2/32
randomAAs[c("C","H","Q","N","K","Y","D","E","W","I","M","TAG","F"),] <- 1/32
randomAAs[c("L","S","R"),] <- 3/32
NNKheatmap <- heatmap.2(randomAAs, Rowv = NA, Colv = NA, col = heatmapcolors, density.info = "none", scale = "none", trace = "none", breaks = sc,  xlab = "Position in Library", ylab = "Codon", margins = c(3,4), dendrogram = "none")
```

```
#this creates heatmap showing bias from random, change scale with respect to range of values
lscale <- seq(-1,4,by=5/100)
librarybias <- (AAtable - randomAAs)/randomAAs
Biasheatmap <- heatmap.2(librarybias, Rowv = NA, Colv = NA, col = heatmapcolors, density.info =
"none", scale = "none", trace = "none", breaks = lscale,  xlab = "Position in Library", ylab = "Codon",
margins = c(3,4), dendrogram = "none")
librarybias <- as.data.frame(librarybias)

#this creates heatmap for AAsnoTAG
AAnoTAGtable <- apply(AAsnoTAG,2,function(x) table(factor(x,levels=aa)))
AAnoTAGtable <- as.matrix(AAnoTAGtable/nrow(AAsnoTAG))
colnames(AAnoTAGtable) <- c(1:lib)
heatmapcolors <- colorRampPalette(brewer.pal(9,"Blues"))(100)
sc <- seq(0.0,0.3,by=0.003)
AAheatmap <- heatmap.2(AAnoTAGtable, Rowv = NA, Colv = NA, col = heatmapcolors, density.info =
"none", scale = "none", trace = "none", breaks = sc,  xlab = "Position in Library", ylab = "Codon", margins
= c(3,4), dendrogram = "none")

#writes csv files for uniqueAAs and bias heatmaps change path to make file
path<-"C:/Users/demon//Documents//NGS/F101/23316Wns_F101-R1_S4_L001_R1_001.fastq"
write.csv(UniqueAAs, paste(path,"/12mernegnegUniqueAAs.csv", sep = ""), row.names = F)
write.csv(TAGtable, paste(path,"/12mernegnegTAGtable.csv", sep = ""), row.names = T)
write.csv(librarybias, paste(path,"/12mernegnegLibraryBias.csv", sep = ""), row.names = T)
```

Supplementary Script 2: Mutant Round 3 Statistical Analysis

```
library(prodlim)
library(VennDiagram)
library(ggplot2)
library(ggrepel)
library(plotly)
ncAA <- "q"
lib <- 14
path <- "C:/Users/demon//PEPTIDE/NGS"

#Reads .csv files of UniqueAAs from Amino Acid Analysis and combines them into one dataframe with
percentages from each round
F85 <- read.csv("C:/Users/demon/PEPTIDE/NGS/F30/F30R3.csv", header = TRUE)
F156 <- read.csv("C:/Users/demon/PEPTIDE/NGS/F101/F101R3.csv")
F217 <- read.csv("C:/Users/demon/PEPTIDE/NGS/F162/F162R3.csv")

F85[F85 == "TAG"] <- ncAA
F156[F156 == "TAG"] <- ncAA
F217[F217 == "TAG"] <- ncAA
F85 <- data.frame(apply(F85[,1:lib], 1, paste, collapse = ""), F85[,lib+1])
F156 <- data.frame(apply(F156[,1:lib], 1, paste, collapse = ""), F156[,lib+1])
F217 <- data.frame(apply(F217[,1:lib], 1, paste, collapse = ""), F217[,lib+1])
colnames(F85) <- c("Sequence", "nF85")
colnames(F156) <- c("Sequence", "nF156")
colnames(F217) <- c("Sequence", "nF217")
a <- row.match(F85[1], F156[1])
```

```
b <- row.match(F85[1], F217[1])
d <- row.match(F156[1], F85[1])
e <- row.match(F156[1], F217[1])
g <- row.match(F217[1], F85[1])
h <- row.match(F217[1], F156[1])
CombinedData <- F85
colnames(CombinedData)[2] <- "nF85"
CombinedData$nF156 <- F156[a,2]
CombinedData$nF217 <- F217[b,2]
F156$nF85 <- F85[d,2]
F156$nF217 <- F217[e,2]
F217$nF85 <- F85[g,2]
F217$nF156 <- F156[h,2]
CombinedData <- merge(CombinedData, F156, all.x = TRUE, all.y = TRUE)
CombinedData <- merge(CombinedData, F217, all.x = TRUE, all.y = TRUE)
CombinedData[is.na(CombinedData)] <- 0
CombinedData$percentF85 <- CombinedData$nF85/sum(CombinedData$nF85)*100
CombinedData$percentF156 <- CombinedData$nF156/sum(CombinedData$nF156)*100
CombinedData$percentF217 <- CombinedData$nF217/sum(CombinedData$nF217)*100
CombinedData <- CombinedData[order(-CombinedData$nF217),]

#Addition of Log2FoldChange to CombinedPlot, assumes 0.5 sequence number for sequences that are not
found in a round
Log2Data <- CombinedData[,1:4]
Log2Data[Log2Data == 0] <- 0.5
Log2Data$percentF85 <- Log2Data$nF85/sum(CombinedData$nF85)*100
Log2Data$percentF156 <- Log2Data$nF156/sum(CombinedData$nF156)*100
Log2Data$percentF217 <- Log2Data$nF217/sum(CombinedData$nF217)*100
Log2Data$Log2F217vF85 <- log2(Log2Data$percentF217/Log2Data$percentF85)
Log2Data$Log2F217vF156 <- log2(Log2Data$percentF217/Log2Data$percentF156)
Log2Data$Log2F156vF85 <- log2(Log2Data$percentF156/Log2Data$percentF85)

#Finding Common Sequences
Common <- CombinedData
Common[Common == 0] <- NA
Common <- na.omit(Common)

totalpercentCommon <- sum(Common[,5:7])/3

#F85andF156Shared
F85andF156 <- CombinedData
test <- F85andF156$nF85 == 0 | F85andF156$nF156 == 0
F85andF156 <- F85andF156[which(test==FALSE),]

totalpercentF85andF156 <- sum(F85andF156[,5:7])/3

#F85andF156Only
F85andF156only <- F85andF156
test <- F85andF156$nF217 == 0
F85andF156only <- F85andF156[which(test==TRUE),]
```

```
totalpercentF85andF156only <- sum(F85andF156only[,5:7])/3

#F85andF217Shared
F85andF217 <- CombinedData
test <- F85andF217$nF85 == 0 | F85andF217$nF217 == 0
F85andF217 <- F85andF217[which(test==FALSE),]

totalpercentF85andF217 <- sum(F85andF217[,5:7])/3

#F85andF217Only
F85andF217only <- F85andF217
test <- F85andF217$nF156 == 0
F85andF217only <- F85andF217[which(test==TRUE),]

totalpercentF85andF217only <- sum(F85andF217only[,5:7])/3

#F156andF217Shared
F156andF217 <- CombinedData
test <- F156andF217$nF156 == 0 | F156andF217$nF217 == 0
F156andF217 <- F156andF217[which(test==FALSE),]

totalpercentF156andF217 <- sum(F156andF217[,5:7])/3

#F156and217only
F156andF217only <- F156andF217
test <- F156andF217$nF85 == 0
F156andF217only <- F156andF217only[which(test==TRUE),]

totalpercentF156andF217only <- sum(F156andF217only[,5:7])/3

#F85Unique
F85Unique <- CombinedData
test <- F85Unique$nF217 == 0 & F85Unique$nF156 == 0
F85Unique <- F85Unique[which(test==TRUE),]

totalpercentF85Unique <- sum(F85Unique[,5:7])/3

#F156Unique
F156Unique <- CombinedData
test <- F156Unique$nF217 == 0 & F156Unique$nF85 == 0
F156Unique <- F156Unique[which(test==TRUE),]

totalpercentF156Unique <- sum(F156Unique[,5:7])/3

#F217Unique
F217Unique <- CombinedData
test <- F217Unique$nF156 == 0 & F217Unique$nF85 == 0
F217Unique <- F217Unique[which(test==TRUE),]

totalpercentF217Unique <- sum(F217Unique[,5:7])/3
```

```
#Calculates total reads for all 3 samples
totalreads <- sum(CombinedData[,2:4])
F85total <- sum(CombinedData$nF85)
F156total <- sum(CombinedData$nF156)
F217total <- sum(CombinedData$nF217)


#Calculates Peptides > 0.1% for all samples
F85morethan0.1 <- length(which(CombinedData$percentF85>0.1))
F156morethan0.1 <- length(which(CombinedData$percentF156>0.1))
F217morethan0.1 <- length(which(CombinedData$percentF217>0.1))

#Calculates ratios for round3 enrichment plots
F85_10k <- length(which(CombinedData$nF85>10000))
F85_1k <- length(which(CombinedData$nF85>999 & CombinedData$nF85 < 10000))
F85_100 <- length(which(CombinedData$nF85>99 & CombinedData$nF85 < 1000))
F85_10 <- length(which(CombinedData$nF85>9 & CombinedData$nF85 < 100))
F85_1 <- length(which(CombinedData$nF85>0.9 & CombinedData$nF85 < 10))
percent10k85 <- sum(CombinedData[which(CombinedData$nF85>10000),"percentF85"])
percent1k85 <- sum(CombinedData[which(CombinedData$nF85>999 & CombinedData$nF85 <
10000),"percentF85"])
percent100_85 <- sum(CombinedData[which(CombinedData$nF85>99 & CombinedData$nF85 <
1000),"percentF85"])
percent10_85 <- sum(CombinedData[which(CombinedData$nF85>9 & CombinedData$nF85 <
100),"percentF85"])
percent1_85 <- sum(CombinedData[which(CombinedData$nF85>0.9 & CombinedData$nF85 <
10),"percentF85"])


F156_10k <- length(which(CombinedData$nF156>10000))
F156_1k <- length(which(CombinedData$nF156>999 & CombinedData$nF156 < 10000))
F156_100 <- length(which(CombinedData$nF156>99 & CombinedData$nF156 < 1000))
F156_10 <- length(which(CombinedData$nF156>9 & CombinedData$nF156 < 100))
F156_1 <- length(which(CombinedData$nF156>0.9 & CombinedData$nF156 < 10))
percent10k156 <- sum(CombinedData[which(CombinedData$nF156>10000),"percentF156"])
percent1k156 <- sum(CombinedData[which(CombinedData$nF156>999 & CombinedData$nF156 <
10000),"percentF156"])
percent100_156 <- sum(CombinedData[which(CombinedData$nF156>99 & CombinedData$nF156 <
1000),"percentF156"])
percent10_156 <- sum(CombinedData[which(CombinedData$nF156>9 & CombinedData$nF156 <
100),"percentF156"])
percent1_156 <- sum(CombinedData[which(CombinedData$nF156>0.9 & CombinedData$nF156 <
10),"percentF156"])


F217_10k <- length(which(CombinedData$nF217>10000))
F217_1k <- length(which(CombinedData$nF217>999 & CombinedData$nF217 < 10000))
F217_100 <- length(which(CombinedData$nF217>99 & CombinedData$nF217 < 1000))
F217_10 <- length(which(CombinedData$nF217>9 & CombinedData$nF217 < 100))
F217_1 <- length(which(CombinedData$nF217>0.9 & CombinedData$nF217 < 10))
percent10k217 <- sum(CombinedData[which(CombinedData$nF217>10000),"percentF217"])
```

```
percent1k217 <- sum(CombinedData[which(CombinedData$nF217>999 & CombinedData$nF217 <
10000),"percentF217"])
percent100_217 <- sum(CombinedData[which(CombinedData$nF217>99 & CombinedData$nF217 <
1000),"percentF217"])
percent10_217 <- sum(CombinedData[which(CombinedData$nF217>9 & CombinedData$nF217 <
100),"percentF217"])
percent1_217 <- sum(CombinedData[which(CombinedData$nF217>0.9 & CombinedData$nF217 <
10),"percentF217"])


#Creates Venn Diagram of Peptides, see https://www.datanovia.com/en/blog/venn-diagram-with-r-or-
rstudio-a-million-ways/ and https://www.sthda.com/english/wiki/colors-in-r
peptidelist <- list(A = F85[,1], B = F156[,1], C = F217[,1])
display_venn <- function(x, ...){
  library(VennDiagram)
  grid.newpage()
  venn_object <- venn.diagram(x, filename = NULL, ...)
  grid.draw(venn_object)
}
display_venn(
  peptidelist,
  category.names = c("F85" , "F156" , "F217"),
  # Circles
  lwd = 2,
  lty = 'blank',
  fill = c("blue", "yellow", "red"),
  # Numbers
  cex = .9,
  fontface = 4,
  # Set names
  cat.cex = 1,
  cat.fontface = "bold",
  cat.default.pos = "outer"
)

#Makes Enrichment plot for F85
df <- data.frame(bin=c("1-9","10-99","100-999","1000-9999","10000+"))
df$sumpercent <- c(percent1_85,percent10_85,percent100_85,percent1k85,percent10k85)
df$unique <- c(F85_1,F85_10,F85_100,F85_1k,F85_10k)
df$y <- cumsum(df$sumpercent)
F85EnrichmentSummary <- df

ggplot(df, aes(ymin = 0, ymax = 100, xmin = 0, xmax = 10000, fill = bin)) +
  geom_rect(mapping = aes(xmin = 0, xmax = unique, ymin = y-sumpercent, ymax = y)) +
  scale_x_log10() +
  scale_fill_manual(values = c("#CCCCCC", "#ADD8E6", "#6495ED", "#4169E1","#000066")) # Light,
medium, dark blue

#Makes Enrichment plot for F156
df <- data.frame(bin=c("1-9","10-99","100-999","1000-9999","10000+"))
df$sumpercent <- c(percent1_156,percent10_156,percent100_156,percent1k156,percent10k156)
```

```
df$unique <- c(F156_1,F156_10,F156_100,F156_1k,F156_10k)
df$y <- cumsum(df$sumpercent)
F156enrichmentsummary <- df

ggplot(df, aes(ymin = 0, ymax = 100, xmin = 0, xmax = 10000, fill = bin)) +
  geom_rect(mapping = aes(xmin = 0, xmax = unique, ymin = y-sumpercent, ymax = y)) +
  scale_x_log10() +
  scale_fill_manual(values = c("#CCCCCC", "#ADD8E6", "#6495ED", "#4169E1","#000066")) # Light,
medium, dark blue

#Makes Enrichment plot for F217
df <- data.frame(bin=c("1-9","10-99","100-999","1000-9999","10000+"))
df$sumpercent <- c(percent1_217,percent10_217,percent100_217,percent1k217,percent10k217)
df$unique <- c(F217_1,F217_10,F217_100,F217_1k,F217_10k)
df$y <- cumsum(df$sumpercent)
F217EnrichmentSummary <- df

ggplot(df, aes(fill = bin)) +
  geom_rect(mapping = aes(xmin = 0, xmax = unique, ymin = y-sumpercent, ymax = y)) +
  scale_x_log10() +
  scale_fill_manual(values = c("#CCCCCC", "#ADD8E6", "#6495ED", "#4169E1","#000066")) # Light,
medium, dark blue

# Assign colors based on conditions
Log2Data$color <- ifelse(Log2Data$nF85 == 0.5 & Log2Data$nF156 == 0.5, "red",
                ifelse(Log2Data$nF156 == 0.5 & Log2Data$nF217 == 0.5, "blue",
                    ifelse(Log2Data$nF217 == 0.5 & Log2Data$nF85 == 0.5, "yellow",
                        ifelse(Log2Data$nF85 != 0.5 & Log2Data$nF156 != 0.5 & Log2Data$nF217 ==
0.5, "green",
                            ifelse(Log2Data$nF85 != 0.5 & Log2Data$nF156 == 0.5 & Log2Data$nF217
!= 0.5, "purple",
                                ifelse(Log2Data$nF85 == 0.5 & Log2Data$nF156 != 0.5 &
Log2Data$nF217 != 0.5, "orange", "black")
                            )))))  # Default color for other points
topF85colors <- table(Log2Data[which(Log2Data$percentF85 > 0.1),"color"])
topF156colors <- table(Log2Data[which(Log2Data$percentF156 > 0.1),"color"])
topF217colors <- table(Log2Data[which(Log2Data$percentF217 > 0.1),"color"])

# Generate scatter plot with log-transformed axes, change data and axes as needed
ggplot(Log2Data, aes(x = percentF156, y = percentF217, color = color)) +
  geom_point(size = 1.5, alpha = 0.7) +  # Scatter points
  # Add dashed reference lines at 0.1 (1x10^-1)
  geom_vline(xintercept = 1e-1, linetype = "dashed", color = "black", linewidth = 0.6) +
  geom_hline(yintercept = 1e-1, linetype = "dashed", color = "black", linewidth = 0.6) +
  scale_color_identity() +
  scale_x_log10() +
  scale_y_log10() +
  labs(
    title = "Round 3 Sequence % Abundance",
    x = "F156 %",
    y = "F217 %"
```

```
  ) +
  theme_minimal() +
  theme(
    axis.line = element_line(linewidth = 1.2, color = "black"),  # updated from size → linewidth
    axis.text = element_text(size = 12),
    axis.title = element_text(size = 14, face = "bold"),
    panel.grid.major = element_line(color = "gray90"),
    panel.grid.minor = element_blank()
  )


#write csv files for log2Data and CombinedData
write.csv(CombinedData, paste(path, "/CombinedData.csv", sep = ""), row.names = F)
write.csv(Log2Data, paste(path, "/Log2Data.csv", sep = ""), row.names = F)
```
